# Supplementary material for: PHOENIX (Picking up Hidden Osteoporosis Effectively during Normal CT Imaging without additional X-rays): protocol for a randomised, multicentre feasibility study
Source: BMJ Open. 2022 May 20;12(5):e050343. doi: 10.1136/bmjopen-2021-050343 (PMC9125739; doi:10.1136/bmjopen-2021-050343)
Supplement: Supplementary data [file bmjopen-2021-050343supp001.pdf]

Appendix 1

NHS
Cambridge University Hospitals
NHS Foundation Trust

UNIVERSITY OF CAMBRIDGE

PHOENIX
Picking up Hidden Osteoporosis Effectively during Normal CT Imaging without additional X-rays

Osteoporosis is a condition that makes bones become fragile and break more easily, especially in the spine and hips. Osteoporosis can be treated. We would like to diagnose it earlier and are running a study called PHOENIX-f to see if this can be done using the scan that you are having today. There are no extra scans, other tests or treatment required to join the study.

If you are interested in being part of the study, please turn over the page and read on

If you do not wish to take part in the study, can you please tell us why in the box below? This would be really useful for us to know, but you do not have to tell us.

Now please return this form to the PHOENIX-f returns box

Thank you for your co-operation

Poole KES, *et al. BMJ Open* 2022; 12:e050343. doi: 10.1136/bmjopen-2021-050343

By completing this form, we understand that you are happy for one of our research team to talk to you about the study.

Your scan taken today can also be used to measure your bone health.

We can't use every scan, so please answer the following 3 questions to see if we can use yours.

Please circle your answer

**Do you take medication for your bones once a week that require you to sit or stand upright?**	**Y**	**N**
**Do you have a yearly infusion (drip) or injection for your bones?**	**Y**	**N**
**Do you have a hip replacement? Have you ever had a hip operation?**	**Y**	**N**

If you answered 'Yes' to any of the questions above unfortunately we will be unable to use your CT scan today for PHOENIX-f.

Please return this form to the PHOENIX-f returns box.
Thank you for your co-operation.

If you have answered 'NO' to ALL the questions above we will be able to review your CT scan for PHOENIX-f.

In order to understand the study more, please read the patient information leaflet. Then, if you are interested in taking part in PHOENIX to measure your bone health, please fill in the questionnaire overleaf and complete the consent form

PHOENIX-f Consent Form

Chief Investigator: Dr Ken Poole, Consultant in Rheumatology and Metabolic Bone Disease, Cambridge

Please *initial* boxes below, then sign and date the form and add your address ***Initials***

1. I confirm that I have read and understood the patient information for the study. ☐
2. I understand that my participation is voluntary and that I am free to withdraw at any time without giving any reason, without my medical care or legal rights being affected. ☐
3. If I decide to withdraw from the trial I agree to data already collected to be used for Phoenix-f. ☐
4. I give permission for the study researchers to have access to my scan and relevant NHS databases in order to conduct this research ☐
5. I agree for my personal details and relevant medical information to be sent from my hospital to the study team at Addenbrooke's Hospital for the purpose of the research. I understand that scans have identifiable data and will be kept securely at Addenbrooke's Hospital and will only be accessed by the research team ☐
6. I agree to the research team contacting my GP to notify them of my participation in the study and any results ☐
7. I give permission to be contacted by post or phone by the study team at Addenbrooke's Hospital. I understand that this will be to send me 2 questionnaires and may include an invitation to talk to a researcher about my experience of the study ☐
8. I agree to take part in the above study. ☐

Mr / Mrs / Ms / Dr (please circle as appropriate)

....
Please enter first and family name above

……………………………………………………………. *Date:* ……………………………….
Your Signature

Date of Birth: .... *Height:* .... *Weight:* ....

NHS number: ....

Why are you having a scan today? ....

The following questions are required for accurate bone health calculation:

Your country of origin: .... *Ethnicity:* ....

PLEASE TURN OVER PAGE TO COMPLETE THIS FORM

PHOENIX Methodology paper_2021_04_29_ submission 4

**BONE HEALTH QUESTIONNAIRE**	**Yes**	**No**	**Not Sure**
Have you broken any bones since you turned 40?			
If Yes, which bones?			
Did your mother or father ever break a hip?			
Do you currently smoke?			
Do you take steroid tablets now (eg: 'prednisolone'), or have you taken steroid tablets in the past, for longer than three months at any time?			
Have you been diagnosed with rheumatoid arthritis?			
Have you been diagnosed with any of the following?<br>**(Tick 'yes' if any of the following apply)**<br>• Premature menopause (before 45yrs of age)<br>• Insulin dependent diabetes<br>• Brittle bone disease, Ankylosing spondylitis<br>• Overactive thyroid<br>• Low testosterone level (men only)<br>• Chronic malnutrition or malabsorption eg: anorexia or coeliac disease<br>• Chronic liver disease			
Do you drink three or more units of alcohol a **day**, on average? (That's 1 1/2 pints of beer or 3 glasses of wine or 3 short measures of spirits)			

Your Address (for questionnaires):

....

....

....

....

Post Code: .... **Your Telephone no:** ....

Your GP Name and address:

....

....

Now please return this form to the PHOENIX-f returns box. Thank you for your co-operation

PHOENIX Methodology paper_2021_04_29_ submission 4
