# Supplementary material for: PHOENIX (Picking up Hidden Osteoporosis Effectively during Normal CT Imaging without additional X-rays): protocol for a randomised, multicentre feasibility study
Source: BMJ Open. 2022 May 20;12(5):e050343. doi: 10.1136/bmjopen-2021-050343 (PMC9125739; doi:10.1136/bmjopen-2021-050343)
Supplement: Supplementary data [file bmjopen-2021-050343supp002.pdf]

PHOENIX-f Clinical Decision Tree *part 1*

Initial steps

1. Retrieve and visually inspect CT images for any vertebral fractures with AP and lateral localiser views (using Mindways QCT PRO SlicePick for 6-point morphometry).

2. Measure CTXA Hip and/or Spine BMD with QCT PRO, noting Femoral neck & Hip BMD results (in g/cm2) and/or Average L1-L3 Spine BMD results (in mg/cm3).

3. Recalculate FRAX online using the Mindways Femoral neck/Hip BMD (g/cm2) value & change 'Previous Fracture' to 'Yes' if a Grade 2 or 3 fracture is found at step 1.

4. If only one (Hip or Spine) result is available, follow the GP Advice Clinical Decision Tree here to generate automatic GP Advice text.

5. If both Hip BMD and Spine BMD results are available, CTXA Femoral neck/Hip results usually dominate, and the following generic Spine 3D QCT GP advice box text is automatically added, "See CTXA Hip Bone Mineral Densitometry report for clinical interpretation." In GP Advice box add the Spine BMD value and definitions of Normal/ Osteopaenia/Osteoporosis.

6. Only if Spine BMD value is <80mg/cm3 AND NOGG advice is GREEN (after entering Femoral neck/Hip BMD into FRAX) does a Spine result dominate. In this case, the following generic CTXA Hip GP advice box text is automatically added, "Both hip and spine results are available. For this patients' clinical interpretation, please see their associated Spine 3D QCT BMD result".

GP advice text using *Mindways QCT Pro Spine L1-3 BMD value + initial FRAX score/NOGG*

ACR Spine BMD Thresholds

**Abnormal High**	**Normal**		**Osteopenia**		**Osteoporosis**
**BMD >200mg/cm^3**	**BMD >120mg/cm^3**		**120mg/cm^3 ≥ BMD > 80mg/cm^3**		**BMD ≤ 80mg/cm^3**
	**Initial FRAX Major osteoporotic fracture risk ≤ 20%**	**Initial FRAX Major osteoporotic fracture risk >20%**	**Initial FRAX Major osteoporotic fracture risk ≤ 20%**	**Initial FRAX Major osteoporotic fracture risk >20%**	
This patient had a FRAX score high enough to warrant a BMD assessment which was done using the clinical CT scan. Their BMD of (***) mg/cm3 (*) is higher than expected. High density can be due to degenerative/ genetic effects or for other reasons.	This patient had a FRAX score high enough to warrant a BMD assessment which was done using the clinical CT scan. Their spine BMD >120mg/cm3 (*) is defined as normal bone density by ACR criteria. Unless there is a change in the clinical circumstances, no routine repeat BMD tests are indicated.	This patient had a FRAX score high enough to warrant BMD assessment using the clinical CT scan. Their spine BMD >120mg/cm3 (*) which is defined as normal bone density by ACR criteria. However, there are clinical risk factors which elevate the 10 year future fracture risk (**%).	This patient had a FRAX score high enough to warrant a BMD assessment which was done using the clinical CT scan. Their SPINE BMD of 80-120 mg/cm3 (*) is defined as osteopenia by ACR criteria. There is no current indication for bone active therapy, but the patient may wish to follow the lifestyle advice to maintain bone density (if relevant to patient avoid smoking and excess alcohol intake, and maintain weightbearing exercise.) Please use the online WHO FRAX tool to determine if a standard DXA scan is needed in 3 years.	This patient had a FRAX score high enough to warrant BMD assessment using the clinical CT scan. Their SPINE BMD of 80-120 mg/cm3(*) is defined as osteopenia by ACR criteria. However, there are clinical risk factors which elevate the 10 year future fracture risk above the treatment threshold (**%). Unless contraindicated, consider bisphosphonate treatment.	This patient had a FRAX score high enough (10 year future risk **%) to warrant a BMD assessment using their clinical CT scan. Their SPINE BMD of <80mg/cm3 (*) is defined as osteoporosis by ACR criteria. We suggest investigations to rule out secondary causes (e.g. renal/liver/bone function, low vit D, high PTH, occasionally TTG, paraprotein screen). Then, unless contraindicated, consider oral or intravenous bisphosphonates as first line therapy with sufficient vitamin D & calcium. If relevant to patient, please recommend avoid smoking, excess alcohol intake, & maintain weightbearing exercise. Follow up: Please re-assess fracture risk and indication for treatment after 3-5 years
If grade 2 or 3 vertebral # identified.<br>Include:<br>Please note that a vertebral fracture was identified. Unless this was traumatic/already known, you may wish to investigate further	If grade 2 or 3 vertebral # identified.<br>Include:<br>Please note that a vertebral fracture was identified. Unless this was traumatic/already known, you may wish to investigate further	If grade 2 or 3 vertebral # identified.<br>Include:<br>Please note that a vertebral fracture was identified. Unless this was traumatic/already known, you may wish to investigate further	If grade 2 or 3 vertebral # identified.<br>Include:<br>Please note that a vertebral fracture was identified. Unless this was traumatic/already known, you may wish to investigate further	If grade 2 or 3 vertebral # identified.<br>Include:<br>Please note that a vertebral fracture was identified. Unless this was traumatic/already known, you may wish to investigate further	If grade 2 or 3 vertebral # identified.<br>Include:<br>Please note that a vertebral fracture was identified. Unless this was traumatic/already known, you may wish to investigate further

* Vertebrae analysed

** Major Fracture Risk Score

*** Average BMD value of vertebrae analysed

American College of Radiology

BMJ Publishing Group Limited (BMJ) disclaims all liability and responsibility arising from any reliance placed on this supplemental material which has been supplied by the author(s)

PHOENIX-f Clinical Decision Tree *part 2*

GP advice text using *Mindways QCT Pro CTXA Femoral neck BMD value + BMD-Adjusted FRAX/NOGG*

WHO BMD T-Score Criteria								
**Abnormal High T-Score > 2**	**Normal T-Score > -1**			**Osteopenia -1 ≥ T-Score > -2.5**			**Osteoporosis T-Score ≤ -2.5**	
**Any FRAX/NOGG Threshold**	**Below FRAX /NOGG Treatment Threshold**	**Below FRAX/NOGG Treatment Threshold AND ≥75yrs with prior fragility#/vert #**	**Above FRAX/NOGG Treatment Threshold**	**Below FRAX/NOGG Treatment Threshold**	**Either i) Below FRAX/NOGG Treatment Threshold ≥75yrs with prior fragility#/vert# OR ii) Between -2.0 & -2.50 with prior fragility#/vert#**	**Above FRAX/NOGG Treatment Threshold**	**Below FRAX/NOGG Treatment Threshold**	**Either i) Above FRAX/NOGG Treatment Threshold OR ii) Below treatment threshold with a prior fracture aged ≥75**
This patient has a FRAX score high enough to warrant a BMD assessment which was done using their clinical CT scan. Their *femoral neck/ total hip T-score was (***) which is higher than expected which can have genetic, degenerative or other causes. There is no evidence of osteoporosis	This patient had a FRAX score high enough to warrant a BMD assessment using the clinical CT scan. Their *femoral neck/total hip T-score was >-1 which is defined as normal hip bone density by WHO guidelines (FRAX score **%). Unless there is a change in the clinical circumstances, no routine repeat BMD tests are indicated.	This patient had a FRAX score high enough to warrant a BMD assessment using the clinical CT scan. Their *femoral neck/ total hip T-score was >-1 which is defined as normal hip bone density by WHO guidelines (FRAX score **%). Please note National Guidelines recommend bisphosphonate treatment in the majority of elderly people with a prior fragility fracture regardless of calculated risk. If relevant to patient, recommend avoid smoking, excess alcohol intake, & maintain weightbearing exercise.	This patient had a FRAX score high enough to warrant a BMD assessment using the clinical CT scan. Their *femoral neck/total hip T-score was >-1 which is defined as normal hip bone density by WHO guidelines. However, there are clinical risk factors which elevate the 10 year future fracture risk (FRAX score **%).	This patient had a FRAX score high enough to warrant BMD assessment using the clinical CT scan. Their *femoral neck/ total hip T-score was between -1 and -2.5 which is defined as osteopenia, or low normal bone density, by WHO guidelines. (FRAX score **%). There is no current indication for bone active therapy, but the patient may wish to follow the lifestyle advice to maintain bone density (if relevant to patient avoid smoking and excess alcohol intake, and maintain weightbearing exercise.) Please use the online WHO FRAX tool to determine if a standard DXA scan is needed in 3 years.	This patient had a FRAX score high enough to warrant BMD assessment using the clinical CT scan. Their *femoral neck/total hip T-score was between -1 and > -2.5 which is defined as osteopenia, or low normal bone density, by WHO guidelines (FRAX Score **%). Please note National Guidelines recommend bisphosphonate treatment in the majority of older people with a prior fragility fracture regardless of calculated risk. Unless contraindicated consider bisphosphonate treatment. If relevant to patient, recommend avoid smoking, excess alcohol intake, & maintain weightbearing exercise.	This patient had a FRAX score high enough to warrant BMD assessment using the clinical CT scan. Their *femoral neck/total hip T-score was between -1 and > -2.5 which is defined as osteopenia, or low normal bone density, by WHO guidelines. However, there are clinical risk factors which elevate the 10 year future fracture risk above the treatment threshold (FRAX Score **%). Unless contraindicated consider bisphosphonate treatment. If relevant to patient, recommend avoid smoking, excess alcohol intake, & maintain weightbearing exercise.	This patient's FRAX score was high enough to warrant BMD assessment using their clinical CT scan. Their *femoral neck/total hip T-score was osteoporotic (FRAX score **%). While the scan indicates osteoporosis at the hip, treatment is not automatically recommended (by National Guidelines) at this level of 10-year risk. Suggest investigations to rule out secondary causes (e.g renal/liver/bone function, occasionally TTG, paraprotein screen) The patient may wish to follow the lifestyle advice to maintain bone density (if relevant to patient avoid smoking and excess alcohol intake, and maintain weightbearing exercise.)	This patient's FRAX score was high enough to warrant BMD assessment using their clinical CT scan. Their *femoral neck/ total hip T-score was osteoporotic (FRAX score **%). Suggest investigations to rule out secondary causes (e.g renal/liver/ bone function, occasionally TTG, paraprotein screen). Then, unless contraindicated, consider oral or intravenous bisphosphonates as first line therapy with sufficient vitamin D & calcium. Follow up: Please reassess fracture risk and indication for treatment after 3-5 years
If grade 2 or 3 vertebral # identified<br>Include:<br>Please note that a vertebral fracture was identified, unless this was traumatic/already known, you may wish to investigate further	If grade 2 or 3 vertebral # identified, <75y/o only.<br>Include:<br>Please note that a vertebral fracture was identified, unless this was traumatic/ already known, you may wish to order a spine only DXA scan.	If grade 2 or 3 vertebral # identified.<br>Include:<br>Please note that a vertebral fracture was identified.	If grade 2 or 3 vertebral # identified, <75y/o only.<br>Include:<br>Please note that a vertebral fracture was identified, unless this was traumatic/ already known, you may wish to order a spine only DXA scan."	If grade 2 or 3 vertebral # identified, <75y/o only.<br>Include:<br>Please note that a vertebral fracture was identified, unless this was traumatic/already known, you may wish to order a spine only DXA scan.	If grade 2 or 3 vertebral # identified<br>Include:<br>Please note that a vertebral fracture was identified.	If grade 2 or 3 vertebral # identified<br>Include:<br>Please note that a vertebral fracture was identified.	If grade 2 or 3 vertebral # identified, <75y/o only<br>Include:<br>Please note that a vertebral fracture was identified.	If grade 2 or 3 vertebral # identified<br>Include:<br>Please note that a vertebral fracture was identified.

* Where lowest BMD T-Score was at either the Femoral Neck or Total Hip

** Value of either the Major FRAX score OR the Hip Fracture FRAX score when this value is above the FRAX/NOGG treatment threshold AND Major is below the threshold.

*** Value of the BMD T-Score
