# Supplementary material for: PHOENIX (Picking up Hidden Osteoporosis Effectively during Normal CT Imaging without additional X-rays): protocol for a randomised, multicentre feasibility study
Source: BMJ Open. 2022 May 20;12(5):e050343. doi: 10.1136/bmjopen-2021-050343 (PMC9125739; doi:10.1136/bmjopen-2021-050343)
Supplement: Author's manuscript [file bmjopen-2021-050343.draft_revisions.pdf]

BMJ Open

BMJ Open is committed to open peer review. As part of this commitment we make the peer review history of every article we publish publicly available.

When an article is published we post the peer reviewers' comments and the authors' responses online. We also post the versions of the paper that were used during peer review. These are the versions that the peer review comments apply to.

The versions of the paper that follow are the versions that were submitted during the peer review process. They are not the versions of record or the final published versions. They should not be cited or distributed as the published version of this manuscript.

BMJ Open is an open access journal and the full, final, typeset and author-corrected version of record of the manuscript is available on our site with no access controls, subscription charges or pay-per-view fees (http://bmjopen.bmj.com).

If you have any questions on BMJ Open's open peer review process please email info.bmjopen@bmj.com

BMJ Open

CLINICAL STUDY PROTOCOL PHOENIX-f (Feasibility Study): Picking up Hidden Osteoporosis Effectively during Normal CT Imaging without additional X-rays

Journal:	*BMJ Open*
Manuscript ID	bmjopen-2021-050343
Article Type:	Protocol
Date Submitted by the Author:	10-May-2021
Complete List of Authors:	Poole, Kenneth; University of Cambridge, Metabolic Bone Unit, Box 201a, Level 7, Addenbrooke's Hospital, Hills Rd CB2 0QQ; Cambridge University Hospitals NHS Foundation Trust, Rheumatology<br>Chappell, Daniel; University of Cambridge, Rheumatology<br>Clark, Emma; University of Bristol, Clinical Science at North Bristol<br>Fleming, Jane; University of Cambridge, Cambridge Institute of Public Health; University of Cambridge, Department of Public Health and Primary Care<br>Kaptoge, Stephen; University of Cambridge<br>Shepstone, Lee; University of East Anglia<br>Turmezei, Tom; Cambridge University Hospitals NHS Foundation Trust; Norfolk and Norwich University Hospital NHS Trust<br>Wagner, Adam; NIHR Collaboration for Leadership in Applied Health Research & Care (CLAHRC) East of England; University of East Anglia, Norwich Medical School<br>willoughby, karen; University of Cambridge School of Clinical Medicine, Medicine
Keywords:	Bone diseases < ORTHOPAEDIC & TRAUMA SURGERY, Hip < ORTHOPAEDIC & TRAUMA SURGERY, Spine < ORTHOPAEDIC & TRAUMA SURGERY

SCHOLARONE™
Manuscripts

CLINICAL STUDY PROTOCOL

PHOENIX-f (Feasibility Study): **P**icking up **H**idden **O**steoporosis **E**ffectively during **N**ormal CT **I**maging without additional **X**-rays

A multi-centred, randomised, pragmatic feasibility study comparing health outcomes in three parallel- groups

This project is funded by the National Institute for Health Research (NIHR) under its Research for Patient Benefit (RfPB) Programme (Grant Reference Number PB-PG-0816-20027). The views expressed are those of the author(s) and not necessarily those of the NIHR or the Department of Health and Social Care

Corresponding author: Kenneth Poole a, b,
Protocol authors:
Kenneth Poole a, b, Daniel Chappell a, Emma M Clark c, Jane Fleming b, Dr Stephen Kaptoge b, Lee Shepstone d, Tom Turmezei a, e Adam P Wagner d, f, Karen Willoughby b

a Cambridge University Hospitals NHS Trust, b University of Cambridge, c University of Bristol,
d University of East Anglia, e Norfolk and Norwich University Hospitals NHS Trust, Norwich, f National Institute of Health Research (NIHR) Applied Research Collaboration (ARC) East of England (EoE)

Issue Date:	06.10.2020
Protocol Version No:	2.0
Protocol substantial amendment number:	02

Revision chronology:
Protocol v1.2 Original
Protocol v1.3 (amendment 1)
Protocol v2.0 (amendment 2)

Primary reason for Substantial Amendment 1: To allow participants to self-consent to the study during the Covid-19 pandemic. Increase in number of recruits permitted to be obtained from one site (Cambridge) as other sites paused non-Covid research. Additional changes: To allow review of scans when home working using a secure Information Technology (IT) environment

Substantial Amendment 2: Change to one of the primary outcomes related to the study retention rate at one year to take account of the relatively high mortality rate in this cohort. The original outcome stated that the study would be considered feasible if treated / not on treatment outcome was determined in ≥75% of the sample at 12 months from randomisation (S3.4.Phoenix_protocol v1.2_2019_07_01). This was amended to ≥75% of those **surviving** at 12 months post randomisation. This change was based on: i) a likely increased disease severity among patients who continued to have CT scans during the first wave of Covid-19 (many non-cancer scans were cancelled, skewing the study sample to those with cancer monitoring); and ii) findings from our own VORTEX Study (Vertebral fracture and Osteoporosis screening from Routine CT as an added EXtra – the VORTEX Study).

Corresponding author: Dr Ken Poole
Address: Dept of Medicine, University of Cambridge, Level 7, Hills Rd, Cambridge CB2 0QQ
Telephone: 01223 217580
Email: kp254@medschl.cam.ac.uk, kenneth.poole@nhs.net

For peer review only - http://bmjopen.bmj.com/site/about/guidelines.xhtml

KEY TRIAL CONTACTS	
Chief Investigator	Dr Ken Poole, 01223 217580, kp254@medschl.cam.ac.uk, kenneth.poole@nhs.net
Trial Co-ordinator	Karen Willoughby, 01223 217580, kw369@medschl.cam.ac.uk
Joint-sponsor(s)/co-sponsor(s)	Cambridge University Hospitals NHS Foundation Trust and the University of Cambridge
Funder(s)	NIHR RfPB stream
Statistician	Dr Stephen Kaptoge, 01223 748668 skk22@medshl.cam.ac.uk
Key Protocol Contributors	Dr Ken Poole<br>Consultant Rheumatologist<br>University of Cambridge<br>kp254@medschl.cam.ac.uk<br><br>Daniel Chappell<br>Research Analyst,<br>Cambridge University Hospitals NHS Trust<br><br>Dr Emma M Clark,<br>Reader in Rheumatology, University of Bristol<br>emma.clark@bristol.ac.uk<br><br>Dr Jane Fleming<br>Senior Research Associate<br>Department of Public Health and Primary Care<br>University of Cambridge School of Clinical Medicine<br>jane.fleming@phpc.cam.ac.uk<br><br>Dr Stephen Kaptoge,<br>Senior Statistician, University of Cambridge<br>skk22@medshl.cam.ac.uk<br><br>Professor Lee Shepstone<br>Professor of Medical Statistics,<br>University of East Anglia<br>l.shepstone@uea.ac.uk<br><br>Dr Tom Turmezei<br>Consultant Radiologist<br>CUHNFT<br>Tom@turmezei.com<br><br>Dr Adam P Wagner<br>Senior Research Fellow in Health Economics,<br>University of East Anglia<br>adam.wagner@uea.ac.uk<br><br>Ms Karen Willoughby,<br>Trial Manager, University of Cambridge<br>kw369@medschl.cam.ac.uk

For peer review only

For peer review only - http://bmjopen.bmj.com/site/about/guidelines.xhtml

Key words: Osteoporosis; osteoporotic fracture reduction; opportunistic screening; vertebral fracture.

ABSTRACT
Introduction: Two million computed tomography (CT) scans are performed each year in the UK (OECD data, 2014) incorporating the thoraco-lumbar region; up to a third reveal undiagnosed osteoporosis or vertebral fractures. PHOENIX-f aims to determine whether new technology can improve early detection of osteoporosis or vertebral fractures by repurposing existing CT scans and if this can be scaled up to a national clinical trial. Five hospital sites are participating (4 secondary and one tertiary level centre). CT Scans are shared using the Image Exchange Portal (IEP). Cambridge University Hospital will act as the reviewing hub

Methods & analysis:
Primary outcome measures:
- Recruitment rate. (375 recruits/ 10 months).
- Retention rate of those surviving at 12 months post randomisation.

Participants are recruited in CT departments whilst attending for scans and asked to complete a FRAX (Fracture Risk Assessment Tool) questionnaire following consent. FRAX uses a range of risk factors to predict the 10-year probability of a fracture occurring in the spine, hip, shoulder or wrist for people aged between 40 and 90 years. Above 90 years of age, the FRAX calculation becomes unreliable due to competing mortality risks.

Respondents whose calculated risk of major osteoporotic or hip fracture over the next 10 years is above the UK National intervention threshold are randomised into one of three parallel groups. Quantitative Computed Tomography (QCT, Mindways software) will be used in the reviewing hub to analyse the scans, determine bone density and identify prevalent vertebral fractures. Pathways and timescales for reporting results to GPs will depend on randomisation group.

Ethics and Dissemination: Approval granted 28.07.2019 by the East of England - Cambridge East Research Ethics Committee. **REC ref: 259482**

Dissemination will be through the Royal Osteoporosis Society. Publication strategy includes a full executive summary and plain English summary in peer reviewed journals.

Registration details

ISRCTN: 14722819
Trial Sponsor: Cambridge University NHS Foundation Trust & University of Cambridge
Sponsor's Reference: A095016

Strengths and limitations of this study:
- A pragmatic randomised controlled clinical study allowing flexibility to self-consent.
- Builds and expands on a pre-existing successful local service (CORTEX - Capturing Osteoporosis on Routine Tomography as an added EXtra) running at Cambridge University Hospitals (CUH).
- Willingness to consent and integrated technology (IEP) tested across multiple sites.
- Recruitment during Covid-19 will possibly be skewed towards the more seriously ill, as non-urgent and routine scans were cancelled. The retention rate outcome at one year post randomisation has been amended to take account of this.

Introduction: Older patients (65 years ≥in women, 70 years ≥in men) with undiagnosed spine fractures or low bone density attending hospital for CT scans stand to benefit from osteoporosis screening. Timely diagnosis and treatment of osteoporosis benefits many patient outcome domains (pain, quality of life, morbidity, and in the case of zoledronate treatment, mortality). An estimated 43% of over 60-year-olds undergoing CT scans (for abdominal and pelvic problems unrelated to their bones) were found to have osteoporosis or vertebral fractures when scan images were examined using computer-analysis methods[1]. However, radiologists reviewing CT scans reported only 16% of vertebral fractures in routine practice[2], and bone density is rarely calculated from CT scans. Systematic reviews indicate that missing these life-changing bone conditions can lead to serious, preventable pain, disability and health costs (>£2 billion per year from osteoporosis in the UK)[3]. Osteoporosis is a common disease in older women and men: osteoporosis is associated with >200,000 vertebral fractures and >85,000 hip fractures annually in the UK[4]. Osteoporosis-related fractures have a high morbidity and, despite low cost-effective treatments, women and men are increasingly presenting at clinics with advanced multiple vertebral fractures, loss of body height, compressed abdominal contents, dysphagia,

For peer review only - http://bmjopen.bmj.com/site/about/guidelines.xhtml

severe and chronic pain and reduced mobility. Despite an ageing UK population, osteoporosis remains under-diagnosed and under-treated[5].

Rationale:
Osteoporosis case finding rate in primary care is low and there is no national mandate for screening. High risk patients may be referred for a dual-energy x-ray absorptiometry (DXA) scan and are offered treatment if bone mineral density (BMD) is low and/or fracture risk sufficiently high. Vertebral fractures are infrequently detected in usual care even though they (alongside hip fractures) cause the biggest burden to patients and the health service. We hypothesise that targeted screening will improve upon usual care and improve diagnosis.

New software has enabled more accurate diagnosis of osteoporosis and sub-clinical vertebral fractures from routine CT scans. The PHOENIX pathway is a simple and widely practicable intervention. It has been developed to apply these new software programmes to CT scan images and reuse them for measuring bone density, fracture risk and to identify crushed vertebrae[6,7]. It couples timely diagnosis with a report to GPs containing advice on NICE-approved, cost-effective treatments and lifestyle advice to reduce osteoporotic fractures[8]. We expect benefits to accrue through early diagnosis and treatment of osteoporosis. Preventing fractures could lead to decreased societal and social burden, and reduced health- and social-care costs.

Reasons for opportunistic osteoporosis screening during a CT scan:

- CT attenders are a high-risk population: >30% of older adults have undiagnosed vertebral fractures or osteoporosis that can be effectively treated once identified[9]
- The PHOENIX pathway diagnoses osteoporosis on scans already undertaken, so there is no requirement for additional x-rays or hospital attendance
- Screening is effective at reducing hip fractures. A recent trial of primary screening based on clinical risk factors and targeted bone density scanning reduced the hip fracture incidence by 28% over five years, with a number needed to screen (NNS) of 111 to prevent one hip fracture[10]
- Systematic reviews of screening for osteoporosis suggest a number needed to screen (NNS) of 43 people (aged 75 or over) to prevent one vertebral fracture[11]
- Fracture risk is currently assessed opportunistically by GPs. Current advances in IT software could augment this practice to make more efficient and effective use of healthcare resources, as recommended by the National Institute for Health and Care Excellence (NICE), Clinical Guideline (CG) 146[11]
- A 2013 study found that radiologists failed to report 84% of clinically important vertebral fractures from routine CT scans[4]. However, once combined with new computerised analysis methods, CT scanners can effectively determine bone density and fractures [7]
- There is a fourfold risk of another vertebral fracture occurring after the first one[12], which may indicate that osteoporosis is a quickly progressing disease once a first fracture occurs
- Generalisability: one million CT scans were performed in the UK in 1996, increasing to five million in 2016[13], permitting screening of many 'at-risk' patients. Pathways such as PHOENIX are not complicated, nor technically demanding for hospitals routinely operating DXA scans

Assessment and management of risk:
There is no clinical risk in this trial to the participants as there is no clinical intervention. We ask participants to answer two short questionnaires, one shortly after randomisation, and another at the one-year follow-up. A small fraction of participants will also be invited to participate in telephone interviews to discuss their study experiences.

Methods and Analysis:
PHOENIX-f has been designed as a feasibility study to determine if:
i) the PHOENIX pathway/intervention is feasible and acceptable to patients.
ii) a definitive trial to evaluate PHOENIX nationally is feasible.

PHOENIX-f is a parallel three arm, non-blinded randomised controlled study. We have designed three arms to allow comparison between (1) the PHOENIX pathway, (2) an alternative active control 'middle' group (to assess whether GPs will treat patients based solely on FRAX scores posted out to them, but with no further treatment recommendations), and (3) a control ('usual care') group. Potential participants are identified ahead of attendance by the research team reviewing CT lists. Those eligible by age and region of the body being scanned are handed the PHOENIX-f documentation whilst attending the CT department. An Informed Consent Form (ICF; Supplemental material file 1) first guides the potential participant through several eligibility screening questions:

For peer review only - http://bmjopen.bmj.com/site/about/guidelines.xhtml

i. *Do you take medication for your bones once a week that requires you to sit or stand upright?*
ii. *Do you have a yearly infusion (drip) or injection for your bones?*
iii. *Do you have a hip replacement? Have you ever had a hip operation?*

If there is an affirmative answer to any of these questions, individuals are asked to return their forms. Those individuals who answer 'no' to all the questions, are asked to supply key demographic data: Date of birth, height, weight, country of origin and ethnicity (needed for accurate FRAX calculation) and NHS number (receptionists support the patient as needed with the latter). Individuals are also asked whether they know the reason for their scan.

On the ICF's last page, the eight validated bone health-relevant questions from the FRAX questionnaire should be completed, along with patient address, telephone number, GP name and GP address. Completed paperwork is either collected or scanned and sent to a secure NHS.net email account. FRAX answers are reviewed by the research team at Cambridge who use the results to calculate the 10-year fracture risk score without femoral neck BMD (figure 1, left panel). Those at low risk of fracture (green category according to the National Osteoporosis Guideline Group guidelines, NOGG) will be excluded from the study. Those in the remaining 'red' and 'amber' risk categories will be randomised to one of 3 arms:

1 PHOENIX pathway (the new intervention)
2 ACTIVE CONTROL (FRAX – GP pathway)
3 USUAL CARE.

Bone mineral density (BMD) in the left total hip region, left femoral neck and/or lumbar spine (L1-L3) is measured in all participants (those not randomised to Group 1 will be measured later at one year) using the CT images already captured and computer-aided diagnosis of vertebral fractures is performed. Each individual's FRAX fracture risk estimate is then recalculated taking account of their femoral neck BMD value and revised prior fracture status if spine imaging revealed any vertebral fracture. The NOGG guidance tool within FRAX is followed to determine whether treatment is recommended, provided no contra-indications exist (Figure 1, middle and right panels).

The PHOENIX pathway (Group 1 only) consists of a clinician verified report posted to the GP with details of femoral neck and/or spine bone density results, vertebral fracture diagnosis (yes/no/ level, if any), FRAX estimated 10-year risk of major osteoporotic fracture, any necessary follow-up investigations and a simple treat/do not treat decision based on guidelines, with recommended clinically effective and cost-effective drug suggestions. All treatment recommendation phrases are derived from a continually updated algorithm and stored within the QCT Pro reporting engine. The clinical decision tree that guides the investigator to the recommendations can be found in Supplemental material file 2. In Group 2 only the completed FRAX questionnaire is sent to the GP, who can calculate the risk themselves by entering responses onto the online calculator (https://www.sheffield.ac.uk/FRAX/tool.aspx?country=9) . Responsibility for subsequent investigations including referrals for DXA and/or treatment, will remain with the GP. In Group 3, the GP receives a letter informing them of their patient's involvement in the study, but no further reports or information at this point. For all participants, the full FRAX fracture risk and Quantitative computed tomography bone mineral density (QCT BMD results) based on the previous scan will be calculated and sent to the GP one year after randomisation to enable them to make informed decisions regarding possible treatment

Baseline and one-year follow-up postal questionnaires sent to all randomised participants will collect data on quality of life and resource use in relation to bone health for health economics analysis. For non-responders a telephone interview will be attempted aiming to collect the questionnaire data. Patient and clinical perspectives of study involvement will also be explored through semi-structured interviews by telephone.

Data will be held and processed in accordance with the Data Protection Act 2018. All study data will be held securely. It will not be disclosed to third parties. All staff working on the study owe a duty of confidentiality to the participants. Manual records will be held securely (for example in locked filing cabinets). Electronic records will be held on a secure network requiring user ID and password access. Individuals will not be identifiable from the results of the trial.

Trial Management. The Project Management Team is responsible for the day to day running of the study to discuss practical aspects and ensure it is progressing. The group meets to address various specialist aspects of the study. The Trial Management Group (TMG) comprises the protocol authors and one PPI member. They are responsible for the overall conduct of the study and ensuring NIHR milestones are achieved. Prior to Covid-19 separate PPI meetings were held which enabled those attending to influence the study design.

For peer review only - http://bmjopen.bmj.com/site/about/guidelines.xhtml

Study Flow Chart

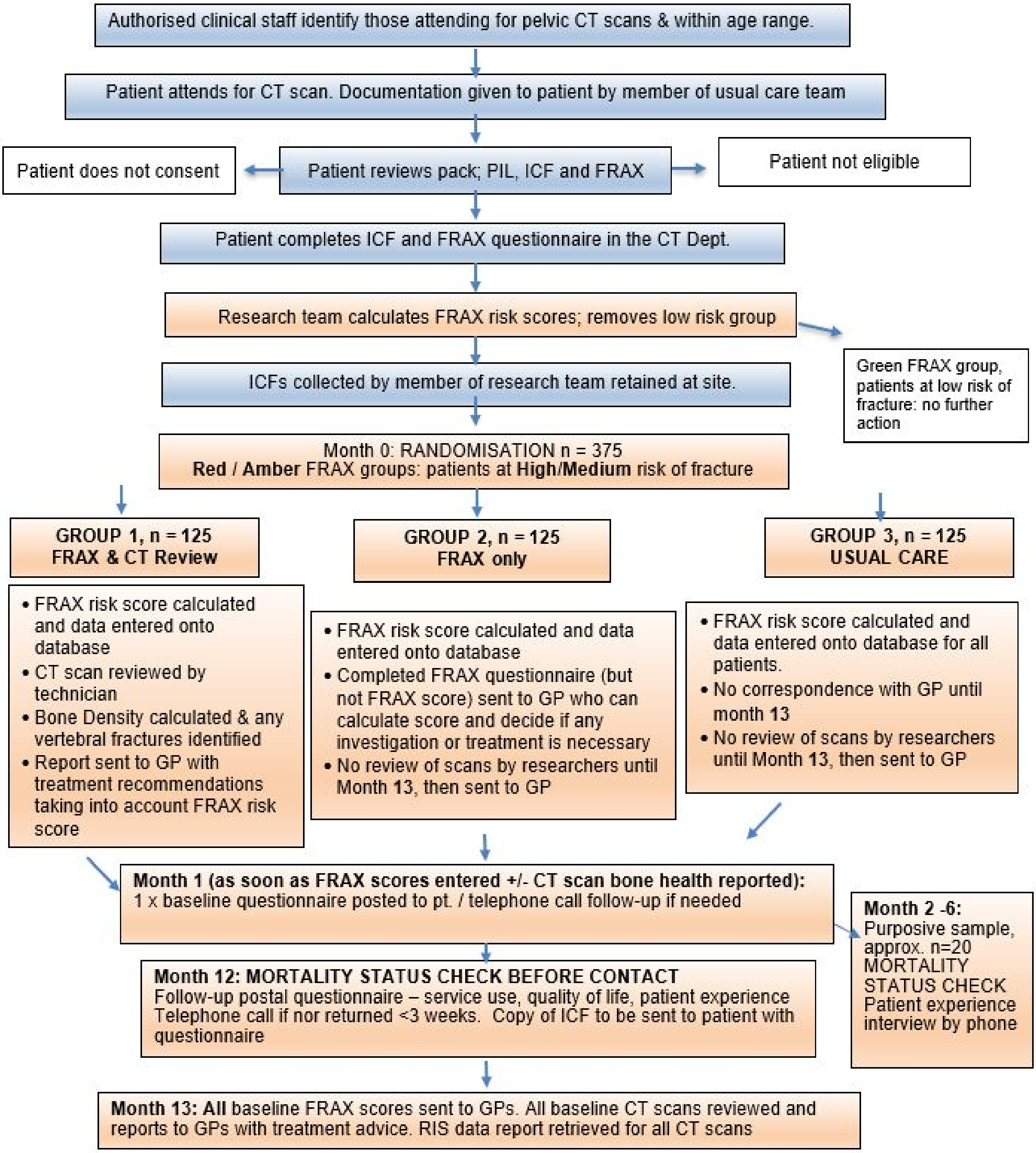

*Radiology Information System

For peer review only - http://bmjopen.bmj.com/site/about/guidelines.xhtml

Relevant concomitant care and interventions prohibited during the trial:

- Participants receiving anti-resorptive medication or who currently have hip metalwork at the time of recruitment will fail screening for the trial, but there are no prohibited interventions during the trial.

Outcome measures/endpoints:

- Rate of recruitment measured over 10 months
- Retention rate of those surviving at 12 months post randomisation

Exploratory endpoints/outcomes:

- Feasibility of using the Image Exchange Portal (IEP) in a 'hub and spoke' model for the Phoenix Study
- Treatment and survival rate at 12 months across 3 arms
- Acceptability to patients of the PHOENIX intervention

Inclusion criteria:

Participants eligible for the trial should fulfil all of the following at randomisation:

- Attendance at one of five participating radiology departments
- Able to provide valid informed consent
- Aged ≥65-90 (women) or ≥75-90 (men) attending for CT for any clinical reason, where the spine and/or hips are visible in scan images

Exclusion criteria:

- Aged > 90
- Metalwork in hips
- Unable to provide valid informed consent
- Known to be receiving prescription treatment for osteoporosis other than calcium/vitamin D (i.e. bisphosphonate drug, strontium ranelate, denosumab, raloxifene or teriparatide)
- Prone CT scan

Patient and Public involvement (PPI)*:*

PPI representatives actively participated in the study design in focus groups with the project management team and assisted in the design of data collection tools, including the Informed Consent Form and Patient Information Leaflet. They provided guidance on the acceptability of approaching participants in CT scan areas and insight into the anxiety of attending hospital amongst some more elderly members of society.

Sample size calculations

The primary outcomes of the feasibility study are participant recruitment rate (willingness to participate) and retention rate of surviving patients at 12 months. We intend to evaluate the technical procedures which would be used in a definitive Health Technology Assessment (HTA) trial with fracture incidence (rather than recruitment/retention) as the primary outcome. To help set the target recruitment numbers for the feasibility study, we have used the surrogate outcome of osteoporosis treatment rates at 12 months follow up to permit sufficient power to assess and conduct cross-group comparisons across trial arms within the period of the feasibility study.

We have also contemplated the scaling up from Phoenix-f, by considering scenarios based on previous data [1, 2, 14]. Only those participants in the red/amber FRAX group **(Figure 1)** will be randomised, so the number of invitations needed to reach any recruitment target will also depend on the prevalence of the red/amber FRAX group. In our local CORTEX service the prevalence of red/amber FRAX varied between 30% at age 40 and 50% at age 95 **(Figure 2),** with overall prevalence 40.5% (95% CI: 35.7%, 45.5%). There is no published UK data to compare with this but comparable prevalence was reported In a single study in the USA investigating combined bone density and fracture detection in routine CT investigations, where 42.3% (of 571 consecutive patients) were newly detected as having osteoporosis[1]. A conservative assumption from preliminary work is that PHOENIX-f will identify a further 9.6% of screened patients for treatment and that GPs will take action to treat at least half of these, resulting in an expected 4.8% minimum detectable difference. If, in Phoenix-f, the rate of treatment at 12 months in Group 2 (the FRAX-GP arm) matches that observed in the SCOOP study[6] (15.5%), we would consider that an improvement in treatment rate of 4.8% would be clinically significant.

For peer review only - http://bmjopen.bmj.com/site/about/guidelines.xhtml

As only participants in the ‘red’ and ’amber’ FRAX group will be randomised **(Figure 1)**, invitation numbers required to reach the recruitment target will depend on the prevalence of these FRAX groups. In the CORTEX study, the prevalence of red/amber FRAX varied between 30% (at age 40) to 50% (at age 95) - **(Figure 2),** with overall prevalence being 40.5% (95% CI: 35.7%, 45.5%).

Figure 1. FRAX UK age-specific fracture risk assessment and intervention thresholds.

Figure 2. Age-specific prevalence of red/amber FRAX risk category in CORTEX study data.

Power for treatment proportions

Assuming that osteoporosis treatment percentages at 1-year follow up in the USUAL-CARE vs. FRAX-GP vs. PHOENIX-F would be 4.5% vs. 15.5% vs. 20.3% respectively (i.e. as observed in SCOOP trial vs. with expected improvement in PHOENIX-F), a total of 375 participants recruited and randomised in the ratio 1:1:1 will have 96% power (see Figure 3) to detect linear trend in treatment proportions based on a two-sided test of binomial proportions at 5% statistical significance level**.** The same sample size will have 93% power for a secondary two-degree of freedom test of between group contrasts in comparison with USUAL-CARE. When allowing for up to 25% attrition due to non-differential dropout or incompleteness of outcomes, the remaining sample size of 280 participants will have >84% power to detect the same magnitude of differences (Figure 3).

Figure 3. Power vs sample size to detect differences in 12-month treatment rates in a 3-arm trial.*

* Assuming treatment percentages of 4.5% vs. 15.5% vs. 20.3% with 1:1:1 randomisation to USUAL-CARE vs. FRAX-GP vs. PHOENIX-F and respectively, and type 1 error = 0.05.

Figure 4 shows the estimated total number of invitees needed to recruit the 375 participants with a FRAX 10-year risk in the red/amber zone, assuming different response rates and different prevalence estimates. We expect the scenario of 30% response rate (similar to SCOOP) combined with 40% prevalence of red/amber FRAX most likely. Thus, it would be necessary to invite a total of 3,125 participants (or 625 invites per centre) to achieve the recruitment target of 375 randomised participants (75 per centre).

Figure 4. Estimated total invites needed to recruit 375 red/amber participants, assuming different response rates and different prevalence estimates.

Figure 5 shows the expected eventual number of participants available for analyses by FRAX category (red, amber and green FRAX) when aiming to recruit and randomise 375 red/amber FRAX participants, with green FRAX only included afterwards for secondary comparison.

Figure 5. Eventual number of participants available for analyses (red, amber and green FRAX) when aiming to recruit and randomise 375 red/amber FRAX participants, with green FRAX only included afterwards for secondary comparison.

Power for recruitment and retention

Hypothesising that the recruitment rate (i.e. consent, FRAX completion, eligibility, and CT imaging) would be similar to that observed in the SCOOP primary prevention trial (p_0 = 30%); a feasibility study aiming to randomise 375 of 938 (40%) consenting participants (i.e. 30% of 3125 total invited) would estimate the trial’s response rate with a precision of 1.6% (i.e. half-width of the 95% confidence interval) – (**Figure 6**). The randomised sample of 375 patients will provide 80% power to detect a difference of at least 6% or larger in the 12-month overall trial retention rates from an hypothesised 75% retention rate (as with the SCOOP trial) based on two-sided one-sample test of proportion at 5% significance level (i.e. providing statistical evidence that the overall retention rate may be as low or high as 69% or 81% respectively, as opposed to 75%) – **(Figure 7).** In addition, against the same expectation, the 1:1:1 randomised comparison (i.e. PHOENIX vs. FRAX to GP vs. USUAL-CARE pathways) will have >80% power to detect at least 12.2% absolute difference in retention rates between trial arms (or RR = 1.16) based on two-sided two-sample test of proportion at 5% significance level.

Figure 6. Sample size for 80% power to reject null hypothesis of 30% response rate.

Figure 7. Sample size for 80% power to reject null hypothesis of 75% retention rate.

For peer review only - http://bmjopen.bmj.com/site/about/guidelines.xhtml

Randomisation
Block randomisation stratified by centre, sex and age group (Female: <75 vs ≥75, Male: <80 vs ≥80) will be utilised. Randomisation will be performed using the web application Research Electronic Data Capture (REDCap) *https://www.project-redcap.org/.*

Statistical analysis
Daily screening logs will be kept determining: i) the total number of days (n) where patients were approached in each centre; and ii) the daily totals of PHOENIX-f invitation packs distributed. This log will also record if a numbered pack was not given out on a day (e.g. if the patient did not arrive for scanning, or the receptionists omitted to give out the pack). The total number of consented individuals (x) as well as the number of consented individuals per day (total x/n overall, and per centre) will be calculated. The number of consented but subsequently excluded individuals (y) will be kept (e.g. for subsequently discovered metalwork in the scan, technical issues with scan). The number of randomised participants will be calculated (z= x-y), and the rate of randomisation (z/n overall, and per centre). These data will be captured from screening logs and entered manually onto a database.

The Research Electronic Data Capture (REDCap) database will be used to record details of all consenting patients (z). The database will be locked on completion of data entry and when missing data or any discrepancies are resolved. The data will be exported for statistical analysis to the study statisticians with assistance from the Chief Investigator.

Predefined Statistical Analysis Plan: Response rates will be calculated as the proportion of participants invited who (a) consent to take part, (b) meet eligibility criteria, and (c) are randomised, with 95% confidence intervals to assess the extent to which the realised response rate deviates from the expected as detailed in the power calculations above. Cumulative participant accrual over time will be plotted overall and by recruitment centre to assess any major differences in accrual and assess progress toward target. Logistic regression will be used to compare randomised groups with respect to the 12-month outcomes of treatment rates and participant retention. Baseline stratification covariates (i.e. centre, sex, and age) will be adjusted for in the models. Analyses will be on an intention-to-treat basis.

Recruitment had to be paused due to the covid-19 pandemic, then restarted in just one centre without face-to-face contact with patients. We will be able to compare recruitment rates during these two phases of the study: success of recruitment by clinical approach and discussion as in the trial's initial phase versus self-consent on the basis of just handing out recruitment packs after covid-19 restrictions were brought in.

Qualitative analysis
Both the baseline and one year follow up questionnaires to be sent to all patients will include space for participants to feedback their experience of the study. First explaining how this will help, this section includes example open questions such as "Do you have any comments on being offered a bone health check at the same time as your CT scan? Or on taking part in this research study? / how you were asked to join? / your decision to take part?".

Semi-structured interviews will be used with a smaller sub-sample of study participants (n=20) to allow for more in-depth exploration of issues that participants may raise in written feedback or during interview. Recordings will be transcribed by an academic transcription service following a confidentiality contract and anonymised by a member of the research team changing names of people and places and any identifiable details

Key research questions for the PHOENIX-F process evaluation are:

- What are the implementation facilitators and barriers for the PHOENIX protocol?
- How acceptable to the intended patient group is the process of PHOENIX screening?
- How do implementation and acceptability issues affect uptake of the CT bone health screening and any subsequent GP investigation and recommended bone protective treatment?

A combination of interviews and focus groups with staff groups who will be key to the effective delivery of PHOENIX screening will also be undertaken, both in hospital and primary care, in order to understand their different perspectives. These staff groups are research nurses, receptionists and radiographers in CT departments and GPs.

Analyses will be descriptive. The software programme NVivo v12 (QSR) will be used to manage and index the qualitative data (anonymised transcripts of audio-recordings from interviews and focus groups) before charting, mapping and interpretation taking a framework approach[15] [Ritchie & Spencer] to thematic analysis, an approach suitable for qualitative research in which data collection is relatively focussed on key questions, as in this feasibility study which will draw on recognised process evaluation approaches[16,17].

For peer review only - http://bmjopen.bmj.com/site/about/guidelines.xhtml

Economic analysis
Completion rates on the quality of life measure (EQ-5D-5L) and resource use items included within the postal survey will inform whether an economic evaluation is feasible using these data collection approaches in the definitive trial. Patterns of response and non-response will be explored to see if they suggest ways the postal survey can be refined to improve data completeness.

Funding Statement: The study was supported by the NIHR Research for Patient Benefit stream. Funding is in place to 31.03.2022. Grant number PB-PG-0816-20027

Competing interests statement: No competing interests noted

Word Count: 4000 (excludes abstract, figures and references)

Contributorship statement:
Kenneth Poole is the chief investigator for the study and is responsible for the concept and design of the overall study and manuscript final approval
Daniel Chappell: data quality assessment, data acquisition, data extraction, data analysis, interpretation of data
Emma M Clark: methods design and providing feedback and approval on manuscript, manuscript final approval
Jane Fleming: interpretation of data, providing detailed feedback on manuscript, manuscript final approval
Dr Stephen Kaptoge: concept and design of overall study, data extraction, data analysis, interpretation of data, manuscript final approval
Lee Shepstone: methods design and manuscript final approval
Tom Turmezei methods design, search strategy design, quality assessment, data analysis, interpretation of data, and approval of final manuscript
Adam P Wagner: interpretation of data, providing feedback and detailed contributions on manuscript, manuscript final approval
Karen Willoughby: design and writing of the manuscript

Authorship:
All authors agree to be accountable for all aspects of the work to enable questions relating to the accuracy or integrity of any part of the work to be appropriately investigated and resolved

Data availability statement:
Researchers involved in the trial who will have access to the full dataset. Applications for access to the final trial dataset will be through the Trial Management Group. All members of the TMG will have full access to the dataset, and a controlled access model (openly available to all applicants) will be followed as set out in the MRC and NIHR guidance
https://www.methodologyhubs.mrc.ac.uk/files/7114/3682/3831/Datasharingguidance2015.pdf

References

[1] Lee SJ, Binkley N, Lubner MG, et al. Opportunistic screening for osteoporosis using the sagittal reconstruction from routine abdominal CT for combined assessment of vertebral fractures and density. Osteoporosis International 2016;27(3):113, 1-36. doi: 10.1007/s00198-0153318-

[2]. Carberry GA, Pooler BD, Binkley N, et al. Unreported Vertebral Body Compression Fractures at Abdominal Multidetector CT. Radiology 2013;268(1):120-26. doi:10.1148/radiol.13121632

[3]. Al-Sari UA, Tobias J, Clark E. Health-related quality of life in older people with osteoporotic vertebral fractures: a systematic review and meta-analysis. Osteoporosis International 2016;27(10):2891-900. doi: 10.1007/s00198-016-3648-x

[4] Department of Health. Hospital episode statistics (England) 2006

[5] Jewell PD, Mitchell R, Javaid MK, et al. Reporting and Follow-up of Vertebral Fractures Seen on Imaging; Can Radiologists Help Reduce the Number of Hip Fractures? Osteoporosis International 2014;25:S674-S74.

For peer review only - http://bmjopen.bmj.com/site/about/guidelines.xhtml

[6] Harvey N, Cushnaghan J, Lawrence W, et al. Anxiety, Fracture Risk and Adherence to Treatment: The MRC SCOOP Trial. Osteoporosis International 2012;2 3:S531-S31.

[7] Steiger P, Staal J, Engelke K, et al. Validation of an Automatic Vertebral Prevalent Fracture Classifier Based Upon Full Vertebral Shape. Osteoporosis International 2013;24:S307-S08.

[8] Shepstone L, Lenaghan E, Cooper C, et al. Screening in the community to reduce fractures in older women (SCOOP) - a randomised controlled trial Lancet 2017(pii)S0140-6736(17)32640-5

[9] OECD (2014). Computed tomography (CT) exams, total. Health: Key Tables from OECD, OECD - ISSN 2075-8480

[10] National Institute for Health and Care Excellence. Osteoporosis: assessing the risk of fragility fracture 2012. NICE guidelines CG146, 2015

[11] Nelson HD, Haney EM, Dana T, et al. Screening for Osteoporosis: An Update for the US Preventive Services Task Force. Annals of internal medicine 2010;153(2):99-W43. doi:10.7326/0003-4819-153-2-201007200-00262

[12] Lindsay R et. Al. Risk of new vertebral fracture in the year following a fracture. JAMA. 2001 Jan 17;285(3):320-3

[13] https://www.england.nhs.uk/statistics/wp-content/uploads/sites/2/2016/08/Provisional-Monthly-Diagnostic-Imaging-Dataset-Statistics-2017-05-18.pdf

[14].Pickhardt PJ, Pooler BD, Lauder T, et al. Opportunistic screening for osteoporosis using abdominal computed tomography scans obtained for other indications. Annals of internal medicine 2013;158(8):588-95. doi: 10.7326/0003-4819-158-8-201304160-00003

[15] Ritchie J, Spencer L. Qualitative data analysis for applied policy research. In: Bryman A, Burgess R, editors. Analyzing qualitative data. London: Routledge; 1994. p. 173–94.]

[16] Moore G, Audrey S, Barker M, Bond L, Bonell C, Hardeman W, Moore L, O'Cathain A, Tinati T, Wight D, Baird J. Process evaluation of complex interventions: Medical Research Council guidance. MRC Population Health Science Research Network, London, 2014

[17] Graham F Moore, Suzanne Audrey, Mary Barker, Lyndal Bond, Chris Bonell, Wendy Hardeman, Laurence Moore, Alicia O'Cathain, Tannaze Tinati, Daniel Wight, Janis Baird. Process evaluation of complex interventions: Medical Research Council guidance. BMJ 2015;350:h1258 doi:10.1136/bmj.h1258

Supplemental material file 1: Consent Form and FRAX questionnaire

Supplemental material file 2: Clinical Decision Tree

Figure 1. FRAX UK age-specific fracture risk assessment and intervention thresholds.

For peer review only - http://bmjopen.bmj.com/site/about/guidelines.xhtml

Figure 2. Age-specific prevalence of red/amber FRAX risk category in CORTEX study data.

Figure 3. Power vs sample size to detect differences in 12-month treatment rates in a 3-arm trial.*

* Assuming treatment percentages of 4.5% vs. 15.5% vs. 20.3% with 1:1:1 randomisation to USUAL-CARE vs. FRAX-GP vs. PHOENIX-F and respectively, and type 1 error = 0.05.

Figure 4. Estimated total invites needed to recruit 375 red/amber participants, assuming different response rates and different prevalence estimates.

Figure 5. Eventual number of participants available for analyses (red, amber and green FRAX) when aiming to recruit and randomise 375 red/amber FRAX participants, with green FRAX only included afterwards for secondary comparison.

Figure 6. Sample size for 80% power to reject null hypothesis of 30% response rate.

Figure 7. Sample size for 80% power to reject null hypothesis of 75% retention rate.

Supplemental material file 1: Consent Form and FRAX questionnaire

Supplemental material file 2: Clinical Decision Tree

For peer review only

For peer review only - http://bmjopen.bmj.com/site/about/guidelines.xhtml

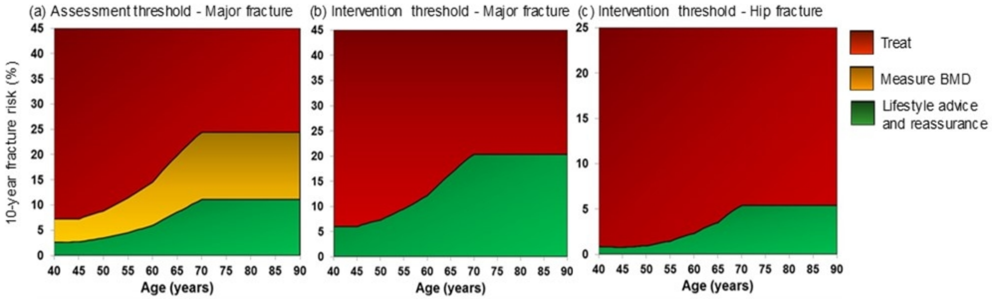

Figure 1. FRAX UK age-specific fracture risk assessment and intervention thresholds.

170x51mm (150 x 150 DPI)

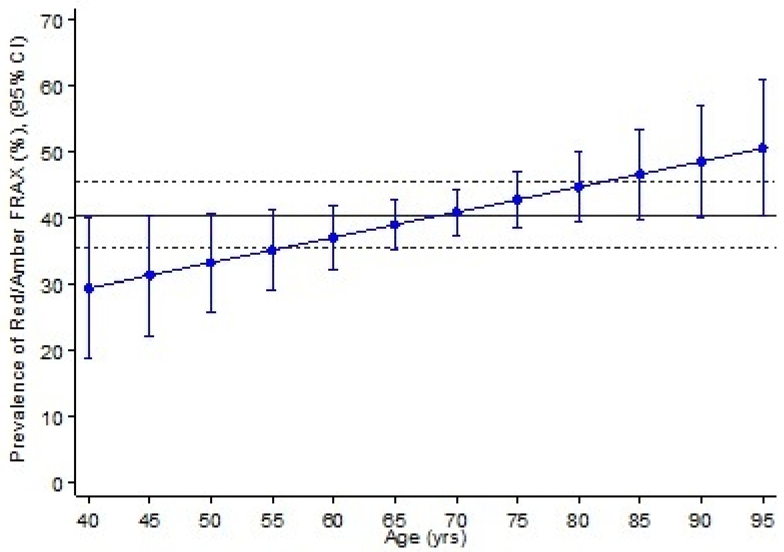

Figure 2. Age-specific prevalence of red/amber FRAX risk category in CORTEX study data

109x79mm (96 x 96 DPI)

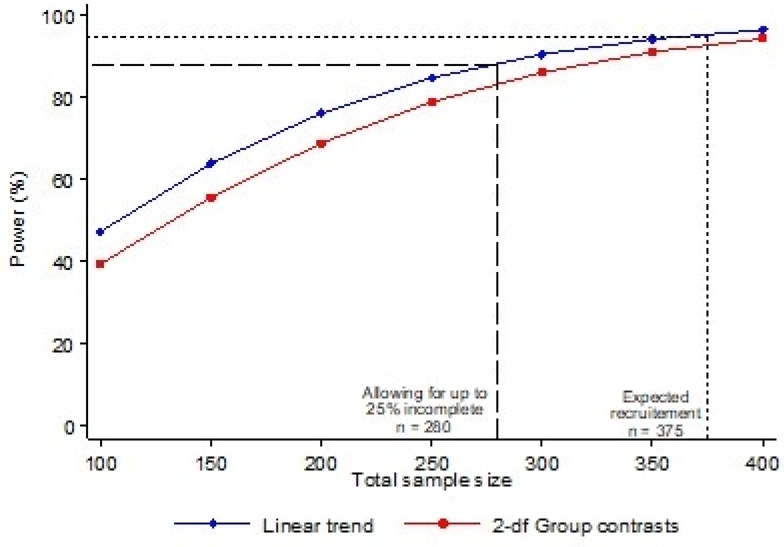

Figure 3. Power vs sample size to detect differences in 12-month treatment rates in a 3-arm trial.*

109x79mm (96 x 96 DPI)

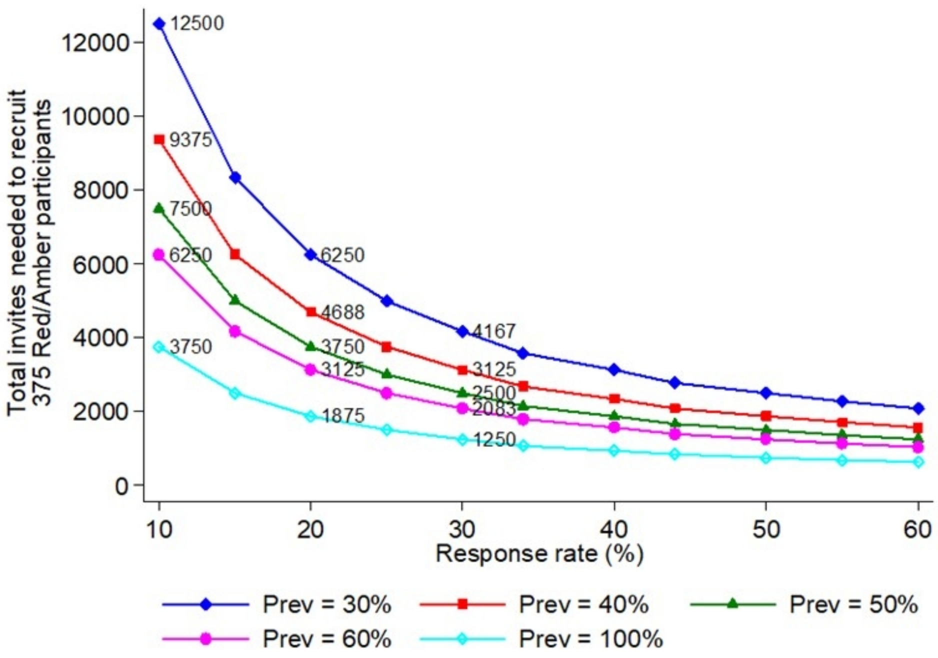

Figure 4. Estimated total invites needed to recruit 375 red/amber participants, assuming different response rates and different prevalence estimates.

136x99mm (150 x 150 DPI)

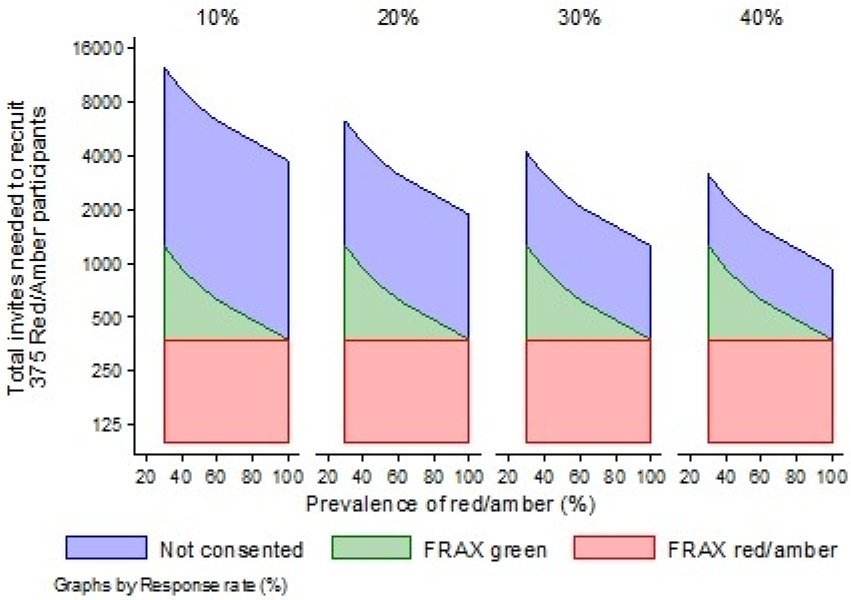

Figure 5 shows the expected eventual number of participants available for analyses by FRAX category (red, amber and green FRAX) when aiming to recruit and randomise 375 red/amber FRAX participants, with green FRAX only included afterwards for secondary comparison.

120x87mm (96 x 96 DPI)

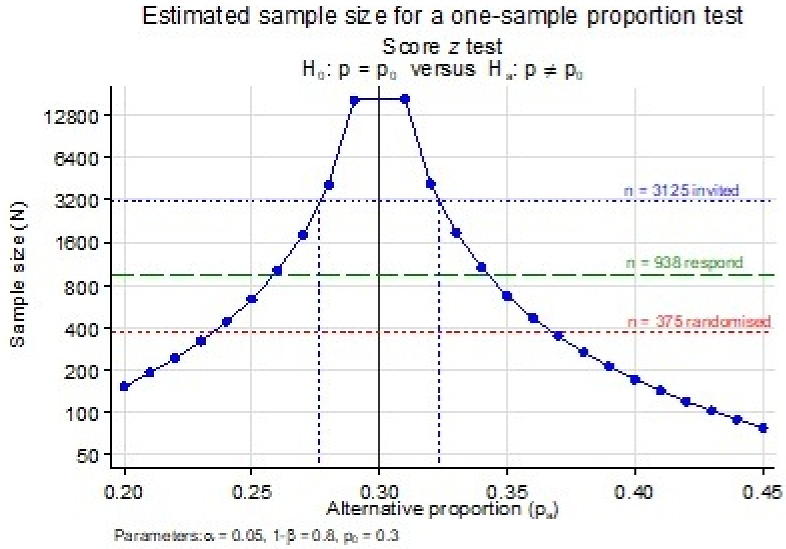

Figure 6. Sample size for 80% power to reject null hypothesis of 30% response rate.

109x79mm (96 x 96 DPI)

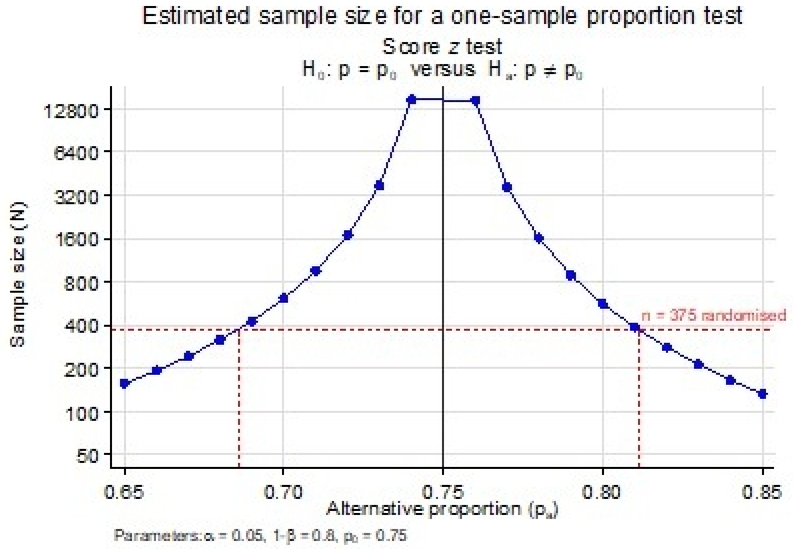

Figure 7. Sample size for 80% power to reject null hypothesis of 75% retention rate.

109x79mm (96 x 96 DPI)

Appendix 1

PHOENIX

Picking up Hidden Osteoporosis Effectively during Normal CT Imaging without additional X-rays

For peer review only

Osteoporosis is a condition that makes bones become fragile and break more easily, especially in the spine and hips. Osteoporosis can be treated. We would like to diagnose it earlier and are running a study called PHOENIX-f to see if this can be done using the scan that you are having today. There are no extra scans, other tests or treatment required to join the study.

If you are interested in being part of the study, please turn over the page and read on

If you do not wish to take part in the study, can you please tell us why in the box below? This would be really useful for us to know, but you do not have to tell us.

Now please return this form to the PHOENIX-f returns box

Thank you for your co-operation

For peer review only - http://bmjopen.bmj.com/site/about/guidelines.xhtml

By completing this form, we understand that you are happy for one of our research team to talk to you about the study.

Your scan taken today can also be used to measure your bone health.

We can't use every scan, so please answer the following 3 questions to see if we can use yours.

Please circle your answer

**Do you take medication for your bones once a week that require you to sit or stand upright?**	**Y**	**N**
**Do you have a yearly infusion (drip) or injection for your bones?**	**Y**	**N**
**Do you have a hip replacement? Have you ever had a hip operation?**	**Y**	**N**

If you answered 'Yes' to any of the questions above unfortunately we will be unable to use your CT scan today for PHOENIX-f.

Please return this form to the PHOENIX-f returns box.
Thank you for your co-operation.

If you have answered 'NO' to ALL the questions above we will be able to review your CT scan for PHOENIX-f.

In order to understand the study more, please read the patient information leaflet. Then, if you are interested in taking part in PHOENIX to measure your bone health, please fill in the questionnaire overleaf and complete the consent form

For peer review only

For peer review only - http://bmjopen.bmj.com/site/about/guidelines.xhtml

PHOENIX-f Consent Form

Chief Investigator: Dr Ken Poole, Consultant in Rheumatology and Metabolic Bone Disease, Cambridge

Please *initial* boxes below, then sign and date the form and add your address ***Initials***

1. I confirm that I have read and understood the patient information for the study. ☐
2. I understand that my participation is voluntary and that I am free to withdraw at any time without giving any reason, without my medical care or legal rights being affected. ☐
3. If I decide to withdraw from the trial I agree to data already collected to be used for Phoenix-f. ☐
4. I give permission for the study researchers to have access to my scan and relevant NHS databases in order to conduct this research ☐
5. I agree for my personal details and relevant medical information to be sent from my hospital to the study team at Addenbrooke's Hospital for the purpose of the research. I understand that scans have identifiable data and will be kept securely at Addenbrooke's Hospital and will only be accessed by the research team ☐
6. I agree to the research team contacting my GP to notify them of my participation in the study and any results ☐
7. I give permission to be contacted by post or phone by the study team at Addenbrooke's Hospital. I understand that this will be to send me 2 questionnaires and may include an invitation to talk to a researcher about my experience of the study ☐
8. I agree to take part in the above study. ☐

Mr / Mrs / Ms / Dr (please circle as appropriate)

....

Please enter first and family name above

………………………………………………………. *Date:* ………………………

Your Signature

Date of Birth: .... *Height:* .... *Weight:* ....

NHS number: ....

Why are you having a scan today? ....

The following questions are required for accurate bone health calculation:

Your country of origin: .... *Ethnicity:* ....

PLEASE TURN OVER PAGE TO COMPLETE THIS FORM

For peer review only - http://bmjopen.bmj.com/site/about/guidelines.xhtml

4

BONE HEALTH QUESTIONNAIRE	Yes	No	Not Sure
Have you broken any bones since you turned 40?			
If Yes, which bones?			
Did your mother or father ever break a hip?			
Do you currently smoke?			
Do you take steroid tablets now (eg: 'prednisolone'), or have you taken steroid tablets in the past, for longer than three months at any time?			
Have you been diagnosed with rheumatoid arthritis?			
Have you been diagnosed with any of the following?<br>**(Tick 'yes' if any of the following apply)**<br>• Premature menopause (before 45yrs of age)<br>• Insulin dependent diabetes<br>• Brittle bone disease, Ankylosing spondylitis<br>• Overactive thyroid<br>• Low testosterone level (men only)<br>• Chronic malnutrition or malabsorption eg: anorexia or coeliac disease<br>• Chronic liver disease			
Do you drink three or more units of alcohol a **day**, on average? (That's 1 1/2 pints of beer or 3 glasses of wine or 3 short measures of spirits)			

Your Address (for questionnaires):

....

....

....

....

Post Code: .... **Your Telephone no:** ....

Your GP Name and address:

....

....

Now please return this form to the PHOENIX-f returns box. Thank you for your co-operation

PHOENIX Methodology paper_2021_04_29_ submission 4

For peer review only

PHOENIX-f Clinical Decision Tree *part 1*

Initial steps

1. Retrieve and visually inspect CT images for any vertebral fractures with AP and lateral localiser views (using Mindways QCT PRO SlicePick for 6-point morphometry).

2. Measure CTXA Hip and/or Spine BMD with QCT PRO, noting Femoral neck & Hip BMD results (in g/cm2) and/or Average L1-L3 Spine BMD results (in mg/cm3).

3. Recalculate FRAX online using the Mindways Femoral neck/Hip BMD (g/cm2) value & change 'Previous Fracture' to 'Yes' if a Grade 2 or 3 fracture is found at step 1.

4. If only one (Hip or Spine) result is available, follow the GP Advice Clinical Decision Tree here to generate automatic GP Advice text.

5. If both Hip BMD and Spine BMD results are available, CTXA Femoral neck/Hip results usually dominate, and the following generic Spine 3D QCT GP advice box text is automatically added, "See CTXA Hip Bone Mineral Densitometry report for clinical interpretation." In GP Advice box add the Spine BMD value and definitions of Normal/ Osteopaenia/Osteoporosis.

6. Only if Spine BMD value is <80mg/cm3 AND NOGG advice is GREEN (after entering Femoral neck/Hip BMD into FRAX) does a Spine result dominate. In this case, the following generic CTXA Hip GP advice box text is automatically added, "Both hip and spine results are available. For this patients' clinical interpretation, please see their associated Spine 3D QCT BMD result".

GP advice text using *Mindways QCT Pro Spine L1-3 BMD value + initial FRAX score/NOGG*

ACR Spine BMD Thresholds

**Abnormal High**<br>**BMD >200mg/cm^3**	**Normal**<br>**BMD >120mg/cm^3**		**Osteopenia**<br>**120mg/cm^3 ≥ BMD > 80mg/cm^3**		**Osteoporosis**<br>**BMD ≤ 80mg/cm^3**
	**Initial FRAX Major osteoporotic fracture risk ≤ 20%**	**Initial FRAX Major osteoporotic fracture risk >20%**	**Initial FRAX Major osteoporotic fracture risk ≤ 20%**	**Initial FRAX Major osteoporotic fracture risk >20%**	
This patient had a FRAX score high enough to warrant a BMD assessment which was done using the clinical CT scan. Their BMD of (***) mg/cm3 (*) is higher than expected. High density can be due to degenerative/ genetic effects or for other reasons.	This patient had a FRAX score high enough to warrant a BMD assessment which was done using the clinical CT scan. Their spine BMD >120mg/cm3 (*) is defined as normal bone density by ACR criteria. Unless there is a change in the clinical circumstances, no routine repeat BMD tests are indicated.	This patient had a FRAX score high enough to warrant BMD assessment using the clinical CT scan. Their spine BMD >120mg/cm3 (*) which is defined as normal bone density by ACR criteria. However, there are clinical risk factors which elevate the 10 year future fracture risk (**%).	This patient had a FRAX score high enough to warrant a BMD assessment which was done using the clinical CT scan. Their SPINE BMD of 80-120 mg/cm3 (*) is defined as osteopenia by ACR criteria. There is no current indication for bone active therapy, but the patient may wish to follow the lifestyle advice to maintain bone density (if relevant to patient avoid smoking and excess alcohol intake, and maintain weightbearing exercise.) Please use the online WHO FRAX tool to determine if a standard DXA scan is needed in 3 years.	This patient had a FRAX score high enough to warrant BMD assessment using the clinical CT scan. Their SPINE BMD of 80-120 mg/cm3(*) is defined as osteopenia by ACR criteria. However, there are clinical risk factors which elevate the 10 year future fracture risk above the treatment threshold (**%). Unless contraindicated, consider bisphosphonate treatment.	This patient had a FRAX score high enough (10 year future risk **%) to warrant a BMD assessment using their clinical CT scan. Their SPINE BMD of <80mg/cm3 (*) is defined as osteoporosis by ACR criteria. We suggest investigations to rule out secondary causes (e.g. renal/liver/bone function, low vit D, high PTH, occasionally TTG, paraprotein screen). Then, unless contraindicated, consider oral or intravenous bisphosphonates as first line therapy with sufficient vitamin D & calcium. If relevant to patient, please recommend avoid smoking, excess alcohol intake, & maintain weightbearing exercise. Follow up: Please re-assess fracture risk and indication for treatment after 3-5 years
If grade 2 or 3 vertebral # identified.<br>Include:<br>Please note that a vertebral fracture was identified. Unless this was traumatic/already known, you may wish to investigate further	If grade 2 or 3 vertebral # identified.<br>Include:<br>Please note that a vertebral fracture was identified. Unless this was traumatic/already known, you may wish to investigate further	If grade 2 or 3 vertebral # identified.<br>Include:<br>Please note that a vertebral fracture was identified. Unless this was traumatic/already known, you may wish to investigate further	If grade 2 or 3 vertebral # identified.<br>Include:<br>Please note that a vertebral fracture was identified. Unless this was traumatic/already known, you may wish to investigate further	If grade 2 or 3 vertebral # identified.<br>Include:<br>Please note that a vertebral fracture was identified. Unless this was traumatic/already known, you may wish to investigate further	If grade 2 or 3 vertebral # identified.<br>Include:<br>Please note that a vertebral fracture was identified. Unless this was traumatic/already known, you may wish to investigate further

* Vertebrae analysed

** Major Fracture Risk Score

*** Average BMD value of vertebrae analysed

American College of Radiology

PHOENIX-f Clinical Decision Tree *part 2*

GP advice text using *Mindways QCT Pro CTXA Femoral neck BMD value + BMD-Adjusted FRAX/NOGG*

WHO BMD T-Score Criteria								
**Abnormal High**<br>**T-Score > 2**	**Normal**<br>**T-Score > -1**			**Osteopenia**<br>**-1 ≥ T-Score > -2.5**			**Osteoporosis**<br>**T-Score ≤ -2.5**	
**Any FRAX/NOGG Threshold**	**Below FRAX /NOGG Treatment Threshold**	**Below FRAX/NOGG Treatment Threshold AND ≥75yrs with prior fragility#/vert #**	**Above FRAX/NOGG Treatment Threshold**	**Below FRAX/NOGG Treatment Threshold**	**Either i) Below FRAX/NOGG Treatment Threshold ≥75yrs with prior fragility#/vert# OR ii) Between -2.0 & -2.50 with prior fragility#/vert#**	**Above FRAX/NOGG Treatment Threshold**	**Below FRAX/NOGG Treatment Threshold**	**Either i) Above FRAX/NOGG Treatment Threshold OR ii) Below treatment threshold with a prior fracture aged ≥75**
This patient has a FRAX score high enough to warrant a BMD assessment which was done using their clinical CT scan. Their *femoral neck/ total hip T-score was (***) which is higher than expected which can have genetic, degenerative or other causes. There is no evidence of osteoporosis	This patient had a FRAX score high enough to warrant a BMD assessment using the clinical CT scan. Their *femoral neck/total hip T-score was >-1 which is defined as normal hip bone density by WHO guidelines (FRAX score **%). Unless there is a change in the clinical circumstances, no routine repeat BMD tests are indicated.	This patient had a FRAX score high enough to warrant a BMD assessment using the clinical CT scan. Their *femoral neck/ total hip T-score was >-1 which is defined as normal hip bone density by WHO guidelines (FRAX score **%). Please note National Guidelines recommend bisphosphonate treatment in the majority of elderly people with a prior fragility fracture regardless of calculated risk. If relevant to patient, recommend avoid smoking, excess alcohol intake, & maintain weightbearing exercise.	This patient had a FRAX score high enough to warrant a BMD assessment using the clinical CT scan. Their *femoral neck/total hip T-score was >-1 which is defined as normal hip bone density by WHO guidelines. However, there are clinical risk factors which elevate the 10 year future fracture risk (FRAX score **%).	This patient had a FRAX score high enough to warrant BMD assessment using the clinical CT scan. Their *femoral neck/ total hip T-score was between -1 and -2.5 which is defined as osteopenia, or low normal bone density, by WHO guidelines. (FRAX score **%). There is no current indication for bone active therapy, but the patient may wish to follow the lifestyle advice to maintain bone density (if relevant to patient avoid smoking and excess alcohol intake, and maintain weightbearing exercise.) Please use the online WHO FRAX tool to determine if a standard DXA scan is needed in 3 years.	This patient had a FRAX score high enough to warrant BMD assessment using the clinical CT scan. Their *femoral neck/total hip T-score was between -1 and > -2.5 which is defined as osteopenia, or low normal bone density, by WHO guidelines (FRAX Score **%). Please note National Guidelines recommend bisphosphonate treatment in the majority of older people with a prior fragility fracture regardless of calculated risk. Unless contraindicated consider bisphosphonate treatment. If relevant to patient, recommend avoid smoking, excess alcohol intake, & maintain weightbearing exercise.	This patient had a FRAX score high enough to warrant BMD assessment using the clinical CT scan. Their *femoral neck/total hip T-score was between -1 and > -2.5 which is defined as osteopenia, or low normal bone density, by WHO guidelines. However, there are clinical risk factors which elevate the 10 year future fracture risk above the treatment threshold (FRAX Score **%). Unless contraindicated consider bisphosphonate treatment. If relevant to patient, recommend avoid smoking, excess alcohol intake, & maintain weightbearing exercise.	This patient's FRAX score was high enough to warrant BMD assessment using their clinical CT scan. Their *femoral neck/total hip T-score was osteoporotic (FRAX score **%). While the scan indicates osteoporosis at the hip, treatment is not automatically recommended (by National Guidelines) at this level of 10-year risk. Suggest investigations to rule out secondary causes (e.g renal/liver/bone function, occasionally TTG, paraprotein screen) The patient may wish to follow the lifestyle advice to maintain bone density (if relevant to patient avoid smoking and excess alcohol intake, and maintain weightbearing exercise.)	This patient's FRAX score was high enough to warrant BMD assessment using their clinical CT scan. Their *femoral neck/ total hip T-score was osteoporotic (FRAX score **%). Suggest investigations to rule out secondary causes (e.g renal/liver/ bone function, occasionally TTG, paraprotein screen). Then, unless contraindicated, consider oral or intravenous bisphosphonates as first line therapy with sufficient vitamin D & calcium. Follow up: Please reassess fracture risk and indication for treatment after 3-5 years
If grade 2 or 3 vertebral # identified<br>Include:<br>Please note that a vertebral fracture was identified, unless this was traumatic/already known, you may wish to investigate further	If grade 2 or 3 vertebral # identified, <75y/o only.<br>Include:<br>Please note that a vertebral fracture was identified, unless this was traumatic/ already known, you may wish to order a spine only DXA scan.	If grade 2 or 3 vertebral # identified.<br>Include:<br>Please note that a vertebral fracture was identified.	If grade 2 or 3 vertebral # identified, <75y/o only.<br>Include:<br>Please note that a vertebral fracture was identified, unless this was traumatic/ already known, you may wish to order a spine only DXA scan."	If grade 2 or 3 vertebral # identified, <75y/o only.<br>Include:<br>Please note that a vertebral fracture was identified, unless this was traumatic/already known, you may wish to order a spine only DXA scan.	If grade 2 or 3 vertebral # identified<br>Include:<br>Please note that a vertebral fracture was identified.	If grade 2 or 3 vertebral # identified<br>Include:<br>Please note that a vertebral fracture was identified.	If grade 2 or 3 vertebral # identified, <75y/o only<br>Include:<br>Please note that a vertebral fracture was identified.	If grade 2 or 3 vertebral # identified<br>Include:<br>Please note that a vertebral fracture was identified.

* Where lowest BMD T-Score was at either the Femoral Neck or Total Hip

** Value of either the Major FRAX score OR the Hip Fracture FRAX score when this value is above the FRAX/NOGG treatment threshold AND Major is below the threshold.

*** Value of the BMD T-Score

Reporting checklist for protocol of a clinical trial.

Based on the SPIRIT guidelines.

Instructions to authors

Complete this checklist by entering the page numbers from your manuscript where readers will find each of the items listed below.

Your article may not currently address all the items on the checklist. Please modify your text to include the missing information. If you are certain that an item does not apply, please write "n/a" and provide a short explanation.

Upload your completed checklist as an extra file when you submit to a journal.

In your methods section, say that you used the SPIRITreporting guidelines, and cite them as:

Chan A-W, Tetzlaff JM, Gøtzsche PC, Altman DG, Mann H, Berlin J, Dickersin K, Hróbjartsson A, Schulz KF, Parulekar WR, Krleža-Jerić K, Laupacis A, Moher D. SPIRIT 2013 Explanation and Elaboration: Guidance for protocols of clinical trials. BMJ. 2013;346:e7586

		Reporting Item	Page Number
**Administrative information**			
Title	#1	Descriptive title identifying the study design, population, interventions, and, if applicable, trial acronym	1

For peer review only

Trial registration	#2a	Trial identifier and registry name. If not yet registered, name of intended registry	1
Trial registration: data set	#2b	All items from the World Health Organization Trial Registration Data Set	1
Protocol version	#3	Date and version identifier	1
Funding	#4	Sources and types of financial, material, and other support	10
Roles and responsibilities: contributorship	#5a	Names, affiliations, and roles of protocol contributors	2
Roles and responsibilities: sponsor contact information	#5b	Name and contact information for the trial sponsor	2
Roles and responsibilities: sponsor and funder	#5c	Role of study sponsor and funders, if any, in study design; collection, management, analysis, and interpretation of data; writing of the report; and the decision to submit the report for publication, including whether they will have ultimate authority over any of these activities	2
Roles and responsibilities: committees	#5d	Composition, roles, and responsibilities of the coordinating centre, steering committee, endpoint adjudication committee, data management team, and	5

For peer review only

		other individuals or groups overseeing the trial, if applicable (see Item 21a for data monitoring committee)	
**Introduction**			
Background and rationale	#6a	Description of research question and justification for undertaking the trial, including summary of relevant studies (published and unpublished) examining benefits and harms for each intervention	3
Background and rationale: choice of comparators	#6b	Explanation for choice of comparators	5
Objectives	#7	Specific objectives or hypotheses	3
Trial design	#8	Description of trial design including type of trial (eg, parallel group, crossover, factorial, single group), allocation ratio, and framework (eg, superiority, equivalence, non-inferiority, exploratory)	4
**Methods: Participants, interventions, and outcomes**			
Study setting	#9	Description of study settings (eg, community clinic, academic hospital) and list of countries where data will be collected. Reference to where list of study sites can be obtained	3

For peer review only

Eligibility criteria	#10	Inclusion and exclusion criteria for participants. If applicable, eligibility criteria for study centres and individuals who will perform the interventions (eg, surgeons, psychotherapists)	7
Interventions: description	#11a	Interventions for each group with sufficient detail to allow replication, including how and when they will be administered	5
Interventions: modifications	#11b	Criteria for discontinuing or modifying allocated interventions for a given trial participant (eg, drug dose change in response to harms, participant request, or improving / worsening disease)	n/a
Interventions: adherance	#11c	Strategies to improve adherence to intervention protocols, and any procedures for monitoring adherence (eg, drug tablet return; laboratory tests)	n/a
Interventions: concomitant care	#11d	Relevant concomitant care and interventions that are permitted or prohibited during the trial	7
Outcomes	#12	Primary, secondary, and other outcomes, including the specific measurement variable (eg, systolic blood pressure), analysis metric (eg, change from baseline, final value, time to event), method of aggregation (eg, median, proportion), and time point for each outcome. Explanation of the clinical relevance of chosen efficacy and harm outcomes is strongly recommended	7

For peer review only

Participant timeline	#13	Time schedule of enrolment, interventions (including any run-ins and washouts), assessments, and visits for participants. A schematic diagram is highly recommended (see Figure)	n/a
Sample size	#14	Estimated number of participants needed to achieve study objectives and how it was determined, including clinical and statistical assumptions supporting any sample size calculations	7
Recruitment	#15	Strategies for achieving adequate participant enrolment to reach target sample size	n/a
**Methods: Assignment of interventions (for controlled trials)**			
Allocation: sequence generation	#16a	Method of generating the allocation sequence (eg, computer-generated random numbers), and list of any factors for stratification. To reduce predictability of a random sequence, details of any planned restriction (eg, blocking) should be provided in a separate document that is unavailable to those who enrol participants or assign interventions	9
Allocation concealment mechanism	#16b	Mechanism of implementing the allocation sequence (eg, central telephone; sequentially numbered, opaque,	n/a

For peer review only

		sealed envelopes), describing any steps to conceal the sequence until interventions are assigned	
Allocation: implementation	#16c	Who will generate the allocation sequence, who will enrol participants, and who will assign participants to interventions	9
Blinding (masking)	#17a	Who will be blinded after assignment to interventions (eg, trial participants, care providers, outcome assessors, data analysts), and how	n/a
Blinding (masking): emergency unblinding	#17b	If blinded, circumstances under which unblinding is permissible, and procedure for revealing a participant's allocated intervention during the trial	n/a
**Methods: Data collection, management, and analysis**			
Data collection plan	#18a	Plans for assessment and collection of outcome, baseline, and other trial data, including any related processes to promote data quality (eg, duplicate measurements, training of assessors) and a description of study instruments (eg, questionnaires, laboratory tests) along with their reliability and validity, if known. Reference to where data collection forms can be found, if not in the protocol	9

For peer review only

Data collection plan: retention	#18b	Plans to promote participant retention and complete follow-up, including list of any outcome data to be collected for participants who discontinue or deviate from intervention protocols	n/a
Data management	#19	Plans for data entry, coding, security, and storage, including any related processes to promote data quality (eg, double data entry; range checks for data values). Reference to where details of data management procedures can be found, if not in the protocol	n/a
Statistics: outcomes	#20a	Statistical methods for analysing primary and secondary outcomes. Reference to where other details of the statistical analysis plan can be found, if not in the protocol	9
Statistics: additional analyses	#20b	Methods for any additional analyses (eg, subgroup and adjusted analyses)	9
Statistics: analysis population and missing data	#20c	Definition of analysis population relating to protocol non-adherence (eg, as randomised analysis), and any statistical methods to handle missing data (eg, multiple imputation)	9

Methods: Monitoring

Data monitoring: formal committee	#21a	Composition of data monitoring committee (DMC); summary of its role and reporting structure; statement of whether it is independent from the sponsor and competing interests; and reference to where further	n/a

For peer review only

		details about its charter can be found, if not in the protocol. Alternatively, an explanation of why a DMC is not needed	
Data monitoring: interim analysis	#21b	Description of any interim analyses and stopping guidelines, including who will have access to these interim results and make the final decision to terminate the trial	n/a
Harms	#22	Plans for collecting, assessing, reporting, and managing solicited and spontaneously reported adverse events and other unintended effects of trial interventions or trial conduct	n/a
Auditing	#23	Frequency and procedures for auditing trial conduct, if any, and whether the process will be independent from investigators and the sponsor	n/a
**Ethics and dissemination**			
Research ethics approval	#24	Plans for seeking research ethics committee / institutional review board (REC / IRB) approval	10
Protocol amendments	#25	Plans for communicating important protocol modifications (eg, changes to eligibility criteria, outcomes, analyses) to relevant parties (eg, investigators, REC / IRBs, trial participants, trial registries, journals, regulators)	1

For peer review only

Consent or assent	#26a	Who will obtain informed consent or assent from potential trial participants or authorised surrogates, and how (see Item 32)	5
Consent or assent: ancillary studies	#26b	Additional consent provisions for collection and use of participant data and biological specimens in ancillary studies, if applicable	n/a
Confidentiality	#27	How personal information about potential and enrolled participants will be collected, shared, and maintained in order to protect confidentiality before, during, and after the trial	5
Declaration of interests	#28	Financial and other competing interests for principal investigators for the overall trial and each study site	10
Data access	#29	Statement of who will have access to the final trial dataset, and disclosure of contractual agreements that limit such access for investigators	10
Ancillary and post trial care	#30	Provisions, if any, for ancillary and post-trial care, and for compensation to those who suffer harm from trial participation	n/a
Dissemination policy: trial results	#31a	Plans for investigators and sponsor to communicate trial results to participants, healthcare professionals, the public, and other relevant groups (eg, via publication, reporting in results databases, or other data sharing arrangements), including any publication restrictions	10

For peer review only

Dissemination policy: authorship	#31b	Authorship eligibility guidelines and any intended use of professional writers	n/a
Dissemination policy: reproducible research	#31c	Plans, if any, for granting public access to the full protocol, participant-level dataset, and statistical code	10
**Appendices**			
Informed consent materials	#32	Model consent form and other related documentation given to participants and authorised surrogates	12
Biological specimens	#33	Plans for collection, laboratory evaluation, and storage of biological specimens for genetic or molecular analysis in the current trial and for future use in ancillary studies, if applicable	n/a

The SPIRIT Explanation and Elaboration paper is distributed under the terms of the Creative Commons Attribution License CC-BY-NC. This checklist was completed on 16. February 2021 using https://www.goodreports.org/, a tool made by the EQUATOR Network in collaboration with Penelope.ai

For peer review only

BMJ Open

PHOENIX (Picking up Hidden Osteoporosis Effectively during Normal CT Imaging without additional X-rays). Protocol for a randomised, multi-centre feasibility study

Journal:	*BMJ Open*
Manuscript ID	bmjopen-2021-050343.R1
Article Type:	Protocol
Date Submitted by the Author:	01-Feb-2022
Complete List of Authors:	Poole, Kenneth; University of Cambridge School of Clinical Medicine; NIHR Cambridge Biomedical Research Centre<br>Chappell, Daniel; Cambridge University Hospitals NHS Foundation Trust, Rheumatology; NIHR Cambridge Biomedical Research Centre<br>Clark, Emma; University of Bristol, Clinical Science at North Bristol<br>Fleming, Jane; University of Cambridge, Cambridge Institute of Public Health; University of Cambridge, Department of Public Health and Primary Care<br>Shepstone, Lee; University of East Anglia<br>Turmezei, Tom; Cambridge University Hospitals NHS Foundation Trust, Radiology; Norfolk and Norwich University Hospital NHS Trust, Radiology<br>Wagner, Adam; NIHR Collaboration for Leadership in Applied Health Research & Care (CLAHRC) East of England; University of East Anglia, Norwich Medical School<br>willoughby, karen; University of Cambridge School of Clinical Medicine, Medicine; NIHR Cambridge Biomedical Research Centre<br>Kaptoge, Stephen; University of Cambridge
**Primary Subject Heading**:	Radiology and imaging
Secondary Subject Heading:	Rheumatology, General practice / Family practice
Keywords:	Bone diseases < ORTHOPAEDIC & TRAUMA SURGERY, Spine < ORTHOPAEDIC & TRAUMA SURGERY, Computed tomography < RADIOLOGY & IMAGING, Diagnostic radiology < RADIOLOGY & IMAGING, RHEUMATOLOGY, Back pain < ORTHOPAEDIC & TRAUMA SURGERY

SCHOLARONE™
Manuscripts

PHOENIX (Picking up Hidden Osteoporosis Effectively during Normal CT Imaging without additional X-rays). Protocol for a randomised, multi-centre feasibility study

Authors Kenneth E. S. Poole* (a, b), Daniel DG Chappell (a), Emma M Clark (c), Jane Fleming (b), Lee Shepstone (d), Thomas D Turmezei (a, e) Adam P Wagner (d, f), Karen Willoughby (b), Dr Stephen K Kaptoge (b).
***Corresponding**
(a) Cambridge University Hospitals NHS Trust, (b) University of Cambridge, (c) University of Bristol, (d) University of East Anglia, (e) Norfolk and Norwich University Hospitals NHS Trust, Norwich, (f) National Institute of Health Research (NIHR), Applied Research Collaboration (ARC) East of England (EoE),

Address: Dept of Medicine, University of Cambridge, Level 7, Hills Rd, Cambridge CB2 0QQ **Tel:** 01223 245151 **Email:** kenneth.poole@nhs.net

Key words: Osteoporosis; osteoporotic fracture reduction; opportunistic screening; vertebral fracture.

Introduction: Two million out of the UK's 5 million routine diagnostic computed tomography (CT) scans performed each year incorporate the thoraco-lumbar spine or pelvic region. Up to one third reveal undiagnosed osteoporosis or vertebral fractures. We developed an intervention, 'PHOENIX', to facilitate early detection and management of osteoporosis in people attending hospitals for CT scans.
Methods & analysis: A multi-centre, randomised, pragmatic feasibility study. From the general CT-attending population, women ≥65 and men ≥75 years attending for CT scans are invited to participate, via a novel consent form incorporating FRAX Fracture Risk Assessment questions. Those at increased 10-year risk (within the amber or red zones of the UK FRAX graphical outputs for further action) are block randomised (1:1:1) to 1) PHOENIX intervention, 2) Active Control or 3) Usual Care. The PHOENIX Intervention comprises i) retrieving the CT scans using the NHS Image Exchange Portal, ii) Mindways QCT Pro software analysis of CT Hip and Spine Bone Density with CT Vertebral Fracture Assessment, iii) sending the participants' GP a clinical report including diagnosis, necessary investigations, and recommended treatment. Baseline CT scans from groups 2 and 3 are assessed with the PHOENIX intervention at study end. Assuming 25% attrition, the study is powered to find a predicted superior osteoporosis treatment rate with PHOENIX (20%) versus 16% among patients whose GPs were sent the FRAX questionnaire only (Active Control) and 5% in the Usual Care group. Five hospitals are participating, to determine feasibility. The co-primary feasibility outcome measures are a) ability to randomise 375 patients within 10 months and b) retention of 75% of survivors, completing their 1-year bone health outcome questionnaire. Secondary 1-year outcomes include osteoporosis/vertebral fracture identification rates and osteoporosis treatment rates. Stakeholder acceptability and economic aspects are evaluated.
Ethics and dissemination REF19/EE/0176. Dissemination will be through the Royal Osteoporosis Society.
Trial registration number 14722819

Strengths and limitations of this study:

- First pragmatic randomised controlled study of patients attending radiology CT units, who are randomised to either comprehensive screening for osteoporosis (by applying FRAX questionnaire, CT bone densitometry and CT vertebral fracture detection) or usual care.

For peer review only - http://bmjopen.bmj.com/site/about/guidelines.xhtml

- Allows patients the flexibility to self-consent, and tests both the willingness of CT attenders to consent, CT technologies and information flow between trial centres and Cambridge hub
- Limited by an amendment of the primary outcome measure because recruitment during COVID-19 pandemic predicted skew towards the more seriously ill (via cancellation of non-urgent and routine scans). Modifications to both the retention outcome and longer recruitment window were made to take account of pandemic redeployment of medical research team.

Introduction: Older patients (65 years ≥in women, 70 years ≥in men) attending hospital for CT scans may have undiagnosed spine fractures or low bone density and therefore stand to benefit from osteoporosis screening. Timely diagnosis and treatment of osteoporosis benefits many patient outcome domains (pain, quality of life, morbidity, and in the case of zoledronate treatment, mortality). An estimated 43% of over 60-year-olds undergoing CT scans (for abdominal and pelvic problems unrelated to their bones) were found to have osteoporosis or vertebral fractures when scan images were examined using computer-analysis methods[1]. However, radiologists reviewing CT scans reported only 16% of vertebral fractures in routine practice[2], and bone density (BMD) is rarely calculated from CT scans (CT BMD). This is despite CT BMD being approved internationally for the diagnosis of osteoporosis. Also, femoral neck BMD from CT is interchangeable with standard DXA* BMD when using the global individual fracture risk web calculator FRAX (*Dual Energy X-ray Absorptiometry). Indeed, several approved software packages now allow accurate diagnosis of osteoporosis in the hip and spine from routinely acquired CT images, while at the same time facilitating vertebral fractures identification and grading.

Systematic reviews indicate that overlooking osteoporosis or vertebral fractures can lead to serious, preventable pain, disability and health costs (>£2 billion per year from osteoporosis in the UK)[3]. Osteoporosis is a common disease in older women and men: osteoporosis is associated with >200,000 vertebral fractures and >85,000 hip fractures annually in the UK[4]. Osteoporosis-related fractures have a high morbidity and, despite low cost-effective treatments, women and men are increasingly presenting at clinics with advanced multiple vertebral fractures, loss of body height, compressed abdominal contents, dysphagia, severe and chronic pain and reduced mobility. Despite an ageing UK population, osteoporosis remains under-diagnosed and under-treated[5]. The osteoporosis case-finding rate in primary care is low and there is no national mandate for screening. High risk patients may be referred for DXA and are offered treatment if fracture risk sufficiently high. Vertebral fractures are infrequently detected in usual care even though they (alongside hip fractures) cause the biggest burden to patients and the health service. We hypothesise that targeted screening will improve upon usual care and improve diagnosis. The PHOENIX intervention is a simple and widely practicable intervention to facilitate osteoporosis identification, diagnosis and treatment in patients attending for routine clinical CT at any hospital. It has been developed to apply software to CT scan images and repurpose them for measuring bone density, fracture risk and to identify crushed vertebrae[6,7]. It couples timely diagnosis with an individualised report (built from a guideline-derived clinical decision tree) sent to GPs containing advice on recommended investigations, NICE-approved, cost-effective treatment/lifestyle advice to reduce osteoporotic fractures[8]. We expect benefits to accrue through early diagnosis and treatment of osteoporosis, with additional justifications for investigating this population shown in Table 1. Preventing fractures could lead to decreased societal and social burden, and reduced health- and social-care costs.

**Table 1. Opportunistic osteoporosis screening of CT scan attenders and rationale for PHOENIX Intervention**
• CT attenders are a high-risk population: >30% of older adults have undiagnosed vertebral fractures or osteoporosis that can be effectively treated once identified[9]

For peer review only - http://bmjopen.bmj.com/site/about/guidelines.xhtml

•	The PHOENIX intervention diagnoses osteoporosis on scans already undertaken, so there is no requirement for additional x-ray exposure or hospital attendance
•	The PHOENIX intervention can be applied to avoid the necessity of extra DXA visits and DXA reporting in multiple clinical scenarios, such as in the setting of radiology reporting Quality Improvement Programmes that increase CT-diagnosis of vertebral fractures, or to formally diagnose patients with osteoporosis after largescale Artificial Intelligence (AI) vertebral fracture identification methods (e.g. Optasia or ZEBRA AI1tools)
•	Screening is effective at reducing hip fractures. A recent trial of primary screening based on clinical risk factors and targeted bone density scanning reduced the hip fracture incidence by 28% over five years, with a number needed to screen (NNS) of 111 to prevent one hip fracture[10]
•	Systematic reviews of screening for osteoporosis suggest a number needed to screen (NNS) of 43 people (aged 75 or over) to prevent one vertebral fracture[11]
•	Fracture risk is currently assessed opportunistically by GPs. Current advances in IT software could augment this practice to make more efficient and effective use of healthcare resources, as recommended by the National Institute for Health and Care Excellence (NICE), Clinical Guideline (CG) 146[11]
•	A 2013 study found that radiologists failed to report 84% of clinically important vertebral fractures from routine CT scans[4]. However, once combined with new computerised analysis methods, CT scanners can effectively determine bone density and fractures [7]
•	There is a fourfold risk of another vertebral fracture occurring after the first one[12], which may indicate that osteoporosis is a quickly progressing disease after a first fracture occurs
•	Generalisability: one million CT scans were performed in the UK in 1996, increasing to five million in 2016[13], permitting screening of many 'at-risk' patients. Interventions such as PHOENIX are not complicated, nor technically demanding for those hospital staff who routinely analyse DXA scans

Methods and Analysis:

This feasibility study aims to test our PHOENIX intervention, which begins by offering adults attending hospitals for CT (for any reason) a comprehensive bone health screen, followed by 'opportunistic' reuse of their CT scans for bone densitometry. The intervention ends with their GPs receiving detailed written advice on managing the participants' bone health. Patients are offered an invitation pack comprising a novel informed consent form/data capture tool that the patient can fill out with or without assistance, and a participant information sheet. Specifically, we want to determine whether it is feasible for our team to undertake a large trial of the PHOENIX intervention to prevent fractures in a larger population of CT-attenders. Hence our co-primary outcome measure is our ability to randomise 375 'high risk' participants within 10 months. For the larger fracture prevention trial to be feasible, the other co-primary outcome measure must be achieved, which is retention at 12 months; our ability to record bone active treatment data (yes/no) in 75% of surviving patients. Power calculations for any future trial will be informed by the secondary outcome measure; the percentage of participants in each arm who have i) received a treatment recommendation (due to osteoporosis, vertebral fracture or high FRAX risk) and ii) received and commenced treatment 12 months after their CT scan. Table 2 contains a summary of the key features of the study protocol.

The PHOENIX study is a parallel three group, non-blinded randomised controlled study of volunteers aged ≥65-90 (women) or ≥75-90 (men) attending for CT for any clinical reason, where the spine and/or hips are visible in scan images. Specifically, CT-attending patient volunteers who have an elevated 10-year fracture risk (within the amber or red zones of the UK FRAX graphical outputs for further action) are randomised to one of three groups. The three arms allow comparison between (1) the PHOENIX intervention, (2) an alternative Active Control 'middle' group (to assess whether GPs will investigate or treat patients based solely on receipt of a populated FRAX questionnaire), and (3) a control ('Usual Care') group where no action is taken. Potential participants are identified ahead of attendance by the clinical or

For peer review only - http://bmjopen.bmj.com/site/about/guidelines.xhtml

research team reviewing CT lists. Those eligible by age, sex and region of the body being scanned are handed the PHOENIX pack whilst attending the CT department. The specially designed Informed Consent Form (ICF; Supplemental material file 1) first guides the potential participant through eligibility screening questions (*Do you take medication for your bones once a week that requires you to sit or stand upright? Do you have a yearly infusion (drip) or injection for your bones? Do you have a hip replacement? Have you ever had a hip operation?*). If there is an affirmative answer to any of these questions, individuals are asked to return their forms and go no further. Those individuals who answer 'no' to all the questions, are asked to supply key demographic data: Date of birth, height, weight, country of origin and ethnicity (needed for accurate FRAX calculation) and NHS number (receptionists support the patient as needed with the latter). Individuals are also asked to document the reason for their scan, if they know it.

Table 2. Summary of key features of the PHOENIX Study Protocol
**Special pragmatic features:**
Patients attending CT waiting rooms are offered a PHOENIX invitation pack comprising a novel informed consent form/data capture tool to ascertain FRAX score (Supplementary Figure 1) that the patient can fill out with or without assistance
The intervention (tested in Group 1) comprises; i) retrieving the CT scans of those 'amber' or 'red' risk individuals with moderate to high 10-year fracture risk by FRAX, ii) hip and spine BMD analysis with VFA and iii) sending the GP guidance from a Clinical Decision Tree
The intervention take place 'behind the scenes' without impacting upon patient flow or the radiography/radiology workflow
Data (imaging, clinical risk factor, PID including sensitive clinical information) flows via secure encrypted and approved methods including the NHS Image Exchange Portal, PACS and nhs.net secure clinical email
Technical staff can 'pull' clinical CT images for assessment at any time
**Primary Outcome measures/endpoints:**
Feasibility of recruitment; Defined as ability to randomise 375 'at risk' participants over continuous 10-month period
Retention: Defined as retaining 75% of surviving participants to 12 months post randomisation, proven by receipt of follow-up postal questionnaire (specifically an answer to the bone active medication received; yes/no question)
**Secondary Outcome measures/endpoints:**
New vertebral fracture identification across 3 arms assessed at 12 months
Osteoporosis identification across 3 arms assessed at 12 months
Bone active medication initiation across the 3 arms assessed at 12 months
Survival at 12 months across 3 arms assessed at 12 months
**Inclusion criteria: Participants eligible for the trial should fulfil all of the following at randomisation:**
Attending for a routine clinical CT scan
Able to provide informed consent
Aged ⩾65-90 (women) or ⩾75-90 (men) attending for CT for any clinical reason, where the spine and/or hips are visible in scan images
**Exclusion criteria:**
Metalwork in hips (due to streak artefact on the contralateral hip within CT)
Known to be receiving prescription treatment for osteoporosis other than calcium/vitamin D (i.e., bisphosphonate drug, strontium ranelate, denosumab, raloxifene or teriparatide since it would be futile to identify already known and treated osteoporosis)
Prone CT scan (which cannot currently be processed by the software)

For peer review only - http://bmjopen.bmj.com/site/about/guidelines.xhtml

Role of patients and the public
Actively participated in the study design in focus groups within the project team
Assisted in the design of data collection tools, including the Informed Consent Form and Information Leaflet
Provided guidance on the acceptability of approaching participants in CT scan areas and insight into the anxiety of attending hospital amongst some more elderly members of society. Adding Country of Origin and Ethnicity questions to consent form to facilitate FRAX.
**Impact of COVID-19 pandemic**
Recruitment had to be paused due to the COVID-19 pandemic, then restarted in just one centre without face-to-face contact with patients. Therefore, recruitment was either by clinical approach/discussion versus self-consent based on just handing out recruitment packs after covid-19 restrictions were brought in.

On the last page of the consent form, the eight validated bone health-relevant questions from the FRAX questionnaire are completed, along with patient address, telephone number, GP name and GP address. Completed paperwork is scanned and sent by email attachment within the national, clinically secure NHS.net email account infrastructure to our add-tr.phoenixs@nhs.net address (except for local patients where the paperwork is collected directly from the CT reception). FRAX answers are processed by the research team at Cambridge who use the results to calculate the 10-year fracture risk score *without* femoral neck BMD online. Those at low risk of fracture (within the green zone of the UK FRAX graphical outputs) are not randomised, but for completeness, these low-risk participants do eventually have the bone parameters in their CT scans reviewed. CT scans from participants in this 'green' category are retrieved later and put through the PHOENIX intervention 13 months after they consented, allowing the study team to ascertain whether FRAX had been effective in picking up patients with osteoporosis/high fracture risk in this CT waiting room setting. Those in the remaining 'red' and 'amber' risk categories are randomised to one of 3 arms, described below: Group 1) The PHOENIX intervention, 2) Active Control (FRAX questionnaire results only sent to GP) and 3) Usual Care. The flow of patients through the study, including randomisation is shown in figure. 1.

Group 1. The PHOENIX intervention. In brief, bone health for the red/amber risk participant is assessed using the CT Images already captured, with computer-aided diagnosis of vertebral fractures performed by one of two trained NHS and accredited bone density technicians using Mindways QCT Pro (Mindways, Austin, Texas USA) in Cambridge. The output is a consultant clinician-verified report (Figure. 2) sent to the GP with details of i) femoral neck and/or spine bone density results, ii) vertebral fracture diagnosis (yes/no/level affected), iii) FRAX estimated 10-year risk of major osteoporotic fracture, iv) any recommended follow-up investigations and a v) simple treat/do not treat decision based on NOGG intervention thresholds, with a suggestion of specific treatments recommended by NICE guidelines. These steps can take place at any time after the patient has gone home from having their CT. Any CT scanner to be used for CT densitometry must be calibrated by reference to a QCT phantom and recalibrated in the event of any x-ray tube change. The technical aspects of the PHOENIX intervention done at study entry in Group 1 (and at study exit in the others two groups) are as follows; all analysis is conducted within the Mindways QCT Pro software tool using one of two standalone hospital-connected PCs as illustrated by screenshots (Figure 3). Technical steps involved in the full PHOENIX intervention are in Table 3.

Table 3. The PHOENIX Intervention (Group 1): Technical and Practical Steps
1) CT scan retrieved via PACS into Mindways via it DICOM transfer tool (Cambridge) or in the case of the spoke sites, via the UK NHS Image Exchange Portal to Cambridge, followed by importing to Mindways QCT Pro.

For peer review only - http://bmjopen.bmj.com/site/about/guidelines.xhtml

2)	The Mindways QCT Pro SlicePick Vertebral Fracture Assessment tool is used to identify any fractures in the imaged CT volume during the bone density assessment, by applying 6-point morphometry criteria to the synthesised lateral image (upper panels of Figure 3). SlicePick works by synthesising AP and sagittal images from the axial CT slices.
3)	Mindways QCT Pro CTXA module is used to measure BMD in the left femoral neck (Figure 3, middle panes), left total hip region (if coverage is sufficient) and/or lumbar spine (L1-L3 as per International Society for Clinical Densitometry (ISCD) guidelines, lower panes Figure 3).
4)	Each individual's FRAX fracture risk estimate is then recalculated online, using clinical RF's from FRAX and the femoral neck BMD value. Importantly, the 'Previous fracture' category is switched to 'yes' in FRAX if spine imaging revealed any vertebral fracture.
5)	The NOGG guidance tool button within FRAX is then used and the pop-up red/green risk charts followed to determine whether treatment is recommended, provided no contra-indications exists. In the case of no femoral neck BMD possible (spinal BMD only), FRAX values are reported (without BMD input) and the PHOENIX clinical decision tree for spine BMD is followed to produce the report that is sent to GPs.
6)	The clinician-verified report phrases are derived from the PHOENIX clinical decision tree (Supplemental material file 2) which is stored within the QCT Pro reporting tool and updated regularly. The report phrases appear (e.g. in Figure 2) on the Hip pdf (CTXA™ Hip Bone Mineral Densitometry, 'GP Advice') analysis and the Spine pdf (Spine 3D QCT Bone Mineral Densitometry 'GP Advice'). The technicians can select the correct GP advice phrase via drop-down menus when they have the FRAX and BMD results to hand. By selecting the matching patient category in the software, a bespoke template is imported into the pdf of the bone density report incorporating all the measured bone health information and clinical risk factors, based on the National Osteoporosis Guideline recommendations.
7)	The final Hip and Spine pdf is signed off electronically by the consultant physician (KESP) via nhs.net email (secure clinical, encrypted) and posted to GPs with a cover letter.
8)	Any vertebral fractures overlooked by radiologists on the RIS report are entered in the local radiology discrepancy review process and the original CT report addended accordingly.
9)	Responsibility for subsequent investigations including referrals for DXA and/or treatment, remain with the GP.
10)	A small, cylindrical Mindways model 4 (asynchronous CT calibration phantom) must be scanned at intervals on each CT scanner, including at the various kVP energies used clinically, according to a Standard Operating Procedure. This phantom can be posted or taken to radiology departments for QA (Quality Assurance) scanning.

Group 2. Active Control. Only the completed FRAX questionnaire results are sent to the GP, who can follow NOGG guidance themselves by entering responses onto the online FRAX website calculator. However, the patient's data is put through the PHOENIX intervention at study exit (13 months) and results shared with the GP then to enable them to make informed decisions regarding possible treatment.

Group 3. Usual Care. The GP receives a letter informing them of their patient's involvement in the study, but no further reports or information at this point. However, the patient's data is put through the PHOENIX intervention at study exit (13 months) and results shared with the GP then to enable them to make informed decisions regarding possible treatment.

Baseline and one-year follow-up postal questionnaires are sent to all randomised participants to collect data on quality of life and resource use relevant to bone health for health economic analysis. For non-responders a telephone interview is attempted aiming to collect the questionnaire data. Patient and clinician/healthcare worker/administrator perspectives of study involvement are explored through semi-structured interviews by telephone. All study data are held securely processed in accordance with the Data Protection Act 2018 and not disclosed to third parties. Manual records will be held securely (for example in locked filing cabinets). Electronic records will be held on a secure network requiring user

For peer review only - http://bmjopen.bmj.com/site/about/guidelines.xhtml

ID and password access. Individuals are not identifiable from the REDCAP and JMP (v15, SAS Institute) spreadsheet results of the trial. The Project Management Team members (KW, KESP, DDGC, JMB) are responsible for the day to day running of the study to discuss practical aspects and ensure it is progressing. The Trial Management Group (TMG) comprises the protocol authors and one PPI member. They are responsible for the overall conduct of the study and ensuring NIHR milestones are achieved. Prior to Covid-19 separate PPI meetings were held which enabled those attending to influence the study design.

Sample size calculations, Study power, Randomisation method

Our primary intention is to evaluate the technical procedures needed for a definitive trial with fracture incidence (rather than recruitment/retention) as the primary outcome. To help set the target recruitment numbers for the feasibility study, we therefore used the surrogate outcome of osteoporosis treatment rates at 12 months follow up to permit sufficient power to assess and conduct cross-group comparisons across trial arms within the period of recruitment to the feasibility study. Scenarios for scaling up to a definitive trial are based on previous data [1, 2, 14]. Only those participants in the red/amber FRAX group (Figure 4a) will be randomised, so the number of invitations needed to reach any recruitment target will also depend on the prevalence of the red/amber FRAX group. In our local preliminary application of the PHOENIX intervention in 1518 patients, the prevalence of red/amber FRAX varied between 30% at age 40 and 50% at age 95 (Figure 4b), with overall prevalence 40.5% (95% CI: 35.7%, 45.5%). There is no published UK data to compare with this but comparable prevalence was reported in a single study in the USA investigating combined bone density and fracture detection in routine CT investigations, where 42.3% (of 571 consecutive patients) were newly detected as having osteoporosis[1]. A conservative assumption from our preliminary work is that the PHOENIX Intervention will identify a further 9.6% of screened patients for treatment and that GPs will act to treat at least half of these, resulting in an expected 4.8% minimum detectable difference. If, in our study, the percentage treated at 12 months in Group 2 (Active Control FRAX-GP arm) matches that observed in the MRC-funded osteoporosis screening SCOOP study[6] (15.5%), then we would consider that an improvement in percentage treated of 4.8% would be clinically significant. Figure 4c. shows the estimated total number of invitees needed to recruit the 375 red/amber risk participants, assuming different response rates and different prevalence estimates. In the scenario of 30% response (similar to SCOOP) combined with 40% prevalence of red/amber FRAX (red line in fig 4d) it would be necessary to invite a total of 3,125 participants (or 625 invites per centre) to achieve the recruitment target of 375 randomised participants (75 per centre, Figure 4d.) **Power for recruitment and retention.** Hypothesising that the recruitment (i.e. consent, FRAX completion, eligibility, and CT imaging i.e. 'Response') would be similar to that observed in the SCOOP primary prevention trial (p0 = 30%); a feasibility study aiming to randomise 375 of 938 (40%) consenting participants (i.e. 30% of 3125 total invited) would estimate the trial's response rate with a precision of 1.6% (i.e. half-width of the 95% confidence interval, Figure 4e *Sample size for 80% power to reject null hypothesis of 30% response rate*). The randomised sample of 375 patients will provide 80% power to detect a difference of at least 6% or larger in the 12-month overall trial retention rates from a hypothesised 75% retention (as with the SCOOP trial) based on two-sided one-sample test of proportion at 5% significance level (i.e. providing statistical evidence that the overall retention may be as low or high as 69% (0.69) or 81% (0.81) respectively, as opposed to 75%, 0.75, Figure 4f = *Sample size for 80% power to reject null hypothesis of 75% retention*). In addition, against the same expectation, the 1:1:1 randomised comparison (i.e. PHOENIX Intervention vs. Active Control FRAX to GP vs. Usual Care pathways) will have >80% power to detect at least 12.2% absolute difference in retention rates between trial arms (or RR = 1.16) based on two-sided two-sample test of proportion at 5% significance level. **Power for treatment proportions.** Assuming that osteoporosis treatment percentages at 1-year follow up in the Usual Care vs.

For peer review only - http://bmjopen.bmj.com/site/about/guidelines.xhtml

Active Control FRAX-GP vs. PHOENIX Intervention would be 4.5% vs. 15.5% vs. 20.3% respectively (i.e. as observed in SCOOP trial vs. with expected improvement in PHOENIX), a total of 375 participants recruited and randomised in the ratio 1:1:1 will have 96% power (Figure 4g) to detect linear trend in treatment proportions based on a two-sided test of binomial proportions at 5% statistical significance level. The same sample size will have 93% power for a secondary two-degree of freedom test of between group contrasts in comparison with Usual Care. When allowing for up to 25% attrition due to non-differential dropout or incompleteness of outcomes, the remaining sample size of 280 participants will have >84% power to detect the same magnitude of differences (Figure 4g). **Randomisation method.** Knowing that 375 participants were needed, we were advised to undertake block randomisation stratified by centre, sex and age group (Female: <75 vs ≥75, Male: <80 vs ≥80). Randomisation utilises the web application Research Electronic Data Capture (REDCap https://www.project-redcap.org/).

Statistical analysis Daily screening logs will be kept determining: i) the total number of days (n) where patients were approached in each centre; and ii) the daily totals of PHOENIX invitation packs distributed. This log will also record if a numbered pack was not given out on a day (e.g. if the patient did not arrive for scanning, or the receptionists omitted to give out the pack). The total number of consented individuals (x) as well as the number of consented individuals per day (total x/n overall, and per centre) will be calculated. The number of consented but subsequently excluded individuals (y) will be kept (e.g. for subsequently discovered metalwork in the scan, technical issues with scan). The number of randomised participants will be calculated (z= x-y), and the rate of randomisation (z/n overall, and per centre). These data will be captured from screening logs and entered manually onto a database. The REDCap database will be used to record details of all consenting patients (z). The database will be locked on completion of data entry and when missing data or any discrepancies are resolved. The data will be exported for statistical analysis to the study statisticians with assistance from the Chief Investigator. **Predefined Statistical Analysis Plan**: Response rates will be calculated as the proportion of participants invited who (a) consent to take part, (b) meet eligibility criteria, and (c) are randomised, with 95% confidence intervals to assess the extent to which the realised response deviates from the expected as detailed in the power calculations above. Cumulative participant accrual over time will be plotted overall and by recruitment centre to assess any major differences in accrual and assess progress toward target. Logistic regression will be used to compare randomised groups with respect to the 12-month outcomes of treatment rates and participant retention. Baseline stratification covariates (i.e. centre, sex, and age) will be adjusted for in the models. Analyses will be on an intention-to-treat basis.

Qualitative analysis and Stakeholder acceptability research

Both the baseline and one year follow up questionnaires being sent to all patients include space for participants to feedback their experience of the study. First explaining how this will help, this section includes example open questions such as *"Do you have any comments on being offered a bone health check at the same time as your CT scan? Or on taking part in this research study? /how you were asked to join? /your decision to take part?".* Semi-structured interviews will be used with a smaller sub-sample of study participants (n=20) to allow for more in-depth exploration of issues that participants may raise in written feedback or during interview. Recordings will be transcribed by an academic transcription service following a confidentiality contract and anonymised by a member of the research team (JF) changing names of people and places and any identifiable details. Key research questions for the PHOENIX process evaluation are; i) what are the implementation facilitators and barriers for the PHOENIX Intervention? ii) how acceptable to the intended patient group is the process of PHOENIX screening? iii) how do implementation and acceptability issues affect uptake of the CT bone health screening and any subsequent GP investigation and recommended bone protective

For peer review only - http://bmjopen.bmj.com/site/about/guidelines.xhtml

treatment? A combination of interviews and focus groups with those staff groups who will be key to the effective delivery of PHOENIX screening will also be undertaken, both in hospital and primary care, in order to understand their different perspectives. These staff groups are research nurses, receptionists and radiographers in CT departments and GPs. Analyses will be descriptive. The software programme NVivo v12 (QSR) will be used to manage and index the qualitative data (anonymised transcripts of audio-recordings from interviews and focus groups) before charting, mapping and interpretation taking a framework approach[15] Ritchie & Spencer thematic analysis is an approach suitable for qualitative research in which data collection is relatively focussed on key questions[16,17], hence it is applicable to PHOENIX.

Patient and Public Involvement

At the 'idea' stage of the research, our small team presented our idea at the large workshop *"Find your voice; Demystifying research and public involvement in research"* at Cambridge Professional Development Centre Trumpington in May 2015. This was run by Dr Doreen Tembo PPIE lead for the Eastern region NIHR Research Design Service in partnership with the Norfolk and Suffolk PPIRes team (Public and Patient Involvement in Research). This connected us with patients and public representatives, many of whom had relevant musculoskeletal disorders and/or past experience in an orthopaedic service. We collated the opinions and suggestions on our proposed project from every participant in the workshop in writing, individually and from small group discussions to help develop our research question and outcome measures, so that they were informed by patients' priorities, experience, and preferences .Three individuals, Mr Jeremy Dearling, Mrs Tessa Plume and Dr Ann Frost volunteered to join our trial group as PPI representatives; they were specifically involved in patient documentation design (particularly the PHOENIX pack, informed consent form which facilitated consent without having a researcher present) and contributed to suggestions for increasing patient recruitment and follow up. Jane Fleming facilitated group sessions and individual structured interviews to assess the burden of the PHOENIX intervention on patients themselves, as well as a qualitative assessment of the process from a user perspective. The results of the research will be disseminated to surviving participants after generating patient-friendly materials in collaboration with our PPIE representatives.

Economic analysis

Completion rates on the quality-of-life measure (EQ-5D-5L) and resource use items included within the postal survey will inform whether an economic evaluation is feasible using these data collection approaches in the definitive trial. Patterns of response and non-response will be explored to see if they suggest ways the postal survey can be refined to improve data completeness.

Word Count: 3722 (excludes abstract, tables, figures, and references)

Legends for figures:

Figure 1. Study Flow chart for the PHOENIX study

Figure 2. Written clinical guidance and report produced at the end of a PHOENIX Intervention in a typical patient from Group 1. These comprise the standard output pdf's of Mindways QCT Pro which are sent to the participants' GP. In this case there are both hip and spine results from a typical patient with a previously undiagnosed grade 3 vertebral fracture of T12 (found and graded using SlicePick). The femoral neck BMD T-score is -3.03

For peer review only - http://bmjopen.bmj.com/site/about/guidelines.xhtml

and spinal osteoporosis is shown with L1-L3 volumetric BMD of 63.4mg/cm^3. Their FRAX 10-year risk of fracture is 25% and investigation/treatment is recommended by NOGG. The written clinical guidance is derived from the PHOENIX clinical decision tree (Supplemental file 2)

Figure 3. The PHOENIX Intervention: technical steps involved in Vertebral Fracture Assessment using Mindways SlicePick to automatically create the sagittal synchronised image in a patient where the technician has used embedded 6-point morphometry to grade a previously undiagnosed T12 vertebral fracture (upper panels). Note the radiopaque bowel contrast in AP and lateral SlicePick views. SlicePick is then used to create a volume for femoral neck (CTXA™) BMD and spine volumetric BMD measurements from the same CT scan. The middle panels show screenshots from CTXA™ femoral neck areal BMD (g/cm^2) analysis from CliniQCT. Note that in this case, only the femoral neck BMD value is taken forwards to the report/guidance (Figure 2), since the CT scan did not cover sufficiently far below the lesser trochanter to allow for 'total hip' BMD measurement. The lower panels show 3D QCT analysis of Lumbar spine L1, L2 and L3 trabecular volumetric BMD (mg/cm^3). Note that Lumbar Spine volumetric BMD T-scores are *not* used in the written clinical guidance and report shown in Figure 2, since trabecular bone spinal vBMD T-scores are not interchangeable with DXA-derived T-scores.

Figure 4. FRAX, sample size and power calculations for PHOENIX a) FRAX UK age-specific fracture risk assessment and intervention thresholds, b) Age-specific prevalence of red/amber FRAX risk category from preliminary local application of PHOENIX intervention in Cambridge, c) Calculation of the number of invites needed to randomise 375 red/amber risk participants based on different responses (rates of agreeing to participate), d) Number of PHOENIX invitation packs that must be given out in order to randomise 375 red/amber risk participants based on different prevalence of red/amber FRAX among participants, e) Our feasibility study aiming to randomise 375 of 938 (40%) consenting participants (i.e. 30% of 3125 total invited) would estimate the trial's response rate with a precision of 1.6% (i.e. half-width of the 95% confidence interval, *this sample size having 80% power to reject null hypothesis at a 30% response*). f) The randomised sample of 375 patients will provide 80% power to detect a difference of at least 6% or larger in the 12-month overall trial retention rates from a hypothesised 75% retention, based on two-sided one-sample test of proportion at 5% significance level (i.e. providing statistical evidence that the overall retention may be as low or high as 69% (0.69) or 81% (0.81) respectively, as opposed to 75%, 0.75, = *Sample size for 80% power to reject null hypothesis of 75% retention*), g) Power vs sample size to detect differences in 12-month treatment rates in our 3-arm trial, assuming treatment percentages of 4.5% vs. 15.5% vs. 20.3% with 1:1:1 randomisation to Usual Care vs. Active Control FRAX-GP vs. PHOENIX Intervention respectively, and type 1 error = 0.05

For peer review only - http://bmjopen.bmj.com/site/about/guidelines.xhtml

Authorship:

All authors agree to be accountable for all aspects of the work to enable questions relating to the accuracy or integrity of any part of the work to be appropriately investigated and resolved

Contributorship statement:

KESP Chief Investigator for the study and is responsible for the concept and design of the overall study and manuscript final approval.

KESP and EMC provided clinical expertise in the area of bone health which forms the basis for the study. They developed the concept and idea for PHOENIX, gained funding and wrote the clinical decision tree with assistance from DDGC. Also methods design, writing and providing feedback of manuscript, manuscript final approval. KESP is responsible for the medical aspects of opportunistic diagnosis of osteoporosis, recommending investigations and treatments to participants.

SKK (with assistance from the relevant trials work of LS, and pilot work of KESP/DDGC) developed the statistical analysis plan including calculation of sample size and definition of primary and secondary outcomes and power calculations.

APW designed the health economics work and developed suitable outcomes for the trial.

DDGC provided information on the background and rationale for using the various software. He has helped clarify pathways for scan analysis in the study and assisted in the development of the Clinical Decision Tree.

KW wrote the protocol and drafted the manuscript, clarified pathways and study flow and liaised with the team to finalise the protocol.

JF designed the process evaluation sub study, with all qualitative interview work, and developed suitable outcomes for the trial.

TDT provided information on the background and rationale for diagnosing vertebral fractures. He was responsible for the medical aspects of opportunistic diagnosis of vertebral fractures.

Acknowledgements: The authors would like to acknowledge Karen Blesic for providing a valuable nursing perspective, for helping develop the idea and for her major contributions towards the NHS Research Innovation Fund pilot to PHOENIX (the clinical service called CORTEX). We acknowledge the contributions of Polly Barnes and Charlotte Tyson to CORTEX and also their contributions towards the practicalities of imaging data flow. We acknowledge Judith Brown's

For peer review only - http://bmjopen.bmj.com/site/about/guidelines.xhtml

contributions towards the practicalities of patient flow and analysis through the pathways and imaging services. We acknowledge Jeremy Dearling as our primary PPIE representative who gave valuable contributions to the design of the overall study, provided detailed feedback on the materials and protocol development. We also acknowledge the assistance of the Eastern region NIHR Research Design Service team, specifically Jonathan Scales, Andrew Sharpe, Analisa Casarin (improving and developing the research idea and application) and Doreen Tembo (PPI lead for the same). KESP acknowledges the Cambridge NIHR Biomedical Research Centre who support his work, and support DDGC. KESP also thanks the NIHR for funding his attendance at the Pragmatic Clinical Trials Course at Queen Mary University of London which helped him develop the idea. Finally the authors acknowledge the major contribution of Dr Alan Lamont, chair of Cambridge East, Research Ethic Committee whose helpful suggestions and understanding of the relevant GCP issues and legislation rekindled our interest in adopting pragmatic patient consent without a researcher present.

Competing interests statement: No competing interests declared.

Funding: This project is funded by the National Institute for Health Research (NIHR) under its Research for Patient Benefit (RfPB) Programme (Grant Reference Number PB-PG-0816-20027) and by the Cambridge NIHR Biomedical Research Centre (BRC-1215-20014). The views expressed are those of the author(s) and not necessarily those of the NIHR or the Department of Health and Social Care. Funding is in place to 31.03.2022.

Data sharing statement:

Researchers involved in the trial who will have access to the full dataset. Applications for access to the final trial dataset will be through the Trial Management Group. All members of the TMG will have full access to the dataset, and a controlled access model (openly available to all applicants) will be followed as set out in the MRC and NIHR guidance https://www.methodologyhubs.mrc.ac.uk/files/7114/3682/3831/Datasharingguidance2015.pdf

References

[1] Lee SJ, Binkley N, Lubner MG, et al. Opportunistic screening for osteoporosis using the sagittal reconstruction from routine abdominal CT for combined assessment of vertebral fractures and density. Osteoporosis International 2016;27(3):113, 1-36. doi:10.1007/s00198-0153318-

[2.]Carberry GA, Pooler BD, Binkley N, et al. Unreported Vertebral Body Compression Fractures at Abdominal Multidetector CT. Radiology 2013;268(1):120-26. doi:10.1148/radiol.13121632

[3] Al-Sari UA, Tobias J, Clark E. Health-related quality of life in older people with osteoporotic vertebral fractures: a systematic review and meta-analysis. Osteoporosis International 2016;27(10):2891-900. doi:10.1007/s00198-016-3648-x

[4] Department of Health. Hospital episode statistics (England) 2006.

For peer review only - http://bmjopen.bmj.com/site/about/guidelines.xhtml

[5] Jewell PD, Mitchell R, Javaid MK, et al. Reporting and Follow-up of Vertebral Fractures Seen on Imaging; Can Radiologists Help Reduce the Number of Hip Fractures? Osteoporosis International 2014;25:S674-S74.

[6] Harvey N, Cushnaghan J, Lawrence W, et al. Anxiety, Fracture Risk and Adherence to Treatment: The MRC SCOOP Trial. Osteoporosis International 2012;2 3:S531-S31.

[7] Steiger P, Staal J, Engelke K, et al. Validation of an Automatic Vertebral Prevalent Fracture Classifier Based Upon Full Vertebral Shape. Osteoporosis International 2013;24:S307-S08.

[8] Shepstone L, Lenaghan E, Cooper C, et al. Screening in the community to reduce fractures in older women (SCOOP) - a randomised controlled trial Lancet 2017(pii)S0140-6736(17)32640-5.

[9] OECD (2014). Computed tomography (CT) exams, total. Health: Key Tables from OECD, OECD - ISSN 2075-8480.

[10] National Institute for Health and Care Excellence. Osteoporosis: assessing the risk of fragility fracture 2012. NICE guidelines CG146, 2015

[11] Nelson HD, Haney EM, Dana T, et al. Screening for Osteoporosis: An Update for the US Preventive Services Task Force. Annals of internal medicine 2010;153(2):99-W43. doi:10.7326/0003-4819-153-2-201007200-00262

[12] Lindsay R et al. Risk of new vertebral fracture in the year following a fracture. JAMA. 2001 Jan 17;285(3):320-3

[13] https://www.england.nhs.uk/statistics/wp-content/uploads/sites/2/2016/08/Provisional-Monthly-Diagnostic-Imaging-Dataset-Statistics-2017-05-18.pdf

[14] Pickhardt PJ, Pooler BD, Lauder T, et al. Opportunistic screening for osteoporosis using abdominal computed tomography scans obtained for other indications. Annals of internal medicine 2013;158(8):588-95. doi:10.7326/0003-4819-158-8-201304160-00003

[15] Ritchie J, Spencer L. Qualitative data analysis for applied policy research. In: Bryman A, Burgess R, editors. Analyzing qualitative data. London: Routledge; 1994. p. 173–94.

[16] Moore G, Audrey S, Barker M, Bond L, Bonell C, Hardeman W, Moore L, O'Cathain A, Tinati T, Wight D, Baird J. Process evaluation of complex interventions: Medical Research Council guidance. MRC Population Health Science Research Network, London, 2014

[17] Moore GF, Audrey S, Barker M, Bond L, Bonell C, Hardeman W, Moore L, O'Cathain A, Tinati T, Wight D, Baird J. Process evaluation of complex interventions: Medical Research Council guidance. BMJ 2015;350:h1258 doi:10.1136/bmj.h1258

For peer review only - http://bmjopen.bmj.com/site/about/guidelines.xhtml

Issue Date: 06.10.2020

Protocol Version No: 2.0

Protocol substantial amendment number: 02

Revision chronology:

Protocol v1.2 Original

Protocol v1.3 (amendment 1)

Protocol v2.0 (amendment 2)

Primary reason for Substantial Amendment 1: To allow participants to self-consent to the study during the Covid-19 pandemic. Increase in number of recruits permitted to be obtained from one site (Cambridge) as other sites paused non-Covid research. Additional changes: To allow review of scans when home working using a secure Information Technology (IT) environment

Substantial Amendment 2: Change to one of the primary outcomes related to the study retention at one year to take account of the relatively high mortality in this cohort. The original outcome stated that the study would be considered feasible if treated / not on treatment outcome was determined in ≥75% of the sample at 12 months from randomisation (S3.4.Phoenix_protocol v1.2_2019_07_01). This was amended to ≥75% of those **surviving** at 12 months post randomisation. This change was based on: i) a likely increased disease severity among patients who continued to have CT scans during the first wave of Covid-19 (many non-cancer scans were cancelled, skewing the study sample to those with cancer monitoring); and ii) findings from our own VORTEX Study (Vertebral fracture and Osteoporosis screening from Routine CT as an added EXtra – the VORTEX Study).

Trial Sponsor: Cambridge University NHS Foundation Trust & University of Cambridge

For peer review only - http://bmjopen.bmj.com/site/about/guidelines.xhtml

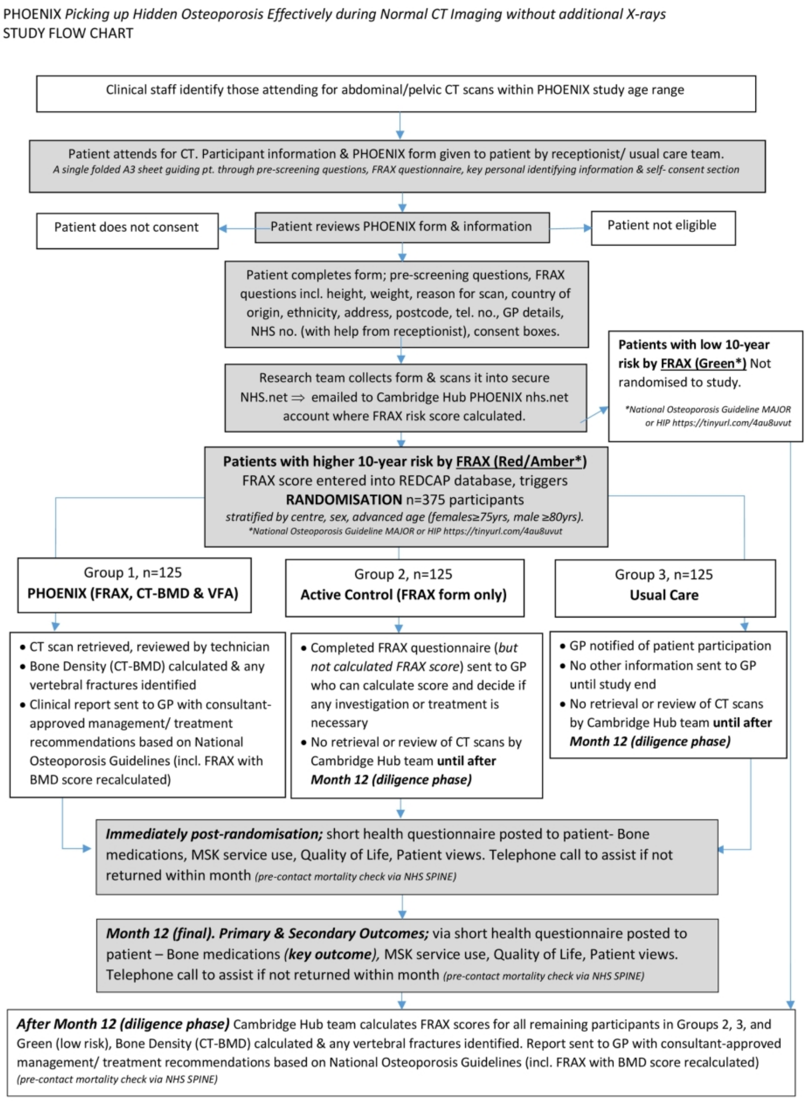

Figure 1. PHOENIX Study flow chart

210x297mm (300 x 300 DPI)

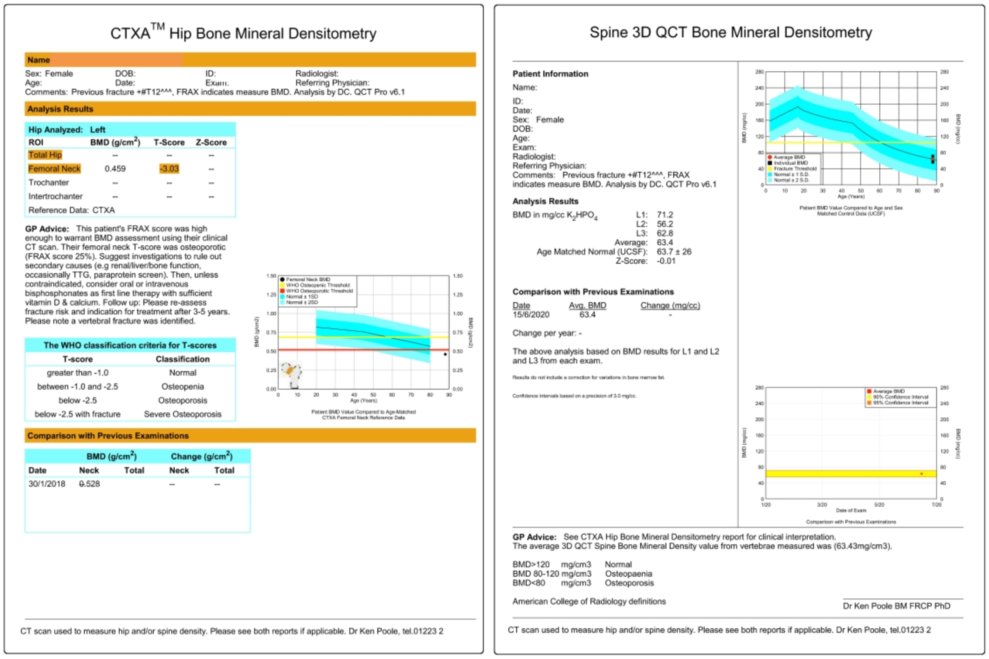

Figure 2. Written clinical guidance and report produced at the end of a PHOENIX Intervention in a typical patient from Group 1. These comprise the standard output pdf's of Mindways QCT Pro which are sent to the participants' GP. In this case there are both hip and spine results from a typical patient with a previously undiagnosed grade 3 vertebral fracture of T12 (found and graded using SlicePick). The femoral neck BMD T-score is -3.03 and spinal osteoporosis is shown with L1-L3 volumetric BMD of 63.4mg/cm3. Their FRAX 10-year risk of fracture is 25% and investigation/treatment is recommended by NOGG. The written clinical guidance is derived from the PHOENIX clinical decision tree (Supplemental file 2)

419x296mm (300 x 300 DPI)

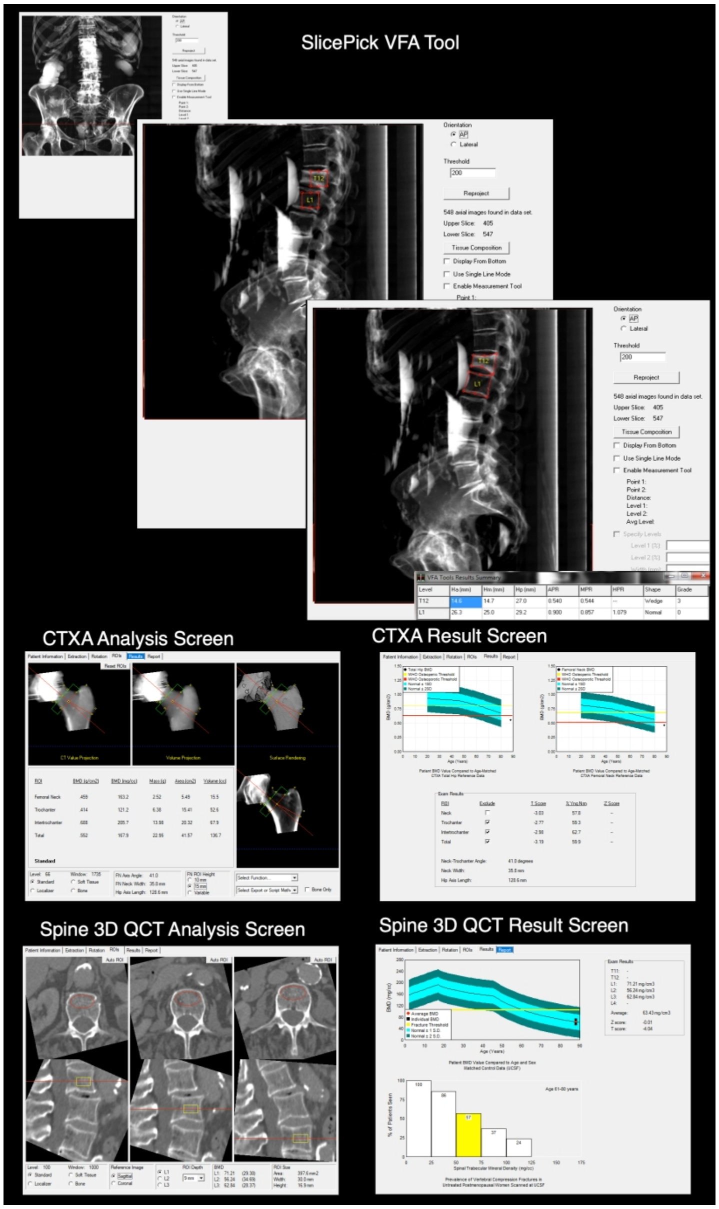

Figure 3. The PHOENIX Intervention: technical steps involved in Vertebral Fracture Assessment using Mindways SlicePick to automatically create the sagittal synchronised image in a patient where the technician has used embedded 6-point morphometry to grade a previously undiagnosed T12 vertebral fracture (upper panels). Note the radiopaque bowel contrast in AP and lateral SlicePick views. SlicePick is then used to create a volume for femoral neck (CTXATM) BMD and spine volumetric BMD measurements from the same CT scan. The middle panels show screenshots from CTXATM femoral neck areal BMD (g/cm2) analysis from CliniQCT. Note that in this case, only the femoral neck BMD value is taken forwards to the report/guidance (Figure 2), since the CT scan did not cover sufficiently far below the lesser trochanter to allow for 'total hip' BMD measurement. The lower panels show 3D QCT analysis of Lumbar spine L1, L2 and L3 trabecular volumetric BMD (mg/cm3). Note that Lumbar Spine volumetric BMD T-scores are not used in the written clinical guidance and report shown in Figure 2, since trabecular bone spinal vBMD T-scores are not interchangeable with DXA-derived T-scores.

151x254mm (300 x 300 DPI)

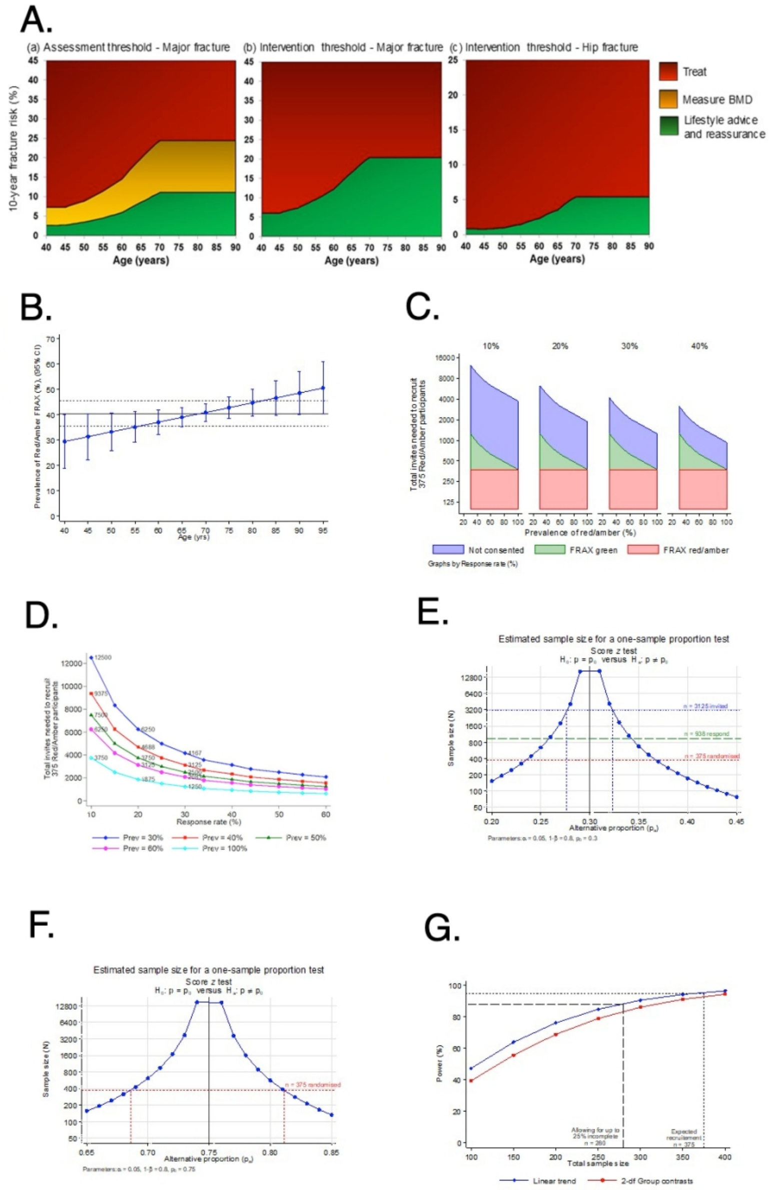

Figure 4. FRAX, sample size and power calculations for PHOENIX a) FRAX UK age-specific fracture risk assessment and intervention thresholds, b) Age-specific prevalence of red/amber FRAX risk category from preliminary local application of PHOENIX intervention in Cambridge, c) Calculation of the number of invites needed to randomise 375 red/amber risk participants based on different responses (rates of agreeing to participate), d) Number of PHOENIX invitation packs that must be given out in order to randomise 375 red/amber risk participants based on different prevalence of red/amber FRAX among participants, e) Our feasibility study aiming to randomise 375 of 938 (40%) consenting participants (i.e. 30% of 3125 total invited) would estimate the trial's response rate with a precision of 1.6% (i.e. half-width of the 95% confidence interval, this sample size having 80% power to reject null hypothesis at a 30% response). f) The randomised sample of 375 patients will provide 80% power to detect a difference of at least 6% or larger in the 12-month overall trial retention rates from a hypothesised 75% retention, based on two- sided one-sample test of proportion at 5% significance level (i.e. providing statistical evidence that the overall retention may be as low or high as 69% (0.69) or 81% (0.81) respectively, as opposed to 75%, 0.75, = Sample size for 80% power to reject null hypothesis of 75% retention), g) Power vs sample size to detect

differences in 12-month treatment rates in our 3-arm trial, assuming treatment percentages of 4.5% vs. 15.5% vs. 20.3% with 1:1:1 randomisation to Usual Care vs. Active Control FRAX-GP vs. PHOENIX Intervention respectively, and type 1 error = 0.05

84x132mm (300 x 300 DPI)

Appendix 1

Osteoporosis is a condition that makes bones become fragile and break more easily, especially in the spine and hips. Osteoporosis can be treated. We would like to diagnose it earlier and are running a study called PHOENIX-f to see if this can be done using the scan that you are having today. There are no extra scans, other tests or treatment required to join the study.

If you are interested in being part of the study, please turn over the page and read on

If you do not wish to take part in the study, can you please tell us why in the box below? This would be really useful for us to know, but you do not have to tell us.

Now please return this form to the PHOENIX-f returns box

Thank you for your co-operation

For peer review only

For peer review only - http://bmjopen.bmj.com/site/about/guidelines.xhtml

By completing this form, we understand that you are happy for one of our research team to talk to you about the study.

Your scan taken today can also be used to measure your bone health.

We can't use every scan, so please answer the following 3 questions to see if we can use yours.

Please circle your answer

**Do you take medication for your bones once a week that require you to sit or stand upright?**	**Y**	**N**
**Do you have a yearly infusion (drip) or injection for your bones?**	**Y**	**N**
**Do you have a hip replacement? Have you ever had a hip operation?**	**Y**	**N**

If you answered 'Yes' to any of the questions above unfortunately we will be unable to use your CT scan today for PHOENIX-f.

Please return this form to the PHOENIX-f returns box.
Thank you for your co-operation.

If you have answered 'NO' to ALL the questions above we will be able to review your CT scan for PHOENIX-f.

In order to understand the study more, please read the patient information leaflet. Then, if you are interested in taking part in PHOENIX to measure your bone health, please fill in the questionnaire overleaf and complete the consent form

For peer review only

For peer review only - http://bmjopen.bmj.com/site/about/guidelines.xhtml

PHOENIX-f Consent Form

Chief Investigator: Dr Ken Poole, Consultant in Rheumatology and Metabolic Bone Disease, Cambridge

Please *initial* boxes below, then sign and date the form and add your address ***Initials***

1. I confirm that I have read and understood the patient information for the study. ☐
2. I understand that my participation is voluntary and that I am free to withdraw at any time without giving any reason, without my medical care or legal rights being affected. ☐
3. If I decide to withdraw from the trial I agree to data already collected to be used for Phoenix-f. ☐
4. I give permission for the study researchers to have access to my scan and relevant NHS databases in order to conduct this research ☐
5. I agree for my personal details and relevant medical information to be sent from my hospital to the study team at Addenbrooke's Hospital for the purpose of the research. I understand that scans have identifiable data and will be kept securely at Addenbrooke's Hospital and will only be accessed by the research team ☐
6. I agree to the research team contacting my GP to notify them of my participation in the study and any results ☐
7. I give permission to be contacted by post or phone by the study team at Addenbrooke's Hospital. I understand that this will be to send me 2 questionnaires and may include an invitation to talk to a researcher about my experience of the study ☐
8. I agree to take part in the above study. ☐

Mr / Mrs / Ms / Dr (please circle as appropriate)

....

Please enter first and first family name above

……………………………………………………………. *Date:* ……………………………

Your Signature

Date of Birth: .... *Height:* .... *Weight:* ....

NHS number: ....

Why are you having a scan today? ....

The following questions are required for accurate bone health calculation:

Your country of origin: .... *Ethnicity:* ....

PLEASE TURN OVER PAGE TO COMPLETE THIS FORM

For peer review only - http://bmjopen.bmj.com/site/about/guidelines.xhtml
For peer review only

4

BONE HEALTH QUESTIONNAIRE	Yes	No	Not Sure
Have you broken any bones since you turned 40?			
If Yes, which bones?			
Did your mother or father ever break a hip?			
Do you currently smoke?			
Do you take steroid tablets now (eg: 'prednisolone'), or have you taken steroid tablets in the past, for longer than three months at any time?			
Have you been diagnosed with rheumatoid arthritis?			
Have you been diagnosed with any of the following?<br>**(Tick 'yes' if any of the following apply)**<br>• Premature menopause (before 45yrs of age)<br>• Insulin dependent diabetes<br>• Brittle bone disease, Ankylosing spondylitis<br>• Overactive thyroid<br>• Low testosterone level (men only)<br>• Chronic malnutrition or malabsorption eg: anorexia or coeliac disease<br>• Chronic liver disease			
Do you drink three or more units of alcohol a **day**, on average? (That's 1 1/2 pints of beer or 3 glasses of wine or 3 short measures of spirits)			

Your Address (for questionnaires):

....

....

....

....

Post Code: .... **Your Telephone no:** ....

Your GP Name and address:

....

....

Now please return this form to the PHOENIX-f returns box. Thank you for your co-operation

PHOENIX Methodology paper_2021_04_29_ submission 4

For peer review only - http://bmjopen.bmj.com/site/about/guidelines.xhtml

PHOENIX-f Clinical Decision Tree *part 1*

Initial steps

1. Retrieve and visually inspect CT images for any vertebral fractures with AP and lateral localiser views (using Mindways QCT PRO SlicePick for 6-point morphometry).

2. Measure CTXA Hip and/or Spine BMD with QCT PRO, noting Femoral neck & Hip BMD results (in g/cm2) and/or Average L1-L3 Spine BMD results (in mg/cm3).

3. Recalculate FRAX online using the Mindways Femoral neck/Hip BMD (g/cm2) value & change 'Previous Fracture' to 'Yes' if a Grade 2 or 3 fracture is found at step 1.

4. If only one (Hip or Spine) result is available, follow the GP Advice Clinical Decision Tree here to generate automatic GP Advice text.

5. If both Hip BMD and Spine BMD results are available, CTXA Femoral neck/Hip results usually dominate, and the following generic Spine 3D QCT GP advice box text is automatically added, "See CTXA Hip Bone Mineral Densitometry report for clinical interpretation." In GP Advice box add the Spine BMD value and definitions of Normal/ Osteopaenia/Osteoporosis.

6. Only if Spine BMD value is <80mg/cm3 AND NOGG advice is GREEN (after entering Femoral neck/Hip BMD into FRAX) does a Spine result dominate. In this case, the following generic CTXA Hip GP advice box text is automatically added, "Both hip and spine results are available. For this patients' clinical interpretation, please see their associated Spine 3D QCT BMD result".

GP advice text using *Mindways QCT Pro Spine L1-3 BMD value + initial FRAX score/NOGG*

ACR Spine BMD Thresholds

**Abnormal High** BMD >200mg/cm^3	**Normal** BMD >120mg/cm^3		**Osteopenia** 120mg/cm^3 ≥ BMD > 80mg/cm^3		**Osteoporosis** BMD ≤ 80mg/cm^3
	**Initial FRAX Major osteoporotic fracture risk ≤ 20%**	**Initial FRAX Major osteoporotic fracture risk >20%**	**Initial FRAX Major osteoporotic fracture risk ≤ 20%**	**Initial FRAX Major osteoporotic fracture risk >20%**	
This patient had a FRAX score high enough to warrant a BMD assessment which was done using the clinical CT scan. Their BMD of (***) mg/cm3 (*) is higher than expected. High density can be due to degenerative/ genetic effects or for other reasons.	This patient had a FRAX score high enough to warrant a BMD assessment which was done using the clinical CT scan. Their spine BMD >120mg/cm3 (*) is defined as normal bone density by ACR criteria. Unless there is a change in the clinical circumstances, no routine repeat BMD tests are indicated.	This patient had a FRAX score high enough to warrant BMD assessment using the clinical CT scan. Their spine BMD >120mg/cm3 (*) which is defined as normal bone density by ACR criteria. However, there are clinical risk factors which elevate the 10 year future fracture risk (**%).	This patient had a FRAX score high enough to warrant a BMD assessment which was done using the clinical CT scan. Their SPINE BMD of 80-120 mg/cm3 (*) is defined as osteopenia by ACR criteria. There is no current indication for bone active therapy, but the patient may wish to follow the lifestyle advice to maintain bone density (if relevant to patient avoid smoking and excess alcohol intake, and maintain weightbearing exercise.) Please use the online WHO FRAX tool to determine if a standard DXA scan is needed in 3 years.	This patient had a FRAX score high enough to warrant BMD assessment using the clinical CT scan. Their SPINE BMD of 80-120 mg/cm3(*) is defined as osteopenia by ACR criteria. However, there are clinical risk factors which elevate the 10 year future fracture risk above the treatment threshold (**%). Unless contraindicated, consider bisphosphonate treatment.	This patient had a FRAX score high enough (10 year future risk **%) to warrant a BMD assessment using their clinical CT scan. Their SPINE BMD of <80mg/cm3 (*) is defined as osteoporosis by ACR criteria. We suggest investigations to rule out secondary causes (e.g. renal/liver/bone function, low vit D, high PTH, occasionally TTG, paraprotein screen). Then, unless contraindicated, consider oral or intravenous bisphosphonates as first line therapy with sufficient vitamin D & calcium. If relevant to patient, please recommend avoid smoking, excess alcohol intake, & maintain weightbearing exercise. Follow up: Please re-assess fracture risk and indication for treatment after 3-5 years
If grade 2 or 3 vertebral # identified.<br>Include:<br>Please note that a vertebral fracture was identified. Unless this was traumatic/already known, you may wish to investigate further	If grade 2 or 3 vertebral # identified.<br>Include:<br>Please note that a vertebral fracture was identified. Unless this was traumatic/already known, you may wish to investigate further	If grade 2 or 3 vertebral # identified.<br>Include:<br>Please note that a vertebral fracture was identified. Unless this was traumatic/already known, you may wish to investigate further	If grade 2 or 3 vertebral # identified.<br>Include:<br>Please note that a vertebral fracture was identified. Unless this was traumatic/already known, you may wish to investigate further	If grade 2 or 3 vertebral # identified.<br>Include:<br>Please note that a vertebral fracture was identified. Unless this was traumatic/already known, you may wish to investigate further	If grade 2 or 3 vertebral # identified.<br>Include:<br>Please note that a vertebral fracture was identified. Unless this was traumatic/already known, you may wish to investigate further

* Vertebrae analysed

** Major Fracture Risk Score

*** Average BMD value of vertebrae analysed

American College of Radiology

PHOENIX-f Clinical Decision Tree *part 2*

GP advice text using *Mindways QCT Pro CTXA Femoral neck BMD value + BMD-Adjusted FRAX/NOGG*

WHO BMD T-Score Criteria								
**Abnormal High**<br>**T-Score > 2**	**Normal**<br>**T-Score > -1**			**Osteopenia**<br>**-1 ≥ T-Score > -2.5**			**Osteoporosis**<br>**T-Score ≤ -2.5**	
**Any FRAX/NOGG Threshold**	**Below FRAX /NOGG Treatment Threshold**	**Below FRAX/NOGG Treatment Threshold AND ≥75yrs with prior fragility#/vert #**	**Above FRAX/NOGG Treatment Threshold**	**Below FRAX/NOGG Treatment Threshold**	**Either i) Below FRAX/NOGG Treatment Threshold ≥75yrs with prior fragility#/vert# OR ii) Between -2.0 & -2.50 with prior fragility#/vert#**	**Above FRAX/NOGG Treatment Threshold**	**Below FRAX/NOGG Treatment Threshold**	**Either i) Above FRAX/NOGG Treatment Threshold OR ii) Below treatment threshold with a prior fracture aged ≥75**
This patient has a FRAX score high enough to warrant a BMD assessment which was done using their clinical CT scan. Their *femoral neck/ total hip T-score was (***) which is higher than expected which can have genetic, degenerative or other causes. There is no evidence of osteoporosis	This patient had a FRAX score high enough to warrant a BMD assessment using the clinical CT scan. Their *femoral neck/total hip T-score was >-1 which is defined as normal hip bone density by WHO guidelines (FRAX score **%). Unless there is a change in the clinical circumstances, no routine repeat BMD tests are indicated.	This patient had a FRAX score high enough to warrant a BMD assessment using the clinical CT scan. Their *femoral neck/ total hip T-score was >-1 which is defined as normal hip bone density by WHO guidelines (FRAX score **%). Please note National Guidelines recommend bisphosphonate treatment in the majority of elderly people with a prior fragility fracture regardless of calculated risk. If relevant to patient, recommend avoid smoking, excess alcohol intake, & maintain weightbearing exercise.	This patient had a FRAX score high enough to warrant a BMD assessment using the clinical CT scan. Their *femoral neck/total hip T-score was >-1 which is defined as normal hip bone density by WHO guidelines. However, there are clinical risk factors which elevate the 10 year future fracture risk (FRAX score **%).	This patient had a FRAX score high enough to warrant BMD assessment using the clinical CT scan. Their *femoral neck/ total hip T-score was between -1 and -2.5 which is defined as osteopenia, or low normal bone density, by WHO guidelines. (FRAX score **%). There is no current indication for bone active therapy, but the patient may wish to follow the lifestyle advice to maintain bone density (if relevant to patient avoid smoking and excess alcohol intake, and maintain weightbearing exercise.) Please use the online WHO FRAX tool to determine if a standard DXA scan is needed in 3 years.	This patient had a FRAX score high enough to warrant BMD assessment using the clinical CT scan. Their *femoral neck/total hip T-score was between -1 and > -2.5 which is defined as osteopenia, or low normal bone density, by WHO guidelines (FRAX Score **%). Please note National Guidelines recommend bisphosphonate treatment in the majority of older people with a prior fragility fracture regardless of calculated risk. Unless contraindicated consider bisphosphonate treatment. If relevant to patient, recommend avoid smoking, excess alcohol intake, & maintain weightbearing exercise.	This patient had a FRAX score high enough to warrant BMD assessment using the clinical CT scan. Their *femoral neck/total hip T-score was between -1 and > -2.5 which is defined as osteopenia, or low normal bone density, by WHO guidelines. However, there are clinical risk factors which elevate the 10 year future fracture risk above the treatment threshold (FRAX Score **%). Unless contraindicated consider bisphosphonate treatment. If relevant to patient, recommend avoid smoking, excess alcohol intake, & maintain weightbearing exercise.	This patient's FRAX score was high enough to warrant BMD assessment using their clinical CT scan. Their *femoral neck/total hip T-score was osteoporotic (FRAX score **%). While the scan indicates osteoporosis at the hip, treatment is not automatically recommended (by National Guidelines) at this level of 10-year risk. Suggest investigations to rule out secondary causes (e.g renal/liver/bone function, occasionally TTG, paraprotein screen) The patient may wish to follow the lifestyle advice to maintain bone density (if relevant to patient avoid smoking and excess alcohol intake, and maintain weightbearing exercise.)	This patient's FRAX score was high enough to warrant BMD assessment using their clinical CT scan. Their *femoral neck/ total hip T-score was osteoporotic (FRAX score **%). Suggest investigations to rule out secondary causes (e.g renal/liver/ bone function, occasionally TTG, paraprotein screen). Then, unless contraindicated, consider oral or intravenous bisphosphonates as first line therapy with sufficient vitamin D & calcium. Follow up: Please re-assess fracture risk and indication for treatment after 3-5 years
If grade 2 or 3 vertebral # identified<br>Include:<br>Please note that a vertebral fracture was identified, unless this was traumatic/already known, you may wish to investigate further	If grade 2 or 3 vertebral # identified, <75y/o only.<br>Include:<br>Please note that a vertebral fracture was identified, unless this was traumatic/ already known, you may wish to order a spine only DXA scan.	If grade 2 or 3 vertebral # identified.<br>Include:<br>Please note that a vertebral fracture was identified.	If grade 2 or 3 vertebral # identified, <75y/o only.<br>Include:<br>Please note that a vertebral fracture was identified, unless this was traumatic/ already known, you may wish to order a spine only DXA scan."	If grade 2 or 3 vertebral # identified, <75y/o only.<br>Include:<br>Please note that a vertebral fracture was identified, unless this was traumatic/already known, you may wish to order a spine only DXA scan.	If grade 2 or 3 vertebral # identified<br>Include:<br>Please note that a vertebral fracture was identified.	If grade 2 or 3 vertebral # identified<br>Include:<br>Please note that a vertebral fracture was identified.	If grade 2 or 3 vertebral # identified, <75y/o only<br>Include:<br>Please note that a vertebral fracture was identified.	If grade 2 or 3 vertebral # identified<br>Include:<br>Please note that a vertebral fracture was identified.

* Where lowest BMD T-Score was at either the Femoral Neck or Total Hip

** Value of either the Major FRAX score OR the Hip Fracture FRAX score when this value is above the FRAX/NOGG treatment threshold AND Major is below the threshold.

*** Value of the BMD T-Score

Reporting checklist for protocol of a clinical trial.

Based on the SPIRIT guidelines.

Instructions to authors

Complete this checklist by entering the page numbers from your manuscript where readers will find each of the items listed below.

Your article may not currently address all the items on the checklist. Please modify your text to include the missing information. If you are certain that an item does not apply, please write "n/a" and provide a short explanation.

Upload your completed checklist as an extra file when you submit to a journal.

In your methods section, say that you used the SPIRITreporting guidelines, and cite them as:

Chan A-W, Tetzlaff JM, Gøtzsche PC, Altman DG, Mann H, Berlin J, Dickersin K, Hróbjartsson A, Schulz KF, Parulekar WR, Krleža-Jerić K, Laupacis A, Moher D. SPIRIT 2013 Explanation and Elaboration: Guidance for protocols of clinical trials. BMJ. 2013;346:e7586

For peer review only

		Reporting Item	Page Number
**Administrative information**			
Title	#1	Descriptive title identifying the study design, population, interventions, and, if applicable, trial acronym	1

Trial registration	#2a	Trial identifier and registry name. If not yet registered, name of intended registry	1
Trial registration: data set	#2b	All items from the World Health Organization Trial Registration Data Set	1
Protocol version	#3	Date and version identifier	1
Funding	#4	Sources and types of financial, material, and other support	10
Roles and responsibilities: contributorship	#5a	Names, affiliations, and roles of protocol contributors	2
Roles and responsibilities: sponsor contact information	#5b	Name and contact information for the trial sponsor	2
Roles and responsibilities: sponsor and funder	#5c	Role of study sponsor and funders, if any, in study design; collection, management, analysis, and interpretation of data; writing of the report; and the decision to submit the report for publication, including whether they will have ultimate authority over any of these activities	2
Roles and responsibilities: committees	#5d	Composition, roles, and responsibilities of the coordinating centre, steering committee, endpoint adjudication committee, data management team, and	5

For peer review only

		other individuals or groups overseeing the trial, if applicable (see Item 21a for data monitoring committee)	
**Introduction**			
Background and rationale	#6a	Description of research question and justification for undertaking the trial, including summary of relevant studies (published and unpublished) examining benefits and harms for each intervention	3
Background and rationale: choice of comparators	#6b	Explanation for choice of comparators	5
Objectives	#7	Specific objectives or hypotheses	3
Trial design	#8	Description of trial design including type of trial (eg, parallel group, crossover, factorial, single group), allocation ratio, and framework (eg, superiority, equivalence, non-inferiority, exploratory)	4
**Methods: Participants, interventions, and outcomes**			
Study setting	#9	Description of study settings (eg, community clinic, academic hospital) and list of countries where data will be collected. Reference to where list of study sites can be obtained	3

For peer review only

Eligibility criteria	#10	Inclusion and exclusion criteria for participants. If applicable, eligibility criteria for study centres and individuals who will perform the interventions (eg, surgeons, psychotherapists)	7
Interventions: description	#11a	Interventions for each group with sufficient detail to allow replication, including how and when they will be administered	5
Interventions: modifications	#11b	Criteria for discontinuing or modifying allocated interventions for a given trial participant (eg, drug dose change in response to harms, participant request, or improving / worsening disease)	n/a
Interventions: adherance	#11c	Strategies to improve adherence to intervention protocols, and any procedures for monitoring adherence (eg, drug tablet return; laboratory tests)	n/a
Interventions: concomitant care	#11d	Relevant concomitant care and interventions that are permitted or prohibited during the trial	7
Outcomes	#12	Primary, secondary, and other outcomes, including the specific measurement variable (eg, systolic blood pressure), analysis metric (eg, change from baseline, final value, time to event), method of aggregation (eg, median, proportion), and time point for each outcome. Explanation of the clinical relevance of chosen efficacy and harm outcomes is strongly recommended	7

For peer review only

Participant timeline	#13	Time schedule of enrolment, interventions (including any run-ins and washouts), assessments, and visits for participants. A schematic diagram is highly recommended (see Figure)	n/a
Sample size	#14	Estimated number of participants needed to achieve study objectives and how it was determined, including clinical and statistical assumptions supporting any sample size calculations	7
Recruitment	#15	Strategies for achieving adequate participant enrolment to reach target sample size	n/a
**Methods: Assignment of interventions (for controlled trials)**			
Allocation: sequence generation	#16a	Method of generating the allocation sequence (eg, computer-generated random numbers), and list of any factors for stratification. To reduce predictability of a random sequence, details of any planned restriction (eg, blocking) should be provided in a separate document that is unavailable to those who enrol participants or assign interventions	9
Allocation concealment mechanism	#16b	Mechanism of implementing the allocation sequence (eg, central telephone; sequentially numbered, opaque,	n/a

For peer review only

		sealed envelopes), describing any steps to conceal the sequence until interventions are assigned	
Allocation: implementation	#16c	Who will generate the allocation sequence, who will enrol participants, and who will assign participants to interventions	9
Blinding (masking)	#17a	Who will be blinded after assignment to interventions (eg, trial participants, care providers, outcome assessors, data analysts), and how	n/a
Blinding (masking): emergency unblinding	#17b	If blinded, circumstances under which unblinding is permissible, and procedure for revealing a participant's allocated intervention during the trial	n/a
**Methods: Data collection, management, and analysis**			
Data collection plan	#18a	Plans for assessment and collection of outcome, baseline, and other trial data, including any related processes to promote data quality (eg, duplicate measurements, training of assessors) and a description of study instruments (eg, questionnaires, laboratory tests) along with their reliability and validity, if known. Reference to where data collection forms can be found, if not in the protocol	9

For peer review only

Data collection plan: retention	#18b	Plans to promote participant retention and complete follow-up, including list of any outcome data to be collected for participants who discontinue or deviate from intervention protocols	n/a
Data management	#19	Plans for data entry, coding, security, and storage, including any related processes to promote data quality (eg, double data entry; range checks for data values). Reference to where details of data management procedures can be found, if not in the protocol	n/a
Statistics: outcomes	#20a	Statistical methods for analysing primary and secondary outcomes. Reference to where other details of the statistical analysis plan can be found, if not in the protocol	9
Statistics: additional analyses	#20b	Methods for any additional analyses (eg, subgroup and adjusted analyses)	9
Statistics: analysis population and missing data	#20c	Definition of analysis population relating to protocol non-adherence (eg, as randomised analysis), and any statistical methods to handle missing data (eg, multiple imputation)	9

Methods: Monitoring

Data monitoring: formal committee	#21a	Composition of data monitoring committee (DMC); summary of its role and reporting structure; statement of whether it is independent from the sponsor and competing interests; and reference to where further	n/a

For peer review only

		details about its charter can be found, if not in the protocol. Alternatively, an explanation of why a DMC is not needed	
Data monitoring: interim analysis	#21b	Description of any interim analyses and stopping guidelines, including who will have access to these interim results and make the final decision to terminate the trial	n/a
Harms	#22	Plans for collecting, assessing, reporting, and managing solicited and spontaneously reported adverse events and other unintended effects of trial interventions or trial conduct	n/a
Auditing	#23	Frequency and procedures for auditing trial conduct, if any, and whether the process will be independent from investigators and the sponsor	n/a
**Ethics and dissemination**			
Research ethics approval	#24	Plans for seeking research ethics committee / institutional review board (REC / IRB) approval	10
Protocol amendments	#25	Plans for communicating important protocol modifications (eg, changes to eligibility criteria, outcomes, analyses) to relevant parties (eg, investigators, REC / IRBs, trial participants, trial registries, journals, regulators)	1

For peer review only

Consent or assent	#26a	Who will obtain informed consent or assent from potential trial participants or authorised surrogates, and how (see Item 32)	5
Consent or assent: ancillary studies	#26b	Additional consent provisions for collection and use of participant data and biological specimens in ancillary studies, if applicable	n/a
Confidentiality	#27	How personal information about potential and enrolled participants will be collected, shared, and maintained in order to protect confidentiality before, during, and after the trial	5
Declaration of interests	#28	Financial and other competing interests for principal investigators for the overall trial and each study site	10
Data access	#29	Statement of who will have access to the final trial dataset, and disclosure of contractual agreements that limit such access for investigators	10
Ancillary and post trial care	#30	Provisions, if any, for ancillary and post-trial care, and for compensation to those who suffer harm from trial participation	n/a
Dissemination policy: trial results	#31a	Plans for investigators and sponsor to communicate trial results to participants, healthcare professionals, the public, and other relevant groups (eg, via publication, reporting in results databases, or other data sharing arrangements), including any publication restrictions	10

For peer review only

Dissemination policy: authorship	#31b	Authorship eligibility guidelines and any intended use of professional writers	n/a
Dissemination policy: reproducible research	#31c	Plans, if any, for granting public access to the full protocol, participant-level dataset, and statistical code	10

Appendices

Informed consent materials	#32	Model consent form and other related documentation given to participants and authorised surrogates	12
Biological specimens	#33	Plans for collection, laboratory evaluation, and storage of biological specimens for genetic or molecular analysis in the current trial and for future use in ancillary studies, if applicable	n/a

The SPIRIT Explanation and Elaboration paper is distributed under the terms of the Creative Commons Attribution License CC-BY-NC. This checklist was completed on 16. February 2021 using https://www.goodreports.org/, a tool made by the EQUATOR Network in collaboration with Penelope.ai

For peer review only

BMJ Open

PHOENIX (Picking up Hidden Osteoporosis Effectively during Normal CT Imaging without additional X-rays). Protocol for a randomised, multi-centre feasibility study

Journal:	*BMJ Open*
Manuscript ID	bmjopen-2021-050343.R2
Article Type:	Protocol
Date Submitted by the Author:	09-Mar-2022
Complete List of Authors:	Poole, Kenneth; University of Cambridge School of Clinical Medicine; NIHR Cambridge Biomedical Research Centre<br>Chappell, Daniel; Cambridge University Hospitals NHS Foundation Trust, Rheumatology; NIHR Cambridge Biomedical Research Centre<br>Clark, Emma; University of Bristol, Clinical Science at North Bristol<br>Fleming, Jane; University of Cambridge, Cambridge Institute of Public Health; University of Cambridge, Department of Public Health and Primary Care<br>Shepstone, Lee; University of East Anglia<br>Turmezei, Tom; Cambridge University Hospitals NHS Foundation Trust, Radiology; Norfolk and Norwich University Hospital NHS Trust, Radiology<br>Wagner, Adam; NIHR Collaboration for Leadership in Applied Health Research & Care (CLAHRC) East of England; University of East Anglia, Norwich Medical School<br>willoughby, karen; University of Cambridge School of Clinical Medicine, Medicine; NIHR Cambridge Biomedical Research Centre<br>Kaptoge, Stephen; University of Cambridge
**Primary Subject Heading**:	Radiology and imaging
Secondary Subject Heading:	Rheumatology, General practice / Family practice
Keywords:	Bone diseases < ORTHOPAEDIC & TRAUMA SURGERY, Spine < ORTHOPAEDIC & TRAUMA SURGERY, Computed tomography < RADIOLOGY & IMAGING, Diagnostic radiology < RADIOLOGY & IMAGING, RHEUMATOLOGY, Back pain < ORTHOPAEDIC & TRAUMA SURGERY

SCHOLARONE™
Manuscripts

PHOENIX (Picking up Hidden Osteoporosis Effectively during Normal CT Imaging without additional X-rays). Protocol for a randomised, multi-centre feasibility study

Authors Kenneth E. S. Poole* (a, b), Daniel DG Chappell (a), Emma M Clark (c), Jane Fleming (b), Lee Shepstone (d), Thomas D Turmezei (a, e) Adam P Wagner (d, f), Karen Willoughby (b), Dr Stephen K Kaptoge (b).
***Corresponding**
(a) Cambridge University Hospitals NHS Trust, (b) University of Cambridge, (c) University of Bristol, (d) University of East Anglia, (e) Norfolk and Norwich University Hospitals NHS Trust, Norwich, (f) National Institute of Health Research (NIHR), Applied Research Collaboration (ARC) East of England (EoE),

Address: Dept of Medicine, University of Cambridge, Level 7, Hills Rd, Cambridge CB2 0QQ **Tel:** 01223 245151 **Email:** kenneth.poole@nhs.net

Key words: Osteoporosis; osteoporotic fracture reduction; opportunistic screening; vertebral fracture.

Introduction: Two million out of the UK's 5 million routine diagnostic computed tomography (CT) scans performed each year incorporate the thoraco-lumbar spine or pelvic region. Up to one third reveal undiagnosed osteoporosis or vertebral fractures. We developed an intervention, 'PHOENIX', to facilitate early detection and management of osteoporosis in people attending hospitals for CT scans.
Methods & analysis: A multi-centre, randomised, pragmatic feasibility study. From the general CT-attending population, women ≥65 and men ≥75 years attending for CT scans are invited to participate, via a novel consent form incorporating FRAX Fracture Risk Assessment questions. Those at increased 10-year risk (within the amber or red zones of the UK FRAX graphical outputs for further action) are block randomised (1:1:1) to 1) PHOENIX intervention, 2) Active Control or 3) Usual Care. The PHOENIX Intervention comprises i) retrieving the CT scans using the NHS Image Exchange Portal, ii) Mindways QCT Pro software analysis of CT Hip and Spine Bone Density with CT Vertebral Fracture Assessment, iii) sending the participants' GP a clinical report including diagnosis, necessary investigations, and recommended treatment. Baseline CT scans from groups 2 and 3 are assessed with the PHOENIX intervention at study end. Assuming 25% attrition, the study is powered to find a predicted superior osteoporosis treatment rate with PHOENIX (20%) versus 16% among patients whose GPs were sent the FRAX questionnaire only (Active Control) and 5% in the Usual Care group. Five hospitals are participating, to determine feasibility. The co-primary feasibility outcome measures are a) ability to randomise 375 patients within 10 months and b) retention of 75% of survivors, completing their 1-year bone health outcome questionnaire. Secondary 1-year outcomes include osteoporosis/vertebral fracture identification rates and osteoporosis treatment rates. Stakeholder acceptability and economic aspects are evaluated.
Ethics and dissemination REF19/EE/0176. Dissemination will be through the Royal Osteoporosis Society.
Trial registration number 14722819

Strengths and limitations of this study:

- First pragmatic randomised controlled study of patients attending radiology CT units, who are randomised to either comprehensive screening for osteoporosis (by applying FRAX questionnaire, CT bone densitometry and CT vertebral fracture detection) or usual care.
- Allows patients the flexibility to self-consent, and tests both the willingness of CT attenders to consent, CT technologies and information flow between trial centres and Cambridge hub
- Limited by an amendment of the primary outcome measure because recruitment during COVID-19 pandemic predicted skew towards the more seriously ill (via cancellation of non-urgent and routine scans). Modifications to both the retention outcome and longer recruitment window were made to take account of pandemic redeployment of medical research team.

Introduction: Older patients (65 years ≥in women, 70 years ≥in men) attending hospital for CT scans may have undiagnosed spine fractures or low bone density and therefore stand to benefit from osteoporosis screening. Timely diagnosis and treatment of osteoporosis benefits many patient outcome domains (pain, quality of life, morbidity, and in the case of zoledronate treatment, mortality). An estimated 43% of over 60-year-olds undergoing CT scans (for abdominal and pelvic problems unrelated to their bones) were found to have osteoporosis or vertebral fractures when scan images were examined using computer-analysis methods [1]. However, radiologists reviewing CT scans reported only 16% of vertebral fractures in routine practice [2], and bone density (BMD) is rarely calculated from CT scans (CT BMD). This is despite CT BMD being approved internationally for the diagnosis of osteoporosis. Also, femoral neck BMD from CT is interchangeable with standard DXA* BMD when using the global individual fracture risk web calculator FRAX (*Dual Energy X-ray Absorptiometry). Indeed, several approved software packages now allow accurate diagnosis of osteoporosis in the hip and spine from routinely acquired CT images, while at the same time facilitating vertebral fractures identification and grading.

Systematic reviews indicate that overlooking osteoporosis or vertebral fractures can lead to serious, preventable pain, disability and health costs (>£2 billion per year from osteoporosis in the UK) [3]. Osteoporosis is a common disease in older women and men: osteoporosis is associated with >200,000 vertebral fractures and >85,000 hip fractures annually in the UK [4]. Osteoporosis-related fractures have a high morbidity and, despite low cost-effective treatments, women and men are increasingly presenting at clinics with advanced multiple vertebral fractures, loss of body height, compressed abdominal contents, dysphagia, severe and chronic pain and reduced mobility. Despite an ageing UK population, osteoporosis remains under-diagnosed and under-treated [5]. The osteoporosis

For peer review only - http://bmjopen.bmj.com/site/about/guidelines.xhtml

case-finding rate in primary care is low and there is no national mandate for screening. High risk patients may be referred for DXA and are offered treatment if fracture risk sufficiently high. Vertebral fractures are infrequently detected in usual care even though they (alongside hip fractures) cause the biggest burden to patients and the health service. We hypothesise that targeted screening will improve upon usual care and improve diagnosis. The PHOENIX intervention is a simple and widely practicable intervention to facilitate osteoporosis identification, diagnosis and treatment in patients attending for routine clinical CT at any hospital. It has been developed to apply software to CT scan images and repurpose them for measuring bone density, fracture risk and to identify crushed vertebrae [6,7]. It couples timely diagnosis with an individualised report (built from a guideline-derived clinical decision tree) sent to GPs containing advice on recommended investigations, NICE-approved, cost-effective treatment/lifestyle advice to reduce osteoporotic fractures [8]. We expect benefits to accrue through early diagnosis and treatment of osteoporosis, with additional justifications for investigating this population shown in Table 1. Preventing fractures could lead to decreased societal and social burden, and reduced health- and social-care costs.

Table 1. Opportunistic osteoporosis screening of CT scan attenders and rationale for PHOENIX Intervention
• CT attenders are a high-risk population: >30% of older adults have undiagnosed vertebral fractures or osteoporosis that can be effectively treated once identified [9]
• The PHOENIX intervention diagnoses osteoporosis on scans already undertaken, so there is no requirement for additional x-ray exposure or hospital attendance
• The PHOENIX intervention can be applied to avoid the necessity of extra DXA visits and DXA reporting in multiple clinical scenarios, such as in the setting of radiology reporting Quality Improvement Programmes that increase CT-diagnosis of vertebral fractures, or to formally diagnose patients with osteoporosis after largescale Artificial Intelligence (AI) vertebral fracture identification methods (e.g. Optasia or ZEBRA AI1tools)
• Screening is effective at reducing hip fractures. A recent trial of primary screening based on clinical risk factors and targeted bone density scanning reduced the hip fracture incidence by 28% over five years, with a number needed to screen (NNS) of 111 to prevent one hip fracture [10]
• Systematic reviews of screening for osteoporosis suggest a number needed to screen (NNS) of 43 people (aged 75 or over) to prevent one vertebral fracture [11]
• Fracture risk is currently assessed opportunistically by GPs. Current advances in IT software could augment this practice to make more efficient and effective use of healthcare resources, as recommended by the National Institute for Health and Care Excellence (NICE), Clinical Guideline (CG) 146 [11]
• A 2013 study found that radiologists failed to report 84% of clinically important vertebral fractures from routine CT scans [4]. However, once combined with new computerised analysis methods, CT scanners can effectively determine bone density and fractures [7]
• There is a fourfold risk of another vertebral fracture occurring after the first one [12] confirming the progressive nature of established osteoporosis
• Generalisability: one million CT scans were performed in the UK in 1996, increasing to five million in 2016 [13], permitting screening of many 'at-risk' patients. Interventions such as PHOENIX are not complicated, nor technically demanding for those hospital staff who routinely analyse DXA scans

Methods and Analysis:
This feasibility study aims to test our PHOENIX intervention, which begins by offering adults attending hospitals for CT (for any reason) a comprehensive bone health screen, followed by 'opportunistic' reuse of their CT scans for bone densitometry. The intervention ends with their GPs receiving detailed written advice on managing the participants' bone health. Patients are offered an invitation pack comprising a novel informed consent form/data capture tool that the patient can fill out with or without assistance, and a participant information sheet. Specifically, we want to determine whether it is feasible for our team to undertake a large trial of the PHOENIX intervention to prevent fractures in a larger population of CT-attenders. Hence our co-primary outcome measure is our ability to randomise 375 'high risk' participants within 10 months. For the larger fracture prevention trial to be feasible, the other co-primary outcome measure must be achieved, which is retention at 12 months; our ability to record bone active treatment data (yes/no) in 75% of surviving patients. Power calculations for any future trial will be informed by the secondary outcome measure; the percentage of participants in each arm who have i) received a treatment recommendation (due to osteoporosis, vertebral fracture or high FRAX risk) and ii) received and commenced treatment 12 months after their CT scan. Table 2 contains a summary of the key features of the study protocol.

The PHOENIX study is a parallel three group, non-blinded randomised controlled study of volunteers aged ≥65-90 (women) or ≥75-90 (men) attending for CT for any clinical reason, where the spine and/or hips are visible in scan images. Specifically, CT-attending patient volunteers who have an elevated 10-year fracture risk (within the amber or red zones of the UK FRAX graphical outputs for further action) are randomised to one of three groups. The three arms allow comparison between [1] the PHOENIX intervention, (2] an alternative Active Control 'middle' group (to assess whether GPs will investigate or treat patients based solely on receipt of a populated FRAX questionnaire), and [3] a control ('Usual Care') group where no action is taken. Potential participants are identified ahead of attendance by the clinical or research team reviewing CT lists. Those eligible by age, sex and region of the body being scanned are handed the PHOENIX pack whilst attending the CT department. The specially designed Informed Consent Form (ICF; Supplemental material file 1) first guides the potential participant through eligibility screening questions (*Do you take medication for your bones once a week that requires you to sit or stand upright? Do you have a yearly infusion (drip) or injection for your bones? Do you have a hip replacement? Have you ever had a hip operation?*). If there is an affirmative answer to any of these questions, individuals are asked to return their forms and go no further. Those individuals who answer 'no' to all the questions, are asked to supply key demographic data: Date of birth, height, weight, country of origin and ethnicity (needed for accurate FRAX calculation) and NHS number (receptionists support the patient as needed with the latter). Individuals are also asked to document the reason for their scan, if they know it.

For peer review only - http://bmjopen.bmj.com/site/about/guidelines.xhtml

**Table 2. Summary of key features of the PHOENIX Study Protocol**
**Pragmatic features:**
Patients attending CT waiting rooms are offered a PHOENIX invitation pack comprising a novel informed consent form/data capture tool to ascertain FRAX score (Supplementary Figure 1) that the patient can fill out with or without assistance
The intervention (tested in Group 1) comprises; i) retrieving the CT scans of those 'amber' or 'red' risk individuals with moderate to high 10-year fracture risk by FRAX, ii) hip and spine BMD analysis with VFA and iii) sending the GP guidance from a Clinical Decision Tree
The intervention take place 'behind the scenes' without impacting upon patient flow or the radiography/radiology workflow
Data (imaging, clinical risk factor, PID including sensitive clinical information) flows via secure encrypted and approved methods including the NHS Image Exchange Portal, PACS and nhs.net secure clinical email
Technical staff can 'pull' clinical CT images for assessment at any time
**Primary Outcome measures/endpoints:**
Feasibility of recruitment; Defined as ability to randomise 375 'at risk' participants over continuous 10-month period
Retention: Defined as retaining 75% of surviving participants to 12 months post randomisation, proven by receipt of follow-up postal questionnaire (specifically an answer to the bone active medication received; yes/no question)
**Secondary Outcome measures/endpoints:**
New vertebral fracture identification across 3 arms assessed at 12 months
Osteoporosis identification across 3 arms assessed at 12 months
Bone active medication initiation across the 3 arms assessed at 12 months
Survival at 12 months across 3 arms assessed at 12 months
**Inclusion criteria: Participants eligible for the trial should fulfil all of the following at randomisation:**
Attending for a routine clinical CT scan
Able to provide informed consent
Aged ⩾65-90 (women) or ⩾75-90 (men) attending for CT for any clinical reason, where the spine and/or hips are visible in scan images
**Exclusion criteria:**
Metalwork in hips (due to streak artefact on the contralateral hip within CT)
Known to be receiving prescription treatment for osteoporosis other than calcium/vitamin D (i.e., bisphosphonate drug, strontium ranelate, denosumab, raloxifene or teriparatide since it would be futile to identify already known and treated osteoporosis)
Prone CT scan (which cannot currently be processed by the software)
**Role of patients and the public**
Actively participated in the study design in focus groups within the project team
Assisted in the design of data collection tools, including the Informed Consent Form and Information Leaflet
Provided guidance on the acceptability of approaching participants in CT scan areas and insight into the anxiety of attending hospital amongst some more elderly members of society. Adding Country of Origin and Ethnicity questions to consent form to facilitate FRAX.
**Impact of COVID-19 pandemic**
Recruitment had to be paused due to the COVID-19 pandemic, then restarted in just one centre without face-to-face contact with patients. Therefore, recruitment was either by clinical approach/discussion versus self-consent based on just handing out recruitment packs after covid-19 restrictions were brought in.

On the last page of the consent form, the eight validated bone health-relevant questions from the FRAX questionnaire are completed, along with patient address, telephone number, GP name and GP address. Completed paperwork is scanned and sent by email attachment within the national, clinically secure NHS.net email account infrastructure to our add-tr.p*******@nhs.net address (except for local patients where the paperwork is collected directly from the CT reception). FRAX answers are processed by the research team at Cambridge who use the results to calculate the 10-year fracture risk score *without* femoral neck BMD online. Those at low risk of fracture (within the green zone of the UK FRAX graphical outputs) are not randomised, but for completeness, these low-risk participants do eventually have the bone parameters in their CT scans reviewed. CT scans from participants in this 'green' category are retrieved later and put through the PHOENIX intervention 13 months after they consented, allowing the study team to ascertain whether FRAX had been effective in picking up patients with osteoporosis/high fracture risk in this CT waiting room setting. Those in the remaining 'red' and 'amber' risk categories are randomised to one of 3 arms, described below: Group 1) The PHOENIX intervention, 2) Active Control (FRAX questionnaire results only sent to GP) and 3) Usual Care. The flow of patients through the study, including randomisation is shown in figure. 1.

Group 1. The PHOENIX intervention. In brief, bone health for the red/amber risk participant is assessed using the CT Images already captured, with computer-aided diagnosis of vertebral fractures performed by one of two trained NHS and accredited bone density technicians using Mindways QCT Pro (Mindways, Austin, Texas USA) in Cambridge. The output is a consultant clinician-verified report (Figure. 2) sent to the GP with details of i) femoral neck and/or spine bone density results, ii) vertebral fracture diagnosis (yes/no/level affected), iii) FRAX estimated 10-year risk of major osteoporotic fracture, iv) any recommended follow-up investigations and a v) simple treat/do not treat decision based on NOGG intervention thresholds, with a suggestion of specific treatments recommended by NICE guidelines. These steps can take place at any time after the patient has gone home from having their CT. Any CT scanner to be used for CT densitometry must be calibrated by reference to a QCT phantom and recalibrated in the event of any x-ray tube change. The technical aspects of the PHOENIX intervention done at study entry in Group 1 (and at study exit in the others two groups) are as follows; all analysis is conducted within the Mindways QCT Pro software tool using one of two standalone hospital-connected PCs as illustrated by screenshots (Figure 3). Technical steps involved in the full PHOENIX intervention are in Table 3.

**Table 3. The PHOENIX Intervention (Group 1): Technical and Practical Steps**

For peer review only - http://bmjopen.bmj.com/site/about/guidelines.xhtml

1)	CT scan retrieved via PACS into Mindways via it DICOM transfer tool (Cambridge) or in the case of the spoke sites, via the UK NHS Image Exchange Portal to Cambridge, followed by importing to Mindways QCT Pro.
2)	The Mindways QCT Pro SlicePick Vertebral Fracture Assessment tool is used to identify any fractures in the imaged CT volume during the bone density assessment, by applying 6-point morphometry criteria to the synthesised lateral image (upper panels of Figure 3). SlicePick works by synthesising AP and sagittal images from the axial CT slices.
3)	Mindways QCT Pro CTXA module is used to measure BMD in the left femoral neck (Figure 3, middle panes), left total hip region (if coverage is sufficient) and/or lumbar spine (L1-L3 as per International Society for Clinical Densitometry (ISCD) guidelines, lower panes Figure 3).
4)	Each individual's FRAX fracture risk estimate is then recalculated online, using clinical RF's from FRAX and the femoral neck BMD value. Importantly, the 'Previous fracture' category is switched to 'yes' in FRAX if spine imaging revealed any vertebral fracture.
5)	The NOGG guidance tool button within FRAX is then used and the pop-up red/green risk charts followed to determine whether treatment is recommended, provided no contra-indications exists. In the case of no femoral neck BMD possible (spinal BMD only), FRAX values are reported (without BMD input) and the PHOENIX clinical decision tree for spine BMD is followed to produce the report that is sent to GPs.
6)	The clinician-verified report phrases are derived from the PHOENIX clinical decision tree (Supplemental material file 2) which is stored within the QCT Pro reporting tool and updated regularly. The report phrases appear (e.g. in Figure 2) on the Hip pdf (CTXA™ Hip Bone Mineral Densitometry, 'GP Advice') analysis and the Spine pdf (Spine 3D QCT Bone Mineral Densitometry 'GP Advice'). The technicians can select the correct GP advice phrase via drop-down menus when they have the FRAX and BMD results to hand. By selecting the matching patient category in the software, a bespoke template is imported into the pdf of the bone density report incorporating all the measured bone health information and clinical risk factors, based on the National Osteoporosis Guideline recommendations.
7)	The final Hip and Spine pdf is signed off electronically by the consultant physician (KESP) via nhs.net email (secure clinical, encrypted) and posted to GPs with a cover letter.
8)	Any vertebral fractures overlooked by radiologists on the RIS report are entered in the local radiology discrepancy review process and the original CT report addended accordingly.
9)	Responsibility for subsequent investigations including referrals for DXA and/or treatment, remain with the GP.
10)	A small, cylindrical Mindways model 4 (asynchronous CT calibration phantom) must be scanned at intervals on each CT scanner, including all the various kVp (kilovoltage peak) energies used clinically, according to a Standard Operating Procedure. This phantom can be posted or taken to radiology departments for QA (Quality Assurance) scanning.

Group 2. Active Control. Only the completed FRAX questionnaire results are sent to the GP, who can follow NOGG guidance themselves by entering responses onto the online FRAX website calculator. However, the patient's data is put through the PHOENIX intervention at study exit (13 months) and results shared with the GP then to enable them to make informed decisions regarding possible treatment.

Group 3. Usual Care. The GP receives a letter informing them of their patient's involvement in the study, but no further reports or information at this point. However, the patient's data is put through the PHOENIX intervention at study exit (13 months) and results shared with the GP then to enable them to make informed decisions regarding possible treatment.

Baseline and one-year follow-up postal questionnaires are sent to all randomised participants to collect data on quality of life and resource use relevant to bone health for health economic analysis. For non-responders a telephone interview is attempted aiming to collect the questionnaire data. Patient and clinician/healthcare worker/administrator perspectives of study involvement are explored through semi-structured interviews by telephone. All study data are held securely processed in accordance with the Data Protection Act 2018 and not disclosed to third parties. Manual records will be held securely (for example in locked filing cabinets). Electronic records will be held on a secure network requiring user ID and password access. Individuals are not identifiable from the REDCAP and JMP (v15, SAS Institute) spreadsheet results of the trial. The Project Management Team members (KW, KESP, DDGC, JMB) are responsible for the day to day running of the study to discuss practical aspects and ensure it is progressing. The Trial Management Group (TMG) comprises the protocol authors and one PPI member. They are responsible for the overall conduct of the study and ensuring NIHR milestones are achieved. Prior to Covid-19 separate PPI meetings were held which enabled those attending to influence the study design.

Sample size calculations, Study power, Randomisation method

Our primary intention is to evaluate the technical procedures needed for a definitive trial with fracture incidence (rather than recruitment/retention) as the primary outcome. To help set the target recruitment numbers for the feasibility study, we therefore used the surrogate outcome of osteoporosis treatment rates at 12 months follow up to permit sufficient power to assess and conduct cross-group comparisons across trial arms within the period of recruitment to the feasibility study. Scenarios for scaling up to a definitive trial are based on previous data [1,2,14] [1, 2, 14]. Only those participants in the red/amber FRAX group (Figure 4a) will be randomised, so the number of invitations needed to reach any recruitment target will also depend on the prevalence of the red/amber FRAX group. In our local preliminary application of the PHOENIX intervention in 1518 patients, the prevalence of red/amber FRAX varied between 30% at age 40 and 50% at age 95 (Figure 4b), with overall prevalence 40.5% (95% CI: 35.7%, 45.5%). There is no published UK data to compare with this but comparable prevalence was reported in a single study in the USA investigating combined bone density and fracture detection in routine CT investigations, where 42.3% (of 571 consecutive patients) were newly detected as having osteoporosis[1]. A conservative assumption from our preliminary work is that the PHOENIX Intervention will identify a further 9.6% of screened patients for treatment and that GPs will act to treat at least half of these, resulting in an expected 4.8% minimum detectable difference. If, in our study, the percentage treated at 12 months in Group 2 (Active Control FRAX-GP arm) matches that observed in the MRC-funded osteoporosis screening SCOOP study[6] (15.5%), then we would consider that an improvement in percentage treated of 4.8% would be clinically significant. Figure 4c. shows the estimated total number of invitees needed to recruit the 375 red/amber risk participants, assuming different response rates and different prevalence estimates. In the

For peer review only - http://bmjopen.bmj.com/site/about/guidelines.xhtml

scenario of 30% response (similar to SCOOP) combined with 40% prevalence of red/amber FRAX (red line in fig 4d) it would be necessary to invite a total of 3,125 participants (or 625 invites per centre) to achieve the recruitment target of 375 randomised participants (75 per centre, Figure 4d.) **Power for recruitment and retention.** Hypothesising that the recruitment (i.e. consent, FRAX completion, eligibility, and CT imaging i.e. 'Response') would be similar to that observed in the SCOOP primary prevention trial (p0 = 30%); a feasibility study aiming to randomise 375 of 938 (40%) consenting participants (i.e. 30% of 3125 total invited) would estimate the trial's response rate with a precision of 1.6% (i.e. half-width of the 95% confidence interval, Figure 4e *Sample size for 80% power to reject null hypothesis of 30% response rate*). The randomised sample of 375 patients will provide 80% power to detect a difference of at least 6% or larger in the 12-month overall trial retention rates from a hypothesised 75% retention (as with the SCOOP trial) based on two-sided one-sample test of proportion at 5% significance level (i.e. providing statistical evidence that the overall retention may be as low or high as 69% (0.69) or 81% (0.81) respectively, as opposed to 75%, 0.75, Figure 4f = *Sample size for 80% power to reject null hypothesis of 75% retention*). In addition, against the same expectation, the 1:1:1 randomised comparison (i.e. PHOENIX Intervention vs. Active Control FRAX to GP vs. Usual Care pathways) will have >80% power to detect at least 12.2% absolute difference in retention rates between trial arms (or RR = 1.16) based on two-sided two-sample test of proportion at 5% significance level. **Power for treatment proportions.** Assuming that osteoporosis treatment percentages at 1-year follow up in the Usual Care vs. Active Control FRAX-GP vs. PHOENIX Intervention would be 4.5% vs. 15.5% vs. 20.3% respectively (i.e. as observed in SCOOP trial vs. with expected improvement in PHOENIX), a total of 375 participants recruited and randomised in the ratio 1:1:1 will have 96% power (Figure 4g) to detect linear trend in treatment proportions based on a two-sided test of binomial proportions at 5% statistical significance level**.** The same sample size will have 93% power for a secondary two-degree of freedom test of between group contrasts in comparison with Usual Care. When allowing for up to 25% attrition due to non-differential dropout or incompleteness of outcomes, the remaining sample size of 280 participants will have >84% power to detect the same magnitude of differences (Figure 4g). **Randomisation method.** Knowing that 375 participants were needed, we were advised to undertake block randomisation stratified by centre, sex and age group (Female: <75 vs ≥75, Male: <80 vs ≥80). Randomisation utilises the web application Research Electronic Data Capture (REDCap https://www.project-redcap.org/).

Statistical analysis Daily screening logs will be kept determining: i) the total number of days (n) where patients were approached in each centre; and ii) the daily totals of PHOENIX invitation packs distributed. This log will also record if a numbered pack was not given out on a day (e.g. if the patient did not arrive for scanning, or the receptionists omitted to give out the pack). The total number of consented individuals (x) as well as the number of consented individuals per day (total x/n overall, and per centre) will be calculated. The number of consented but subsequently excluded individuals (y) will be kept (e.g. for subsequently discovered metalwork in the scan, technical issues with scan). The number of randomised participants will be calculated (z= x-y), and the rate of randomisation (z/n overall, and per centre). These data will be captured from screening logs and entered manually onto a database. The REDCap database will be used to record details of all consenting patients (z). The database will be locked on completion of data entry and when missing data or any discrepancies are resolved. The data will be exported for statistical analysis to the study statisticians with assistance from the Chief Investigator. **Predefined Statistical Analysis Plan**: Response rates will be calculated as the proportion of participants invited who (a) consent to take part, (b) meet eligibility criteria, and (c) are randomised, with 95% confidence intervals to assess the extent to which the realised response deviates from the expected as detailed in the power calculations above. Cumulative participant accrual over time will be plotted overall and by recruitment centre to assess any major differences in accrual and assess progress toward target. Logistic regression will be used to compare randomised groups with respect to the 12-month outcomes of treatment rates and participant retention. Baseline stratification covariates (i.e. centre, sex, and age) will be adjusted for in the models. Analyses will be on an intention-to-treat basis.

Qualitative analysis and Stakeholder acceptability research

Both the baseline and one year follow up questionnaires being sent to all patients include space for participants to feedback their experience of the study. First explaining how this will help, this section includes example open questions such as *"Do you have any comments on being offered a bone health check at the same time as your CT scan? Or on taking part in this research study? /how you were asked to join? /your decision to take part?"*. Semi-structured interviews will be used with a smaller sub-sample of study participants (n=20) to allow for more in-depth exploration of issues that participants may raise in written feedback or during interview. Recordings will be transcribed by an academic transcription service following a confidentiality contract and anonymised by a member of the research team (JF) changing names of people and places and any identifiable details. Key research questions for the PHOENIX process evaluation are; i) what are the implementation facilitators and barriers for the PHOENIX Intervention? ii) how acceptable to the intended patient group is the process of PHOENIX screening? iii) how do implementation and acceptability issues affect uptake of the CT bone health screening and any subsequent GP investigation and recommended bone protective treatment? A combination of interviews and focus groups with those staff groups who will be key to the effective delivery of PHOENIX screening will also be undertaken, both in hospital and primary care, in order to understand their different perspectives. These staff groups are research nurses, receptionists and radiographers in CT departments and GPs. Analyses will be descriptive. The software programme NVivo v12 (QSR) will be used to manage and index the qualitative data (anonymised transcripts of audio-recordings from interviews and focus groups) before charting, mapping and interpretation taking a framework approach [15]. Ritchie & Spencer thematic analysis is an approach suitable for qualitative research in which data collection is relatively focussed on key questions [16,17], hence it is applicable to PHOENIX.

Patient and Public Involvement

At the 'idea' stage of the research, our small team presented our idea at the large workshop *"Find your voice; Demystifying research and public involvement in research"* at Cambridge Professional Development Centre Trumpington in May 2015. This was run by Dr

For peer review only - http://bmjopen.bmj.com/site/about/guidelines.xhtml

Doreen Tembo PPIE lead for the Eastern region NIHR Research Design Service in partnership with the Norfolk and Suffolk PPIRes team (Public and Patient Involvement in Research). This connected us with patients and public representatives, many of whom had relevant musculoskeletal disorders and/or past experience in an orthopaedic service. We collated the opinions and suggestions on our proposed project from every participant in the workshop in writing, individually and from small group discussions to help develop our research question and outcome measures, so that they were informed by patients' priorities, experience, and preferences .Three individuals, Mr Jeremy Dearling, Mrs Tessa Plume and Dr Ann Frost volunteered to join our trial group as PPI representatives; they were specifically involved in patient documentation design (particularly the PHOENIX pack, informed consent form which facilitated consent without having a researcher present) and contributed to suggestions for increasing patient recruitment and follow up. Jane Fleming facilitated group sessions and individual structured interviews to assess the burden of the PHOENIX intervention on patients themselves, as well as a qualitative assessment of the process from a user perspective. The results of the research will be disseminated to surviving participants after generating patient-friendly materials in collaboration with our PPIE representatives.

Economic analysis

Completion rates on the quality-of-life measure (EQ-5D-5L) and resource use items included within the postal survey will inform whether an economic evaluation is feasible using these data collection approaches in the definitive trial. Patterns of response and non-response will be explored to see if they suggest ways the postal survey can be refined to improve data completeness.

Word Count: 3722 (excludes abstract, tables, figures, and references)

Legends for figures:

Figure 1. Study Flow chart for the PHOENIX study

Figure 2. Written clinical guidance and report produced at the end of a PHOENIX Intervention in a typical patient from Group 1. These comprise the standard output pdf's of Mindways QCT Pro which are sent to the participants' GP. In this case there are both hip and spine results from a typical patient with a previously undiagnosed grade 3 vertebral fracture of T12 (found and graded using SlicePick). The femoral neck BMD T-score is -3.03 and spinal osteoporosis is shown with L1-L3 volumetric BMD of 63.4mg/cm^3. Their FRAX 10-year risk of fracture is 25% and investigation/treatment is recommended by NOGG. The written clinical guidance is derived from the PHOENIX clinical decision tree (Supplemental file 2)

Figure 3. The PHOENIX Intervention: technical steps involved in Vertebral Fracture Assessment using Mindways SlicePick to automatically create the sagittal synchronised image in a patient where the technician has used embedded 6-point morphometry to grade a previously undiagnosed T12 vertebral fracture (upper panels). Note the radiopaque bowel contrast in AP and lateral SlicePick views. SlicePick is then used to create a volume for femoral neck (CTXA™) BMD and spine volumetric BMD measurements from the same CT scan. The middle panels show screenshots from CTXA™ femoral neck areal BMD (g/cm^2) analysis from CliniQCT. Note that in this case, only the femoral neck BMD value is taken forwards to the report/guidance (Figure 2), since the CT scan did not cover sufficiently far below the lesser trochanter to allow for 'total hip' BMD measurement. The lower panels show 3D QCT analysis of Lumbar spine L1, L2 and L3 trabecular volumetric BMD (mg/cm^3). Note that Lumbar Spine volumetric BMD T-scores are *not* used in the written clinical guidance and report shown in Figure 2, since trabecular bone spinal vBMD T-scores are not interchangeable with DXA-derived T-scores.

Figure 4. FRAX, sample size and power calculations for PHOENIX a) FRAX UK age-specific fracture risk assessment and intervention thresholds, b) Age-specific prevalence of red/amber FRAX risk category from preliminary local application of PHOENIX intervention in Cambridge, c) Calculation of the number of invites needed to randomise 375 red/amber risk participants based on different responses (rates of agreeing to participate), d) Number of PHOENIX invitation packs that must be given out in order to randomise 375 red/amber risk participants based on different prevalence of red/amber FRAX among participants, e) Our feasibility study aiming to randomise 375 of 938 (40%) consenting participants (i.e. 30% of 3125 total invited) would estimate the trial's response rate with a precision of 1.6% (i.e. half-width of the 95% confidence interval, *this sample size having 80% power to reject null hypothesis at a 30% response*). f) The randomised sample of 375 patients will provide 80% power to detect a difference of at least 6% or larger in the 12-month overall trial retention rates from a hypothesised 75% retention, based on two-sided one-sample test of proportion at 5% significance level (i.e. providing statistical evidence that the overall retention may be as low or high as 69% (0.69) or 81% (0.81) respectively, as opposed to 75%, 0.75, = *Sample size for 80% power to reject null hypothesis of 75% retention*), g) Power vs sample size to detect differences in 12-month treatment rates in our 3-arm trial, assuming treatment percentages of 4.5% vs. 15.5% vs. 20.3% with 1:1:1 randomisation to Usual Care vs. Active Control FRAX-GP vs. PHOENIX Intervention respectively, and type 1 error = 0.05

For peer review only - http://bmjopen.bmj.com/site/about/guidelines.xhtml

Authorship:
All authors agree to be accountable for all aspects of the work to enable questions relating to the accuracy or integrity of any part of the work to be appropriately investigated and resolved

Contributorship statement:
KESP Chief Investigator for the study and is responsible for the concept and design of the overall study and manuscript final approval.
KESP and EMC provided clinical expertise in the area of bone health which forms the basis for the study. They developed the concept and idea for PHOENIX, gained funding and wrote the clinical decision tree with assistance from DDGC. Also methods design, writing and providing feedback of manuscript, manuscript final approval. KESP is responsible for the medical aspects of opportunistic diagnosis of osteoporosis, recommending investigations and treatments to participants.
SKK (with assistance from the relevant trials work of LS, and pilot work of KESP/DDGC) developed the statistical analysis plan including calculation of sample size and definition of primary and secondary outcomes and power calculations.
APW designed the health economics work and developed suitable outcomes for the trial.
DDGC provided information on the background and rationale for using the various software. He has helped clarify pathways for scan analysis in the study and assisted in the development of the Clinical Decision Tree.
KW wrote the protocol and drafted the manuscript, clarified pathways and study flow and liaised with the team to finalise the protocol.
JF designed the process evaluation sub study, with all qualitative interview work, and developed suitable outcomes for the trial.
TDT provided information on the background and rationale for diagnosing vertebral fractures. He was responsible for the medical aspects of opportunistic diagnosis of vertebral fractures.

Acknowledgements: The authors would like to acknowledge Karen Blesic for providing a valuable nursing perspective, for helping develop the idea and for her major contributions towards the NHS Research Innovation Fund pilot to PHOENIX (the clinical service called CORTEX). We acknowledge the contributions of Polly Barnes and Charlotte Tyson to CORTEX and also their contributions towards the practicalities of imaging data flow. We acknowledge Judith Brown's contributions towards the practicalities of patient flow and analysis through the pathways and imaging services. We acknowledge Jeremy Dearling as our primary PPIE representative who gave valuable contributions to the design of the overall study, provided detailed feedback on the materials and protocol development. We also acknowledge the assistance of the Eastern region NIHR Research Design Service team, specifically Jonathan Scales, Andrew Sharpe, Analisa Casarin (improving and developing the research idea and application) and Doreen Tembo (PPI lead for the same). KESP acknowledges the Cambridge NIHR Biomedical Research Centre who support his work, and support DDGC. KESP also thanks the NIHR for funding his attendance at the Pragmatic Clinical Trials Course at Queen Mary University of London which helped him develop the idea. Finally the authors acknowledge the major contribution of Dr Alan Lamont, chair of Cambridge East, Research Ethic Committee whose helpful suggestions and understanding of the relevant GCP issues and legislation rekindled our interest in adopting pragmatic patient consent without a researcher present.

Competing interests statement: No competing interests declared.

Funding: This project is funded by the National Institute for Health Research (NIHR) under its Research for Patient Benefit (RfPB) Programme (Grant Reference Number PB-PG-0816-20027) and by the Cambridge NIHR Biomedical Research Centre (BRC-1215-20014). The views expressed are those of the author(s) and not necessarily those of the NIHR or the Department of Health and Social Care. Funding is in place to 31.03.2022.

Data sharing statement:
Researchers involved in the trial who will have access to the full dataset. Applications for access to the final trial dataset will be through the Trial Management Group. All members of the TMG will have full access to the dataset, and a controlled access model (openly available to all applicants) will be followed as set out in the MRC and NIHR guidance
https://www.methodologyhubs.mrc.ac.uk/files/7114/3682/3831/Datasharingguidance2015.pdf

References

[1] Lee SJ, Binkley N, Lubner MG, et al. Opportunistic screening for osteoporosis using the sagittal reconstruction from routine abdominal CT for combined assessment of vertebral fractures and density. Osteoporosis International 2016;27(3):113, 1-36. doi:10.1007/s00198-0153318-

[2]·Carberry GA, Pooler BD, Binkley N, et al. Unreported Vertebral Body Compression Fractures at Abdominal Multidetector CT. Radiology 2013;268(1):120-26. doi:10.1148/radiol.13121632

[3] Al-Sari UA, Tobias J, Clark E. Health-related quality of life in older people with osteoporotic vertebral fractures: a systematic review and meta-analysis. Osteoporosis International 2016;27(10):2891-900. doi:10.1007/s00198-016-3648-x

For peer review only - http://bmjopen.bmj.com/site/about/guidelines.xhtml

[4] Department of Health. Hospital episode statistics (England) 2006.

[5] Jewell PD, Mitchell R, Javaid MK, et al. Reporting and Follow-up of Vertebral Fractures Seen on Imaging; Can Radiologists Help Reduce the Number of Hip Fractures? Osteoporosis International 2014;25:S674-S74.

[6] Harvey N, Cushnaghan J, Lawrence W, et al. Anxiety, Fracture Risk and Adherence to Treatment: The MRC SCOOP Trial. Osteoporosis International 2012;2 3:S531-S31.

[7] Steiger P, Staal J, Engelke K, et al. Validation of an Automatic Vertebral Prevalent Fracture Classifier Based Upon Full Vertebral Shape. Osteoporosis International 2013;24:S307-S08.

[8] Shepstone L, Lenaghan E, Cooper C, et al. Screening in the community to reduce fractures in older women (SCOOP) - a randomised controlled trial Lancet 2017(pii)S0140-6736(17)32640-5.

[9] OECD (2014). Computed tomography (CT) exams, total. Health: Key Tables from OECD, OECD - ISSN 2075-8480.

[10] National Institute for Health and Care Excellence. Osteoporosis: assessing the risk of fragility fracture 2012. NICE guidelines CG146, 2015

[11] Nelson HD, Haney EM, Dana T, et al. Screening for Osteoporosis: An Update for the US Preventive Services Task Force. Annals of internal medicine 2010;153(2):99-W43. doi:10.7326/0003-4819-153-2-201007200-00262

[12] Lindsay R et al. Risk of new vertebral fracture in the year following a fracture. JAMA. 2001 Jan 17;285(3):320-3

[13] https://www.england.nhs.uk/statistics/wp-content/uploads/sites/2/2016/08/Provisional-Monthly-Diagnostic-Imaging-Dataset-Statistics-2017-05-18.pdf

[14] Pickhardt PJ, Pooler BD, Lauder T, et al. Opportunistic screening for osteoporosis using abdominal computed tomography scans obtained for other indications. Annals of internal medicine 2013;158(8):588-95. doi:10.7326/0003-4819-158-8-201304160-00003

[15] Ritchie J, Spencer L. Qualitative data analysis for applied policy research. In: Bryman A, Burgess R, editors. Analyzing qualitative data. London: Routledge; 1994. p. 173–94.

[16] Moore G, Audrey S, Barker M, Bond L, Bonell C, Hardeman W, Moore L, O'Cathain A, Tinati T, Wight D, Baird J. Process evaluation of complex interventions: Medical Research Council guidance. MRC Population Health Science Research Network, London, 2014

[17] Moore GF, Audrey S, Barker M, Bond L, Bonell C, Hardeman W, Moore L, O'Cathain A, Tinati T, Wight D, Baird J. Process evaluation of complex interventions: Medical Research Council guidance. BMJ 2015;350:h1258 doi:10.1136/bmj.h1258

Revision chronology:
Protocol v1.2 Original 06.10.2020
Protocol v1.3 (amendment 1)
Protocol v2.0 (amendment 2)

Primary reason for Substantial Amendment 1: To allow participants to self-consent to the study during the Covid-19 pandemic. Increase in number of recruits permitted to be obtained from one site (Cambridge) as other sites paused non-Covid research. Additional changes: To allow review of scans when home working using a secure Information Technology (IT) environment

Substantial Amendment 2: Change to one of the primary outcomes related to the study retention at one year to take account of the relatively high mortality in this cohort. The original outcome stated that the study would be considered feasible if treated / not on treatment outcome was determined in ≥75% of the sample at 12 months from randomisation (S3.4.Phoenix_protocol v1.2_2019_07_01). This was amended to ≥75% of those **surviving** at 12 months post randomisation. This change was based on: i) a likely increased disease severity among patients who continued to have CT scans during the first wave of Covid-19 (many non-cancer scans were cancelled, skewing the study sample to those with cancer monitoring); and ii) findings from our own VORTEX Study (Vertebral fracture and Osteoporosis screening from Routine CT as an added EXtra – the VORTEX Study).

Trial Sponsor: Cambridge University NHS Foundation Trust & University of Cambridge

For peer review only - http://bmjopen.bmj.com/site/about/guidelines.xhtml

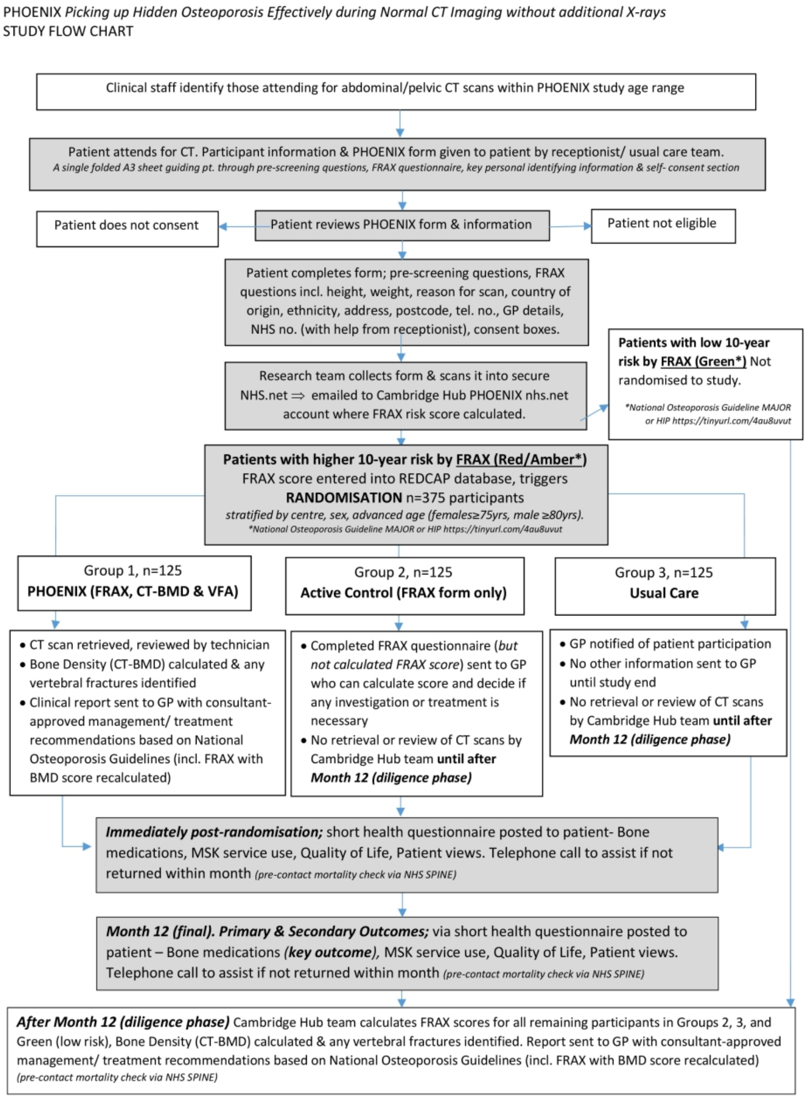

Figure 1. PHOENIX Study flow chart

210x297mm (600 x 600 DPI)

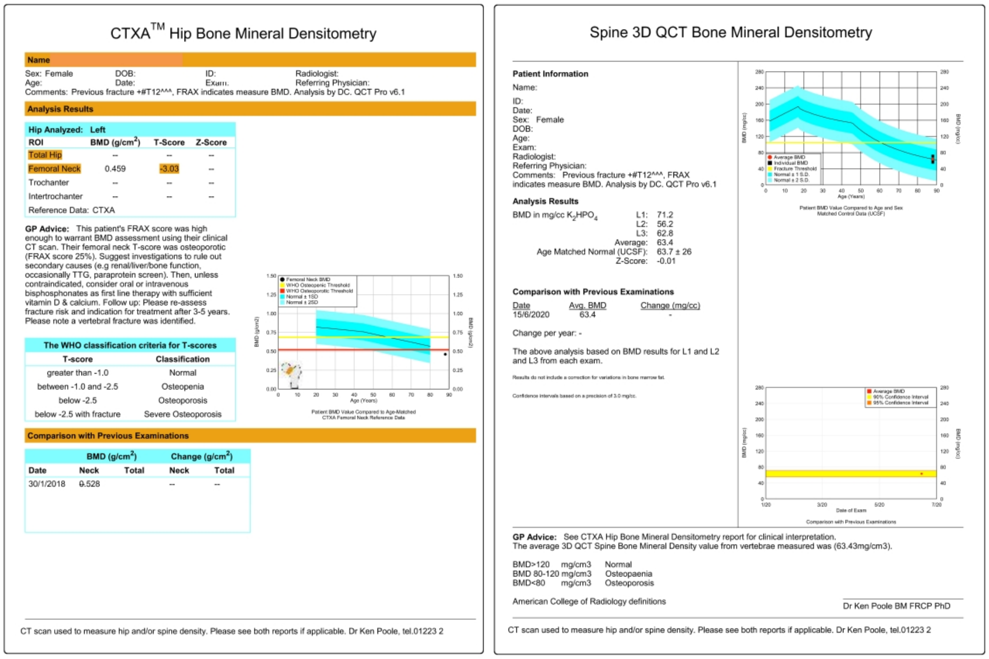

Figure 2. Written clinical guidance and report produced at the end of a PHOENIX Intervention in a typical patient from Group 1. These comprise the standard output pdf's of Mindways QCT Pro which are sent to the participants' GP. In this case there are both hip and spine results from a typical patient with a previously undiagnosed grade 3 vertebral fracture of T12 (found and graded using SlicePick). The femoral neck BMD T-score is -3.03 and spinal osteoporosis is shown with L1-L3 volumetric BMD of 63.4mg/cm3. Their FRAX 10-year risk of fracture is 25% and investigation/treatment is recommended by NOGG. The written clinical guidance is derived from the PHOENIX clinical decision tree (Supplemental file 2)

419x296mm (300 x 300 DPI)

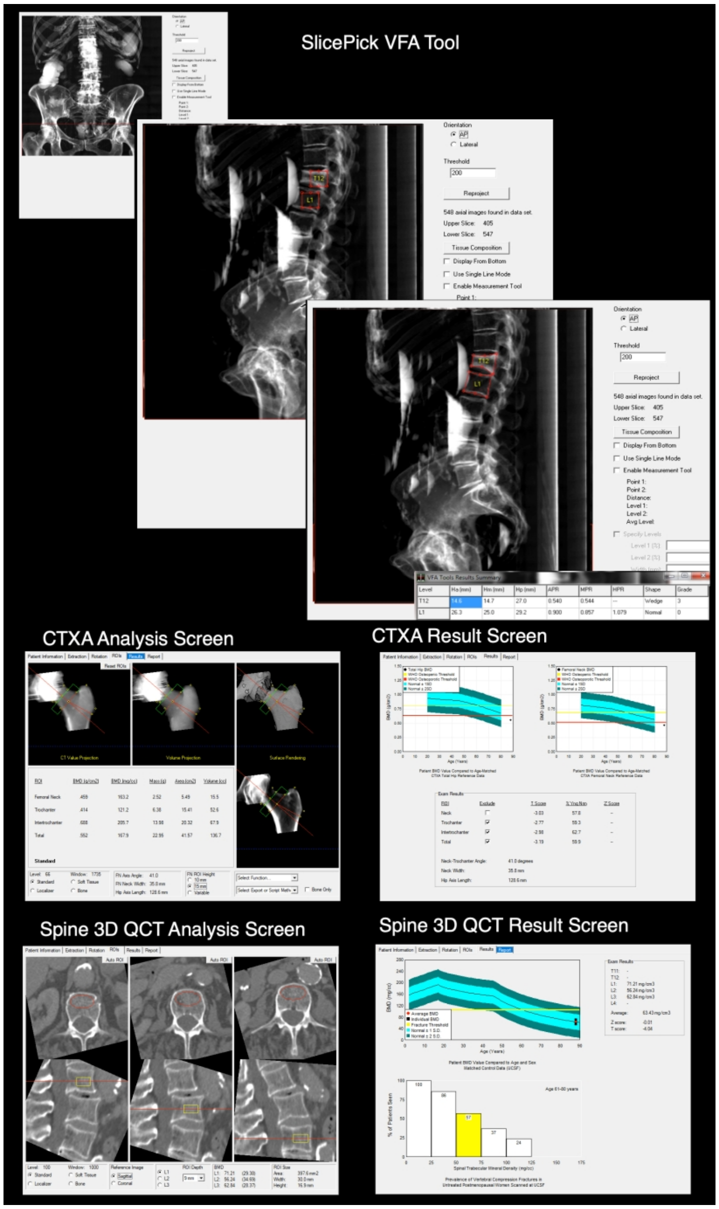

Figure 3. The PHOENIX Intervention: technical steps involved in Vertebral Fracture Assessment using Mindways SlicePick to automatically create the sagittal synchronised image in a patient where the technician has used embedded 6-point morphometry to grade a previously undiagnosed T12 vertebral fracture (upper panels). Note the radiopaque bowel contrast in AP and lateral SlicePick views. SlicePick is then used to create a volume for femoral neck (CTXATM) BMD and spine volumetric BMD measurements from the same CT scan. The middle panels show screenshots from CTXATM femoral neck areal BMD (g/cm2) analysis from CliniQCT. Note that in this case, only the femoral neck BMD value is taken forwards to the report/guidance (Figure 2), since the CT scan did not cover sufficiently far below the lesser trochanter to allow for 'total hip' BMD measurement. The lower panels show 3D QCT analysis of Lumbar spine L1, L2 and L3 trabecular volumetric BMD (mg/cm3). Note that Lumbar Spine volumetric BMD T-scores are not used in the written clinical guidance and report shown in Figure 2, since trabecular bone spinal vBMD T-scores are not interchangeable with DXA-derived T-scores.

151x254mm (300 x 300 DPI)

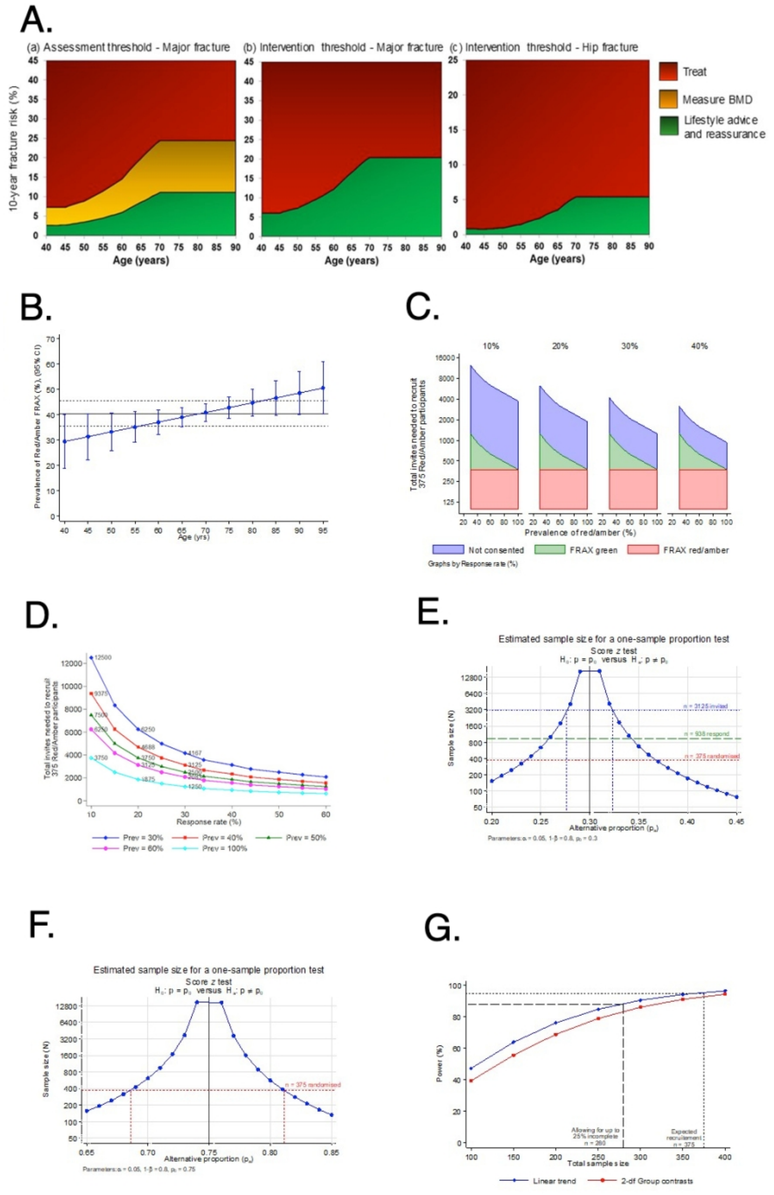

Figure 4. FRAX, sample size and power calculations for PHOENIX a) FRAX UK age-specific fracture risk assessment and intervention thresholds, b) Age-specific prevalence of red/amber FRAX risk category from preliminary local application of PHOENIX intervention in Cambridge, c) Calculation of the number of invites needed to randomise 375 red/amber risk participants based on different responses (rates of agreeing to participate), d) Number of PHOENIX invitation packs that must be given out in order to randomise 375 red/amber risk participants based on different prevalence of red/amber FRAX among participants, e) Our feasibility study aiming to randomise 375 of 938 (40%) consenting participants (i.e. 30% of 3125 total invited) would estimate the trial's response rate with a precision of 1.6% (i.e. half-width of the 95% confidence interval, this sample size having 80% power to reject null hypothesis at a 30% response). f) The randomised sample of 375 patients will provide 80% power to detect a difference of at least 6% or larger in the 12-month overall trial retention rates from a hypothesised 75% retention, based on two- sided one-sample test of proportion at 5% significance level (i.e. providing statistical evidence that the overall retention may be as low or high as 69% (0.69) or 81% (0.81) respectively, as opposed to 75%, 0.75, = Sample size for 80% power to reject null hypothesis of 75% retention), g) Power vs sample size to detect

differences in 12-month treatment rates in our 3-arm trial, assuming treatment percentages of 4.5% vs. 15.5% vs. 20.3% with 1:1:1 randomisation to Usual Care vs. Active Control FRAX-GP vs. PHOENIX Intervention respectively, and type 1 error = 0.05

84x132mm (300 x 300 DPI)

Appendix 1

Osteoporosis is a condition that makes bones become fragile and break more easily, especially in the spine and hips. Osteoporosis can be treated. We would like to diagnose it earlier and are running a study called PHOENIX-f to see if this can be done using the scan that you are having today. There are no extra scans, other tests or treatment required to join the study.

If you are interested in being part of the study, please turn over the page and read on

If you do not wish to take part in the study, can you please tell us why in the box below? This would be really useful for us to know, but you do not have to tell us.

Now please return this form to the PHOENIX-f returns box

Thank you for your co-operation

For peer review only

For peer review only - http://bmjopen.bmj.com/site/about/guidelines.xhtml

By completing this form, we understand that you are happy for one of our research team to talk to you about the study.

Your scan taken today can also be used to measure your bone health.

We can't use every scan, so please answer the following 3 questions to see if we can use yours.

Please circle your answer

**Do you take medication for your bones once a week that require you to sit or stand upright?**	**Y**	**N**
**Do you have a yearly infusion (drip) or injection for your bones?**	**Y**	**N**
**Do you have a hip replacement? Have you ever had a hip operation?**	**Y**	**N**

If you answered 'Yes' to any of the questions above unfortunately we will be unable to use your CT scan today for PHOENIX-f.

Please return this form to the PHOENIX-f returns box.
Thank you for your co-operation.

If you have answered 'NO' to ALL the questions above we will be able to review your CT scan for PHOENIX-f.

In order to understand the study more, please read the patient information leaflet. Then, if you are interested in taking part in PHOENIX to measure your bone health, please fill in the questionnaire overleaf and complete the consent form

For peer review only

For peer review only - http://bmjopen.bmj.com/site/about/guidelines.xhtml

PHOENIX-f Consent Form

Chief Investigator: Dr Ken Poole, Consultant in Rheumatology and Metabolic Bone Disease, Cambridge

Please *initial* boxes below, then sign and date the form and add your address ***Initials***

1. I confirm that I have read and understood the patient information for the study. ☐
2. I understand that my participation is voluntary and that I am free to withdraw at any time without giving any reason, without my medical care or legal rights being affected. ☐
3. If I decide to withdraw from the trial I agree to data already collected to be used for Phoenix-f. ☐
4. I give permission for the study researchers to have access to my scan and relevant NHS databases in order to conduct this research ☐
5. I agree for my personal details and relevant medical information to be sent from my hospital to the study team at Addenbrooke's Hospital for the purpose of the research. I understand that scans have identifiable data and will be kept securely at Addenbrooke's Hospital and will only be accessed by the research team ☐
6. I agree to the research team contacting my GP to notify them of my participation in the study and any results ☐
7. I give permission to be contacted by post or phone by the study team at Addenbrooke's Hospital. I understand that this will be to send me 2 questionnaires and may include an invitation to talk to a researcher about my experience of the study ☐
8. I agree to take part in the above study. ☐

Mr / Mrs / Ms / Dr (please circle as appropriate)

....

Please enter first and family name above

……………………………………………………………. *Date:* ………………………………

Your Signature

Date of Birth: .... *Height:* .... *Weight:* ....

NHS number: ....

Why are you having a scan today? ....

The following questions are required for accurate bone health calculation:

Your country of origin: .... *Ethnicity:* ....

PLEASE TURN OVER PAGE TO COMPLETE THIS FORM

For peer review only - http://bmjopen.bmj.com/site/about/guidelines.xhtml

4

BONE HEALTH QUESTIONNAIRE	Yes	No	Not Sure
Have you broken any bones since you turned 40?			
If Yes, which bones?			
Did your mother or father ever break a hip?			
Do you currently smoke?			
Do you take steroid tablets now (eg: 'prednisolone'), or have you taken steroid tablets in the past, for longer than three months at any time?			
Have you been diagnosed with rheumatoid arthritis?			
Have you been diagnosed with any of the following? **(Tick 'yes' if any of the following apply)**<br>• Premature menopause (before 45yrs of age)<br>• Insulin dependent diabetes<br>• Brittle bone disease, Ankylosing spondylitis<br>• Overactive thyroid<br>• Low testosterone level (men only)<br>• Chronic malnutrition or malabsorption eg: anorexia or coeliac disease<br>• Chronic liver disease			
Do you drink three or more units of alcohol a **day**, on average? (That's 1 1/2 pints of beer or 3 glasses of wine or 3 short measures of spirits)			

Your Address (for questionnaires):

....

....

....

....

Post Code: .... **Your Telephone no:** ....

Your GP Name and address:

....

....

Now please return this form to the PHOENIX-f returns box. Thank you for your co-operation

PHOENIX Methodology paper_2021_04_29_ submission 4

For peer review only

PHOENIX-f Clinical Decision Tree *part 1*

Initial steps

1. Retrieve and visually inspect CT images for any vertebral fractures with AP and lateral localiser views (using Mindways QCT PRO SlicePick for 6-point morphometry).

2. Measure CTXA Hip and/or Spine BMD with QCT PRO, noting Femoral neck & Hip BMD results (in g/cm2) and/or Average L1-L3 Spine BMD results (in mg/cm3).

3. Recalculate FRAX online using the Mindways Femoral neck/Hip BMD (g/cm2) value & change 'Previous Fracture' to 'Yes' if a Grade 2 or 3 fracture is found at step 1.

4. If only one (Hip or Spine) result is available, follow the GP Advice Clinical Decision Tree here to generate automatic GP Advice text.

5. If both Hip BMD and Spine BMD results are available, CTXA Femoral neck/Hip results usually dominate, and the following generic Spine 3D QCT GP advice box text is automatically added, "See CTXA Hip Bone Mineral Densitometry report for clinical interpretation." In GP Advice box add the Spine BMD value and definitions of Normal/ Osteopaenia/Osteoporosis.

6. Only if Spine BMD value is <80mg/cm3 AND NOGG advice is GREEN (after entering Femoral neck/Hip BMD into FRAX) does a Spine result dominate. In this case, the following generic CTXA Hip GP advice box text is automatically added, "Both hip and spine results are available. For this patients' clinical interpretation, please see their associated Spine 3D QCT BMD result".

GP advice text using *Mindways QCT Pro Spine L1-3 BMD value + initial FRAX score/NOGG*

ACR Spine BMD Thresholds

**Abnormal High BMD >200mg/cm^3**	**Normal BMD >120mg/cm^3**		**Osteopenia 120mg/cm^3 ≥ BMD > 80mg/cm^3**		**Osteoporosis BMD ≤ 80mg/cm^3**
	**Initial FRAX Major osteoporotic fracture risk ≤ 20%**	**Initial FRAX Major osteoporotic fracture risk >20%**	**Initial FRAX Major osteoporotic fracture risk ≤ 20%**	**Initial FRAX Major osteoporotic fracture risk >20%**	
This patient had a FRAX score high enough to warrant a BMD assessment which was done using the clinical CT scan. Their BMD of (***) mg/cm3 (*) is higher than expected. High density can be due to degenerative/ genetic effects or for other reasons.	This patient had a FRAX score high enough to warrant a BMD assessment which was done using the clinical CT scan. Their spine BMD >120mg/cm3 (*) is defined as normal bone density by ACR criteria. Unless there is a change in the clinical circumstances, no routine repeat BMD tests are indicated.	This patient had a FRAX score high enough to warrant BMD assessment using the clinical CT scan. Their spine BMD >120mg/cm3 (*) which is defined as normal bone density by ACR criteria. However, there are clinical risk factors which elevate the 10 year future fracture risk (**%).	This patient had a FRAX score high enough to warrant a BMD assessment which was done using the clinical CT scan. Their SPINE BMD of 80-120 mg/cm3 (*) is defined as osteopenia by ACR criteria. There is no current indication for bone active therapy, but the patient may wish to follow the lifestyle advice to maintain bone density (if relevant to patient avoid smoking and excess alcohol intake, and maintain weightbearing exercise.) Please use the online WHO FRAX tool to determine if a standard DXA scan is needed in 3 years.	This patient had a FRAX score high enough to warrant BMD assessment using the clinical CT scan. Their SPINE BMD of 80-120 mg/cm3(*) is defined as osteopenia by ACR criteria. However, there are clinical risk factors which elevate the 10 year future fracture risk above the treatment threshold (**%). Unless contraindicated, consider bisphosphonate treatment.	This patient had a FRAX score high enough (10 year future risk **%) to warrant a BMD assessment using their clinical CT scan. Their SPINE BMD of <80mg/cm3 (*) is defined as osteoporosis by ACR criteria. We suggest investigations to rule out secondary causes (e.g. renal/liver/bone function, low vit D, high PTH, occasionally TTG, paraprotein screen). Then, unless contraindicated, consider oral or intravenous bisphosphonates as first line therapy with sufficient vitamin D & calcium. If relevant to patient, please recommend avoid smoking, excess alcohol intake, & maintain weightbearing exercise. Follow up: Please re-assess fracture risk and indication for treatment after 3-5 years
If grade 2 or 3 vertebral # identified.<br>Include:<br>Please note that a vertebral fracture was identified. Unless this was traumatic/already known, you may wish to investigate further	If grade 2 or 3 vertebral # identified.<br>Include:<br>Please note that a vertebral fracture was identified. Unless this was traumatic/already known, you may wish to investigate further	If grade 2 or 3 vertebral # identified.<br>Include:<br>Please note that a vertebral fracture was identified. Unless this was traumatic/already known, you may wish to investigate further	If grade 2 or 3 vertebral # identified.<br>Include:<br>Please note that a vertebral fracture was identified. Unless this was traumatic/already known, you may wish to investigate further	If grade 2 or 3 vertebral # identified.<br>Include:<br>Please note that a vertebral fracture was identified. Unless this was traumatic/already known, you may wish to investigate further	If grade 2 or 3 vertebral # identified.<br>Include:<br>Please note that a vertebral fracture was identified. Unless this was traumatic/already known, you may wish to investigate further

* Vertebrae analysed

** Major Fracture Risk Score

*** Average BMD value of vertebrae analysed

American College of Radiology

PHOENIX-f Clinical Decision Tree *part 2*

GP advice text using *Mindways QCT Pro CTXA Femoral neck BMD value + BMD-Adjusted FRAX/NOGG*

WHO BMD T-Score Criteria								
**Abnormal High T-Score > 2**	**Normal T-Score > -1**			**Osteopenia -1 ≥ T-Score > -2.5**			**Osteoporosis T-Score ≤ -2.5**	
**Any FRAX/NOGG Threshold**	**Below FRAX /NOGG Treatment Threshold**	**Below FRAX/NOGG Treatment Threshold AND ≥75yrs with prior fragility#/vert #**	**Above FRAX/NOGG Treatment Threshold**	**Below FRAX/NOGG Treatment Threshold**	**Either i) Below FRAX/NOGG Treatment Threshold ≥75yrs with prior fragility#/vert# OR ii) Between -2.0 & -2.50 with prior fragility#/vert#**	**Above FRAX/NOGG Treatment Threshold**	**Below FRAX/NOGG Treatment Threshold**	**Either i) Above FRAX/NOGG Treatment Threshold OR ii) Below treatment threshold with a prior fracture aged ≥75**
This patient has a FRAX score high enough to warrant a BMD assessment which was done using their clinical CT scan. Their *femoral neck/ total hip T-score was (***) which is higher than expected which can have genetic, degenerative or other causes. There is no evidence of osteoporosis	This patient had a FRAX score high enough to warrant a BMD assessment using the clinical CT scan. Their *femoral neck/total hip T-score was >-1 which is defined as normal hip bone density by WHO guidelines (FRAX score **%). Unless there is a change in the clinical circumstances, no routine repeat BMD tests are indicated.	This patient had a FRAX score high enough to warrant a BMD assessment using the clinical CT scan. Their *femoral neck/ total hip T-score was >-1 which is defined as normal hip bone density by WHO guidelines (FRAX score **%). Please note National Guidelines recommend bisphosphonate treatment in the majority of elderly people with a prior fragility fracture regardless of calculated risk. If relevant to patient, recommend avoid smoking, excess alcohol intake, & maintain weightbearing exercise.	This patient had a FRAX score high enough to warrant a BMD assessment using the clinical CT scan. Their *femoral neck/total hip T-score was >-1 which is defined as normal hip bone density by WHO guidelines. However, there are clinical risk factors which elevate the 10 year future fracture risk (FRAX score **%).	This patient had a FRAX score high enough to warrant BMD assessment using the clinical CT scan. Their *femoral neck/ total hip T-score was between -1 and -2.5 which is defined as osteopenia, or low normal bone density, by WHO guidelines. (FRAX score **%). There is no current indication for bone active therapy, but the patient may wish to follow the lifestyle advice to maintain bone density (if relevant to patient avoid smoking and excess alcohol intake, and maintain weightbearing exercise.) Please use the online WHO FRAX tool to determine if a standard DXA scan is needed in 3 years.	This patient had a FRAX score high enough to warrant BMD assessment using the clinical CT scan. Their *femoral neck/total hip T-score was between -1 and > -2.5 which is defined as osteopenia, or low normal bone density, by WHO guidelines (FRAX Score **%). Please note National Guidelines recommend bisphosphonate treatment in the majority of older people with a prior fragility fracture regardless of calculated risk. Unless contraindicated consider bisphosphonate treatment. If relevant to patient, recommend avoid smoking, excess alcohol intake, & maintain weightbearing exercise.	This patient had a FRAX score high enough to warrant BMD assessment using the clinical CT scan. Their *femoral neck/total hip T-score was between -1 and > -2.5 which is defined as osteopenia, or low normal bone density, by WHO guidelines. However, there are clinical risk factors which elevate the 10 year future fracture risk above the treatment threshold (FRAX Score **%). Unless contraindicated consider bisphosphonate treatment. If relevant to patient, recommend avoid smoking, excess alcohol intake, & maintain weightbearing exercise.	This patient's FRAX score was high enough to warrant BMD assessment using their clinical CT scan. Their *femoral neck/total hip T-score was osteoporotic (FRAX score **%). While the scan indicates osteoporosis at the hip, treatment is not automatically recommended (by National Guidelines) at this level of 10-year risk. Suggest investigations to rule out secondary causes (e.g renal/liver/bone function, occasionally TTG, paraprotein screen) The patient may wish to follow the lifestyle advice to maintain bone density (if relevant to patient avoid smoking and excess alcohol intake, and maintain weightbearing exercise.)	This patient's FRAX score was high enough to warrant BMD assessment using their clinical CT scan. Their *femoral neck/ total hip T-score was osteoporotic (FRAX score **%). Suggest investigations to rule out secondary causes (e.g renal/liver/ bone function, occasionally TTG, paraprotein screen). Then, unless contraindicated, consider oral or intravenous bisphosphonates as first line therapy with sufficient vitamin D & calcium. Follow up: Please reassess fracture risk and indication for treatment after 3-5 years
If grade 2 or 3 vertebral # identified<br>Include:<br>Please note that a vertebral fracture was identified, unless this was traumatic/already known, you may wish to investigate further	If grade 2 or 3 vertebral # identified, <75y/o only.<br>Include:<br>Please note that a vertebral fracture was identified, unless this was traumatic/ already known, you may wish to order a spine only DXA scan.	If grade 2 or 3 vertebral # identified.<br>Include:<br>Please note that a vertebral fracture was identified.	If grade 2 or 3 vertebral # identified, <75y/o only.<br>Include:<br>Please note that a vertebral fracture was identified, unless this was traumatic/ already known, you may wish to order a spine only DXA scan."	If grade 2 or 3 vertebral # identified, <75y/o only.<br>Include:<br>Please note that a vertebral fracture was identified, unless this was traumatic/already known, you may wish to order a spine only DXA scan.	If grade 2 or 3 vertebral # identified<br>Include:<br>Please note that a vertebral fracture was identified.	If grade 2 or 3 vertebral # identified<br>Include:<br>Please note that a vertebral fracture was identified.	If grade 2 or 3 vertebral # identified, <75y/o only<br>Include:<br>Please note that a vertebral fracture was identified.	If grade 2 or 3 vertebral # identified<br>Include:<br>Please note that a vertebral fracture was identified.

* Where lowest BMD T-Score was at either the Femoral Neck or Total Hip

** Value of either the Major FRAX score OR the Hip Fracture FRAX score when this value is above the FRAX/NOGG treatment threshold AND Major is below the threshold.

*** Value of the BMD T-Score

Reporting checklist for protocol of a clinical trial.

Based on the SPIRIT guidelines.

Instructions to authors

Complete this checklist by entering the page numbers from your manuscript where readers will find each of the items listed below.

Your article may not currently address all the items on the checklist. Please modify your text to include the missing information. If you are certain that an item does not apply, please write "n/a" and provide a short explanation.

Upload your completed checklist as an extra file when you submit to a journal.

In your methods section, say that you used the SPIRITreporting guidelines, and cite them as:

Chan A-W, Tetzlaff JM, Gøtzsche PC, Altman DG, Mann H, Berlin J, Dickersin K, Hróbjartsson A, Schulz KF, Parulekar WR, Krleža-Jerić K, Laupacis A, Moher D. SPIRIT 2013 Explanation and Elaboration: Guidance for protocols of clinical trials. BMJ. 2013;346:e7586

For peer review only

		Reporting Item	Page Number
**Administrative information**			
Title	#1	Descriptive title identifying the study design, population, interventions, and, if applicable, trial acronym	1

Trial registration	#2a	Trial identifier and registry name. If not yet registered, name of intended registry	1
Trial registration: data set	#2b	All items from the World Health Organization Trial Registration Data Set	1
Protocol version	#3	Date and version identifier	1
Funding	#4	Sources and types of financial, material, and other support	10
Roles and responsibilities: contributorship	#5a	Names, affiliations, and roles of protocol contributors	2
Roles and responsibilities: sponsor contact information	#5b	Name and contact information for the trial sponsor	2
Roles and responsibilities: sponsor and funder	#5c	Role of study sponsor and funders, if any, in study design; collection, management, analysis, and interpretation of data; writing of the report; and the decision to submit the report for publication, including whether they will have ultimate authority over any of these activities	2
Roles and responsibilities: committees	#5d	Composition, roles, and responsibilities of the coordinating centre, steering committee, endpoint adjudication committee, data management team, and	5

For peer review only

		other individuals or groups overseeing the trial, if applicable (see Item 21a for data monitoring committee)	
**Introduction**			
Background and rationale	#6a	Description of research question and justification for undertaking the trial, including summary of relevant studies (published and unpublished) examining benefits and harms for each intervention	3
Background and rationale: choice of comparators	#6b	Explanation for choice of comparators	5
Objectives	#7	Specific objectives or hypotheses	3
Trial design	#8	Description of trial design including type of trial (eg, parallel group, crossover, factorial, single group), allocation ratio, and framework (eg, superiority, equivalence, non-inferiority, exploratory)	4
**Methods: Participants, interventions, and outcomes**			
Study setting	#9	Description of study settings (eg, community clinic, academic hospital) and list of countries where data will be collected. Reference to where list of study sites can be obtained	3

For peer review only

Eligibility criteria	#10	Inclusion and exclusion criteria for participants. If applicable, eligibility criteria for study centres and individuals who will perform the interventions (eg, surgeons, psychotherapists)	7
Interventions: description	#11a	Interventions for each group with sufficient detail to allow replication, including how and when they will be administered	5
Interventions: modifications	#11b	Criteria for discontinuing or modifying allocated interventions for a given trial participant (eg, drug dose change in response to harms, participant request, or improving / worsening disease)	n/a
Interventions: adherance	#11c	Strategies to improve adherence to intervention protocols, and any procedures for monitoring adherence (eg, drug tablet return; laboratory tests)	n/a
Interventions: concomitant care	#11d	Relevant concomitant care and interventions that are permitted or prohibited during the trial	7
Outcomes	#12	Primary, secondary, and other outcomes, including the specific measurement variable (eg, systolic blood pressure), analysis metric (eg, change from baseline, final value, time to event), method of aggregation (eg, median, proportion), and time point for each outcome. Explanation of the clinical relevance of chosen efficacy and harm outcomes is strongly recommended	7

For peer review only

Participant timeline	#13	Time schedule of enrolment, interventions (including any run-ins and washouts), assessments, and visits for participants. A schematic diagram is highly recommended (see Figure)	n/a
Sample size	#14	Estimated number of participants needed to achieve study objectives and how it was determined, including clinical and statistical assumptions supporting any sample size calculations	7
Recruitment	#15	Strategies for achieving adequate participant enrolment to reach target sample size	n/a
**Methods: Assignment of interventions (for controlled trials)**			
Allocation: sequence generation	#16a	Method of generating the allocation sequence (eg, computer-generated random numbers), and list of any factors for stratification. To reduce predictability of a random sequence, details of any planned restriction (eg, blocking) should be provided in a separate document that is unavailable to those who enrol participants or assign interventions	9
Allocation concealment mechanism	#16b	Mechanism of implementing the allocation sequence (eg, central telephone; sequentially numbered, opaque,	n/a

For peer review only

		sealed envelopes), describing any steps to conceal the sequence until interventions are assigned	
Allocation: implementation	#16c	Who will generate the allocation sequence, who will enrol participants, and who will assign participants to interventions	9
Blinding (masking)	#17a	Who will be blinded after assignment to interventions (eg, trial participants, care providers, outcome assessors, data analysts), and how	n/a
Blinding (masking): emergency unblinding	#17b	If blinded, circumstances under which unblinding is permissible, and procedure for revealing a participant's allocated intervention during the trial	n/a
**Methods: Data collection, management, and analysis**			
Data collection plan	#18a	Plans for assessment and collection of outcome, baseline, and other trial data, including any related processes to promote data quality (eg, duplicate measurements, training of assessors) and a description of study instruments (eg, questionnaires, laboratory tests) along with their reliability and validity, if known. Reference to where data collection forms can be found, if not in the protocol	9

For peer review only

Data collection plan: retention	#18b	Plans to promote participant retention and complete follow-up, including list of any outcome data to be collected for participants who discontinue or deviate from intervention protocols	n/a
Data management	#19	Plans for data entry, coding, security, and storage, including any related processes to promote data quality (eg, double data entry; range checks for data values). Reference to where details of data management procedures can be found, if not in the protocol	n/a
Statistics: outcomes	#20a	Statistical methods for analysing primary and secondary outcomes. Reference to where other details of the statistical analysis plan can be found, if not in the protocol	9
Statistics: additional analyses	#20b	Methods for any additional analyses (eg, subgroup and adjusted analyses)	9
Statistics: analysis population and missing data	#20c	Definition of analysis population relating to protocol non-adherence (eg, as randomised analysis), and any statistical methods to handle missing data (eg, multiple imputation)	9

Methods: Monitoring

Data monitoring: formal committee	#21a	Composition of data monitoring committee (DMC); summary of its role and reporting structure; statement of whether it is independent from the sponsor and competing interests; and reference to where further	n/a

For peer review only

		details about its charter can be found, if not in the protocol. Alternatively, an explanation of why a DMC is not needed	
Data monitoring: interim analysis	#21b	Description of any interim analyses and stopping guidelines, including who will have access to these interim results and make the final decision to terminate the trial	n/a
Harms	#22	Plans for collecting, assessing, reporting, and managing solicited and spontaneously reported adverse events and other unintended effects of trial interventions or trial conduct	n/a
Auditing	#23	Frequency and procedures for auditing trial conduct, if any, and whether the process will be independent from investigators and the sponsor	n/a
**Ethics and dissemination**			
Research ethics approval	#24	Plans for seeking research ethics committee / institutional review board (REC / IRB) approval	10
Protocol amendments	#25	Plans for communicating important protocol modifications (eg, changes to eligibility criteria, outcomes, analyses) to relevant parties (eg, investigators, REC / IRBs, trial participants, trial registries, journals, regulators)	1

For peer review only

Consent or assent	#26a	Who will obtain informed consent or assent from potential trial participants or authorised surrogates, and how (see Item 32)	5
Consent or assent: ancillary studies	#26b	Additional consent provisions for collection and use of participant data and biological specimens in ancillary studies, if applicable	n/a
Confidentiality	#27	How personal information about potential and enrolled participants will be collected, shared, and maintained in order to protect confidentiality before, during, and after the trial	5
Declaration of interests	#28	Financial and other competing interests for principal investigators for the overall trial and each study site	10
Data access	#29	Statement of who will have access to the final trial dataset, and disclosure of contractual agreements that limit such access for investigators	10
Ancillary and post trial care	#30	Provisions, if any, for ancillary and post-trial care, and for compensation to those who suffer harm from trial participation	n/a
Dissemination policy: trial results	#31a	Plans for investigators and sponsor to communicate trial results to participants, healthcare professionals, the public, and other relevant groups (eg, via publication, reporting in results databases, or other data sharing arrangements), including any publication restrictions	10

For peer review only

Dissemination policy: authorship	#31b	Authorship eligibility guidelines and any intended use of professional writers	n/a
Dissemination policy: reproducible research	#31c	Plans, if any, for granting public access to the full protocol, participant-level dataset, and statistical code	10

Appendices

Informed consent materials	#32	Model consent form and other related documentation given to participants and authorised surrogates	12
Biological specimens	#33	Plans for collection, laboratory evaluation, and storage of biological specimens for genetic or molecular analysis in the current trial and for future use in ancillary studies, if applicable	n/a

The SPIRIT Explanation and Elaboration paper is distributed under the terms of the Creative Commons Attribution License CC-BY-NC. This checklist was completed on 16. February 2021 using https://www.goodreports.org/, a tool made by the EQUATOR Network in collaboration with Penelope.ai

For peer review only

BMJ Open

PHOENIX (Picking up Hidden Osteoporosis Effectively during Normal CT Imaging without additional X-rays). Protocol for a randomised, multi-centre feasibility study

Journal:	*BMJ Open*
Manuscript ID	bmjopen-2021-050343.R3
Article Type:	Protocol
Date Submitted by the Author:	13-Apr-2022
Complete List of Authors:	Poole, Kenneth; University of Cambridge School of Clinical Medicine; NIHR Cambridge Biomedical Research Centre<br>Chappell, Daniel; Cambridge University Hospitals NHS Foundation Trust, Rheumatology; NIHR Cambridge Biomedical Research Centre<br>Clark, Emma; University of Bristol, Clinical Science at North Bristol<br>Fleming, Jane; University of Cambridge, Cambridge Institute of Public Health; University of Cambridge, Department of Public Health and Primary Care<br>Shepstone, Lee; University of East Anglia<br>Turmezei, Tom; Cambridge University Hospitals NHS Foundation Trust, Radiology; Norfolk and Norwich University Hospital NHS Trust, Radiology<br>Wagner, Adam; NIHR Collaboration for Leadership in Applied Health Research & Care (CLAHRC) East of England; University of East Anglia, Norwich Medical School<br>willoughby, karen; University of Cambridge School of Clinical Medicine, Medicine; NIHR Cambridge Biomedical Research Centre<br>Kaptoge, Stephen; University of Cambridge
**Primary Subject Heading**:	Radiology and imaging
Secondary Subject Heading:	Rheumatology, General practice / Family practice
Keywords:	Bone diseases < ORTHOPAEDIC & TRAUMA SURGERY, Spine < ORTHOPAEDIC & TRAUMA SURGERY, Computed tomography < RADIOLOGY & IMAGING, Diagnostic radiology < RADIOLOGY & IMAGING, RHEUMATOLOGY, Back pain < ORTHOPAEDIC & TRAUMA SURGERY

SCHOLARONE™
Manuscripts

1

PHOENIX (Picking up Hidden Osteoporosis Effectively during Normal CT Imaging without additional X-rays). Protocol for a randomised, multi-centre feasibility study

Authors Kenneth E. S. Poole* (a, b), Daniel DG Chappell (a), Emma M Clark (c), Jane Fleming (b), Lee Shepstone (d), Thomas D Turmezei (a, e) Adam P Wagner (d, f), Karen Willoughby (b), Dr Stephen K Kaptoge (b).
***Corresponding**
(a) Cambridge University Hospitals NHS Trust, (b) University of Cambridge, (c) University of Bristol, (d) University of East Anglia, (e) Norfolk and Norwich University Hospitals NHS Trust, Norwich, (f) National Institute of Health Research (NIHR), Applied Research Collaboration (ARC) East of England (EoE),

Address: Dept of Medicine, University of Cambridge, Level 7, Hills Rd, Cambridge CB2 0QQ **Tel:** 01223 245151 **Email:** kenneth.poole@nhs.net

Key words: Osteoporosis; osteoporotic fracture reduction; opportunistic screening; vertebral fracture

Introduction: Two million out of the UK's 5 million routine diagnostic computed tomography (CT) scans performed each year incorporate the thoraco-lumbar spine or pelvic region. Up to one third reveal undiagnosed osteoporosis or vertebral fractures. We developed an intervention, 'PHOENIX', to facilitate early detection and management of osteoporosis in people attending hospitals for CT scans.
Methods & analysis: A multi-centre, randomised, pragmatic feasibility study. From the general CT-attending population, women ≥65 and men ≥75 years attending for CT scans are invited to participate, via a novel consent form incorporating FRAX Fracture Risk Assessment questions. Those at increased 10-year risk (within the amber or red zones of the UK FRAX graphical outputs for further action) are block randomised (1:1:1) to 1) PHOENIX intervention, 2) Active Control or 3) Usual Care. The PHOENIX Intervention comprises i) retrieving the CT scans using the NHS Image Exchange Portal, ii) Mindways QCT Pro software analysis of CT Hip and Spine Bone Density with CT Vertebral Fracture Assessment, iii) sending the participants' GP a clinical report including diagnosis, necessary investigations, and recommended treatment. Baseline CT scans from groups 2 and 3 are assessed with the PHOENIX intervention at study end. Assuming 25% attrition, the study is powered to find a predicted superior osteoporosis treatment rate with PHOENIX (20%) versus 16% among patients whose GPs were sent the FRAX questionnaire only (Active Control) and 5% in the Usual Care group. Five hospitals are participating, to determine feasibility. The co-primary feasibility outcome measures are a) ability to randomise 375 patients within 10 months and b) retention of 75% of survivors, completing their 1-year bone health outcome questionnaire. Secondary 1-year outcomes include osteoporosis/vertebral fracture identification rates and osteoporosis treatment rates. Stakeholder acceptability and economic aspects are evaluated.
Ethics and dissemination REF19/EE/0176. Dissemination will be through the Royal Osteoporosis Society.
Trial registration number ISRCTN14722819

Strengths and limitations of this study:

- First pragmatic randomised controlled study of patients attending radiology CT units. Individuals randomised to either comprehensive screening for osteoporosis (by applying FRAX questionnaire, CT bone densitometry and CT vertebral fracture detection) or usual care.
- Allows patients the flexibility to self-consent, and tests both the willingness of CT attenders to consent, CT technologies and information flow between trial centres and Cambridge hub
- Limited by an amendment of the primary outcome measure because recruitment during COVID-19 pandemic predicted skew towards the more seriously ill (via cancellation of non-urgent and routine scans). Modifications to both the retention outcome and longer recruitment window were made to take account of pandemic redeployment of medical research team.
- Missing a secondary care direct intervention arm; which would have been ideal to relieve some of the burden from primary care to enact osteoporosis treatments

Introduction: Older patients (65 years ≥in women, 70 years ≥in men) attending hospital for CT scans may have undiagnosed spine fractures or low bone density and therefore stand to benefit from osteoporosis screening. Timely diagnosis and treatment of osteoporosis benefits many patient outcome domains (pain, quality of life, morbidity, and in the case of zoledronate treatment, mortality). An estimated 43% of over 60-year-olds undergoing CT scans (for abdominal and pelvic problems unrelated to their bones) were found to have osteoporosis or vertebral fractures when scan images were examined using computer-analysis methods [1]. However, radiologists reviewing CT scans reported only 16% of vertebral fractures in routine practice [2], and bone density (BMD) is rarely calculated from CT scans (CT BMD). This is despite CT BMD being approved internationally for the diagnosis of osteoporosis. Also, femoral neck BMD from CT is interchangeable with standard DXA* BMD when using the global individual fracture risk web calculator FRAX (*Dual Energy X-ray Absorptiometry). Indeed, several approved software packages now allow accurate diagnosis of osteoporosis in the hip and spine from routinely acquired CT images, while at the same time facilitating vertebral fractures identification and grading.

Systematic reviews indicate that overlooking osteoporosis or vertebral fractures can lead to serious, preventable pain, disability and health costs (>£2 billion per year from osteoporosis in the UK) [3]. Osteoporosis is a common disease in older women and men: osteoporosis is associated with >200,000 vertebral fractures and >85,000 hip fractures annually in the UK [4]. Osteoporosis-related fractures have a high morbidity and, despite low cost-effective treatments, women and men are increasingly presenting at clinics with

For peer review only - http://bmjopen.bmj.com/site/about/guidelines.xhtml

advanced multiple vertebral fractures, loss of body height, compressed abdominal contents, dysphagia, severe and chronic pain and reduced mobility. Despite an ageing UK population, osteoporosis remains under-diagnosed and under-treated [5]. The osteoporosis case-finding rate in primary care is low and there is no national mandate for screening. High risk patients may be referred for DXA and are offered treatment if fracture risk sufficiently high. Vertebral fractures are infrequently detected in usual care even though they (alongside hip fractures) cause the biggest burden to patients and the health service. We hypothesise that targeted screening will improve upon usual care and improve diagnosis. The PHOENIX intervention is a simple and widely practicable intervention to facilitate osteoporosis identification, diagnosis and treatment in patients attending for routine clinical CT at any hospital. It has been developed to apply software to CT scan images and repurpose them for measuring bone density, fracture risk and to identify crushed vertebrae [6,7]. It couples timely diagnosis with an individualised report (built from a guideline-derived clinical decision tree) sent to GPs containing advice on recommended investigations, NICE-approved, cost-effective treatment/lifestyle advice to reduce osteoporotic fractures [8]. We expect benefits to accrue through early diagnosis and treatment of osteoporosis, with additional justifications for investigating this population shown in Table 1. Preventing fractures could lead to decreased societal and social burden, and reduced health- and social-care costs.

Table 1. Opportunistic osteoporosis screening of CT scan attenders and rationale for PHOENIX Intervention

**Table 1.**
• CT attenders are a high-risk population: >30% of older adults have undiagnosed vertebral fractures or osteoporosis that can be effectively treated once identified [9]
• The PHOENIX intervention diagnoses osteoporosis on scans already undertaken, so there is no requirement for additional x-ray exposure or hospital attendance
• The PHOENIX intervention can be applied to avoid the necessity of extra DXA visits and DXA reporting in multiple clinical scenarios, such as in the setting of radiology reporting Quality Improvement Programmes that increase CT-diagnosis of vertebral fractures, or to formally diagnose patients with osteoporosis after largescale Artificial Intelligence (AI) vertebral fracture identification methods (e.g. Optasia or ZEBRA AI1tools)
• Screening is effective at reducing hip fractures. A recent trial of primary screening based on clinical risk factors and targeted bone density scanning reduced the hip fracture incidence by 28% over five years, with a number needed to screen (NNS) of 111 to prevent one hip fracture [10]
• Systematic reviews of screening for osteoporosis suggest a number needed to screen (NNS) of 43 people (aged 75 or over) to prevent one vertebral fracture [11]
• Fracture risk is currently assessed opportunistically by GPs. Current advances in IT software could augment this practice to make more efficient and effective use of healthcare resources, as recommended by the National Institute for Health and Care Excellence (NICE), Clinical Guideline (CG) 146 [11]
• A 2013 study found that radiologists failed to report 84% of clinically important vertebral fractures from routine CT scans [4]. However, once combined with new computerised analysis methods, CT scanners can effectively determine bone density and fractures [7]
• There is a fourfold risk of another vertebral fracture occurring after the first one [12] confirming the progressive nature of established osteoporosis
• Generalisability: one million CT scans were performed in the UK in 1996, increasing to five million in 2016 [13], permitting screening of many 'at-risk' patients. Interventions such as PHOENIX are not complicated, nor technically demanding for those hospital staff who routinely analyse DXA scans

Methods and Analysis:
This feasibility study aims to test our PHOENIX intervention, which begins by offering adults attending hospitals for CT (for any reason) a comprehensive bone health screen, followed by 'opportunistic' reuse of their CT scans for bone densitometry. The intervention ends with their GPs receiving detailed written advice on managing the participants' bone health. Patients are offered an invitation pack comprising a novel informed consent form/data capture tool that the patient can fill out with or without assistance, and a participant information sheet. Specifically, we want to determine whether it is feasible for our team to undertake a large trial of the PHOENIX intervention to prevent fractures in a larger population of CT-attenders. Hence our co-primary outcome measure is our ability to randomise 375 'high risk' participants within 10 months. For the larger fracture prevention trial to be feasible, the other co-primary outcome measure must be achieved, which is retention at 12 months; our ability to record bone active treatment data (yes/no) in 75% of surviving patients. Power calculations for any future trial will be informed by the secondary outcome measure; the percentage of participants in each arm who have i) received a treatment recommendation (due to osteoporosis, vertebral fracture or high FRAX risk) and ii) received and commenced treatment 12 months after their CT scan. Table 2 contains a summary of the key features of the study protocol.

The PHOENIX study is a parallel three group, non-blinded randomised controlled study of volunteers aged ≥65-90 (women) or ≥75-90 (men) attending for CT for any clinical reason, where the spine and/or hips are visible in scan images. Specifically, CT-attending patient volunteers who have an elevated 10-year fracture risk (within the amber or red zones of the UK FRAX graphical outputs for further action) are randomised to one of three groups. The three arms allow comparison between [1] the PHOENIX intervention, (2] an alternative Active Control 'middle' group (to assess whether GPs will investigate or treat patients based solely on receipt of a populated FRAX questionnaire), and [3] a control ('Usual Care') group where no action is taken. Potential participants are identified ahead of attendance by the clinical or research team reviewing CT lists. Those eligible by age, sex and region of the body being scanned are handed the PHOENIX pack whilst attending the CT department. The specially designed Informed Consent Form (ICF; Supplemental material file 1) first guides the potential participant through eligibility screening questions (*Do you take medication for your bones once a week that requires you to sit or stand upright? Do you have a yearly infusion (drip) or injection for your bones? Do you have a hip replacement? Have you ever had a hip operation?*). If there is an affirmative answer to any of these questions,

For peer review only - http://bmjopen.bmj.com/site/about/guidelines.xhtml

individuals are asked to return their forms and go no further. Those individuals who answer 'no' to all the questions, are asked to supply key demographic data: Date of birth, height, weight, country of origin and ethnicity (needed for accurate FRAX calculation) and NHS number (receptionists support the patient as needed with the latter). Individuals are also asked to document the reason for their scan, if they know it.

Table 2. Summary of key features of the PHOENIX Study Protocol. Note that a limitation of our methodology is that the protocol was designed before the pandemic when our standard practice (enacted by Fracture Liaison Service and Rheumatologists/Endocrinologists/Orthogeriatricians) was to ask the GP to commence prescriptions for osteoporosis in the patients that we identify. We have witnessed the pressure and difficulties faced by primary care during the pandemic leading us to consider how we would redesign the study to reflect current healthcare pressures and primary care circumstances;. Hence if it were possible, we would now design the study with an extra arm testing the efficacy of treatments organised by the FLS team or secondary care (Rheumatologists/Endocrinologists/Orthogeriatricians), of course informing the primary care physician of the decision to offer treatment.

Table 2.
**Pragmatic features:**
Patients attending CT waiting rooms are offered a PHOENIX invitation pack comprising a novel informed consent form/data capture tool to ascertain FRAX score (Supplementary Figure 1) that the patient can fill out with or without assistance
The intervention (tested in Group 1) comprises; i) retrieving the CT scans of those 'amber' or 'red' risk individuals with moderate to high 10-year fracture risk by FRAX, ii) hip and spine BMD analysis with VFA and iii) sending the GP guidance from a Clinical Decision Tree
The intervention take place 'behind the scenes' without impacting upon patient flow or the radiography/radiology workflow
Data (imaging, clinical risk factor, PID including sensitive clinical information) flows via secure encrypted and approved methods including the NHS Image Exchange Portal, PACS and nhs.net secure clinical email
Technical staff can 'pull' clinical CT images for assessment at any time
**Primary Outcome measures/endpoints:**
Feasibility of recruitment; Defined as ability to randomise 375 'at risk' participants over continuous 10-month period
Retention: Defined as retaining 75% of surviving participants to 12 months post randomisation, proven by receipt of follow-up postal questionnaire (specifically an answer to the bone active medication received; yes/no question)
**Secondary Outcome measures/endpoints:**
New vertebral fracture identification across 3 arms assessed at 12 months
Osteoporosis identification across 3 arms assessed at 12 months
Bone active medication initiation across the 3 arms assessed at 12 months
Survival at 12 months across 3 arms assessed at 12 months
**Inclusion criteria: Participants eligible for the trial should fulfil all of the following at randomisation:**
Attending for a routine clinical CT scan
Able to provide informed consent
Aged ⩾65-90 (women) or ⩾75-90 (men) attending for CT for any clinical reason, where the spine and/or hips are visible in scan images
**Exclusion criteria:**
Metalwork in hips (due to streak artefact on the contralateral hip within CT)
Known to be receiving prescription treatment for osteoporosis other than calcium/vitamin D (i.e., bisphosphonate drug, strontium ranelate, denosumab, raloxifene or teriparatide since it would be futile to identify already known and treated osteoporosis)
Prone CT scan (which cannot currently be processed by the software)
**Role of patients and the public**
Actively participated in the study design in focus groups within the project team
Assisted in the design of data collection tools, including the Informed Consent Form and Information Leaflet
Provided guidance on the acceptability of approaching participants in CT scan areas and insight into the anxiety of attending hospital amongst some more elderly members of society. Adding Country of Origin and Ethnicity questions to consent form to facilitate FRAX.
**Impact of COVID-19 pandemic**
Recruitment had to be paused due to the COVID-19 pandemic, then restarted in just one centre without face-to-face contact with patients. Therefore, recruitment was either by clinical approach/discussion versus self-consent based on just handing out recruitment packs after covid-19 restrictions were brought in.

On the last page of the consent form, the eight validated bone health-relevant questions from the FRAX questionnaire are completed, along with patient address, telephone number, GP name and GP address. Completed paperwork is scanned and sent by email attachment within the national, clinically secure NHS.net email account infrastructure to our add-tr.p*******@nhs.net address (except for local patients where the paperwork is collected directly from the CT reception). FRAX answers are processed by the research team at Cambridge who use the results to calculate the 10-year fracture risk score *without* femoral neck BMD online. Those at low risk of fracture (within the green zone of the UK FRAX graphical outputs) are not randomised, but for completeness, these low-risk participants do eventually have the bone parameters in their CT scans reviewed. CT scans from participants in this 'green' category are retrieved later and put through the PHOENIX intervention 13 months after they consented, allowing the study team to ascertain whether FRAX had been effective in picking up patients with osteoporosis/high fracture risk in this CT waiting room setting. Those in the remaining 'red' and 'amber' risk categories are randomised to one of 3 arms, described below: Group 1) The PHOENIX intervention, 2) Active Control (FRAX questionnaire results only sent to GP) and 3) Usual Care. The flow of patients through the study, including randomisation is shown in figure. 1.

Group 1. The PHOENIX intervention. In brief, bone health for the red/amber risk participant is assessed using the CT Images already captured, with computer-aided diagnosis of vertebral fractures performed by one of two trained NHS and accredited bone density technicians using Mindways QCT Pro (Mindways, Austin, Texas USA) in Cambridge. The output is a consultant clinician-verified report (Figure. 2) sent to the GP with details of i) femoral neck and/or spine bone density results, ii) vertebral fracture

For peer review only - http://bmjopen.bmj.com/site/about/guidelines.xhtml

diagnosis (yes/no/level affected), iii) FRAX estimated 10-year risk of major osteoporotic fracture, iv) any recommended follow-up investigations and a v) simple treat/do not treat decision based on NOGG intervention thresholds, with a suggestion of specific treatments recommended by NICE guidelines. These steps can take place at any time after the patient has gone home from having their CT. Any CT scanner to be used for CT densitometry must be calibrated by reference to a QCT phantom and recalibrated in the event of any x-ray tube change. The technical aspects of the PHOENIX intervention done at study entry in Group 1 (and at study exit in the others two groups) are as follows; all analysis is conducted within the Mindways QCT Pro software tool using one of two standalone hospital-connected PCs as illustrated by screenshots (Figure 3). Technical steps involved in the full PHOENIX intervention are in Table 3.

Table 3. The PHOENIX Intervention (Group 1): Technical and Practical Steps

**Table 3.**	
1)	CT scan retrieved via PACS into Mindways via it DICOM transfer tool (Cambridge) or in the case of the spoke sites, via the UK NHS Image Exchange Portal to Cambridge, followed by importing to Mindways QCT Pro.
2)	The Mindways QCT Pro SlicePick Vertebral Fracture Assessment tool is used to identify any fractures in the imaged CT volume during the bone density assessment, by applying 6-point morphometry criteria to the synthesised lateral image (upper panels of Figure 3). SlicePick works by synthesising AP and sagittal images from the axial CT slices.
3)	Mindways QCT Pro CTXA module is used to measure BMD in the left femoral neck (Figure 3, middle panes), left total hip region (if coverage is sufficient) and/or lumbar spine (L1-L3 as per International Society for Clinical Densitometry (ISCD) guidelines, lower panes Figure 3).
4)	Each individual's FRAX fracture risk estimate is then recalculated online, using clinical RF's from FRAX and the femoral neck BMD value. Importantly, the 'Previous fracture' category is switched to 'yes' in FRAX if spine imaging revealed any vertebral fracture.
5)	The NOGG guidance tool button within FRAX is then used and the pop-up red/green risk charts followed to determine whether treatment is recommended, provided no contra-indications exists. In the case of no femoral neck BMD possible (spinal BMD only), FRAX values are reported (without BMD input) and the PHOENIX clinical decision tree for spine BMD is followed to produce the report that is sent to GPs.
6)	The clinician-verified report phrases are derived from the PHOENIX clinical decision tree (Supplemental material file 2) which is stored within the QCT Pro reporting tool and updated regularly. The report phrases appear (e.g. in Figure 2) on the Hip pdf (CTXA™ Hip Bone Mineral Densitometry, 'GP Advice') analysis and the Spine pdf (Spine 3D QCT Bone Mineral Densitometry 'GP Advice'). The technicians can select the correct GP advice phrase via drop-down menus when they have the FRAX and BMD results to hand. By selecting the matching patient category in the software, a bespoke template is imported into the pdf of the bone density report incorporating all the measured bone health information and clinical risk factors, based on the National Osteoporosis Guideline recommendations.
7)	The final Hip and Spine pdf is signed off electronically by the consultant physician (KESP) via nhs.net email (secure clinical, encrypted) and posted to GPs with a cover letter.
8)	Any vertebral fractures overlooked by radiologists on the RIS report are entered in the local radiology discrepancy review process and the original CT report addended accordingly.
9)	Responsibility for subsequent investigations including referrals for DXA and/or treatment, remain with the GP.
10)	A small, cylindrical Mindways model 4 (asynchronous CT calibration phantom) must be scanned at intervals on each CT scanner, including all the various kVp (kilovoltage peak) energies used clinically, according to a Standard Operating Procedure. This phantom can be posted or taken to radiology departments for QA (Quality Assurance) scanning.

Group 2. Active Control. Only the completed FRAX questionnaire results are sent to the GP, who can follow NOGG guidance themselves by entering responses onto the online FRAX website calculator. However, the patient's data is put through the PHOENIX intervention at study exit (13 months) and results shared with the GP then to enable them to make informed decisions regarding possible treatment.

Group 3. Usual Care. The GP receives a letter informing them of their patient's involvement in the study, but no further reports or information at this point. However, the patient's data is put through the PHOENIX intervention at study exit (13 months) and results shared with the GP then to enable them to make informed decisions regarding possible treatment.

Baseline and one-year follow-up postal questionnaires are sent to all randomised participants to collect data on quality of life and resource use relevant to bone health for health economic analysis. For non-responders a telephone interview is attempted aiming to collect the questionnaire data. Patient and clinician/healthcare worker/administrator perspectives of study involvement are explored through semi-structured interviews by telephone. All study data are held securely processed in accordance with the Data Protection Act 2018 and not disclosed to third parties. Manual records will be held securely (for example in locked filing cabinets). Electronic records will be held on a secure network requiring user ID and password access. Individuals are not identifiable from the REDCAP and JMP (v15, SAS Institute) spreadsheet results of the trial. The Project Management Team members (KW, KESP, DDGC, JMB) are responsible for the day to day running of the study to discuss practical aspects and ensure it is progressing. The Trial Management Group (TMG) comprises the protocol authors and one PPI member. They are responsible for the overall conduct of the study and ensuring NIHR milestones are achieved. Prior to Covid-19 separate PPI meetings were held which enabled those attending to influence the study design.

Sample size calculations, Study power, Randomisation method

Our primary intention is to evaluate the technical procedures needed for a definitive trial with fracture incidence (rather than recruitment/retention) as the primary outcome. To help set the target recruitment numbers for the feasibility study, we therefore used the surrogate outcome of osteoporosis treatment rates at 12 months follow up to permit sufficient power to assess and conduct cross-group comparisons across trial arms within the period of recruitment to the feasibility study. Scenarios for scaling up to a definitive trial are based on previous data [1,2,14] [1, 2, 14]. Only those participants in the red/amber FRAX group (Figure 4a) will be randomised,

For peer review only - http://bmjopen.bmj.com/site/about/guidelines.xhtml

so the number of invitations needed to reach any recruitment target will also depend on the prevalence of the red/amber FRAX group. In our local preliminary application of the PHOENIX intervention in 1518 patients, the prevalence of red/amber FRAX varied between 30% at age 40 and 50% at age 95 (Figure 4b), with overall prevalence 40.5% (95% CI: 35.7%, 45.5%). There is no published UK data to compare with this but comparable prevalence was reported in a single study in the USA investigating combined bone density and fracture detection in routine CT investigations, where 42.3% (of 571 consecutive patients) were newly detected as having osteoporosis[1]. A conservative assumption from our preliminary work is that the PHOENIX Intervention will identify a further 9.6% of screened patients for treatment and that GPs will act to treat at least half of these, resulting in an expected 4.8% minimum detectable difference. If, in our study, the percentage treated at 12 months in Group 2 (Active Control FRAX-GP arm) matches that observed in the MRC-funded osteoporosis screening SCOOP study[6] (15.5%), then we would consider that an improvement in percentage treated of 4.8% would be clinically significant. Figure 4c. shows the estimated total number of invitees needed to recruit the 375 red/amber risk participants, assuming different response rates and different prevalence estimates. In the scenario of 30% response (similar to SCOOP) combined with 40% prevalence of red/amber FRAX (red line in fig 4d) it would be necessary to invite a total of 3,125 participants (or 625 invites per centre) to achieve the recruitment target of 375 randomised participants (75 per centre, Figure 4d.) **Power for recruitment and retention.** Hypothesising that the recruitment (i.e. consent, FRAX completion, eligibility, and CT imaging i.e. 'Response') would be similar to that observed in the SCOOP primary prevention trial (p0 = 30%); a feasibility study aiming to randomise 375 of 938 (40%) consenting participants (i.e. 30% of 3125 total invited) would estimate the trial's response rate with a precision of 1.6% (i.e. half-width of the 95% confidence interval, Figure 4e *Sample size for 80% power to reject null hypothesis of 30% response rate*). The randomised sample of 375 patients will provide 80% power to detect a difference of at least 6% or larger in the 12-month overall trial retention rates from a hypothesised 75% retention (as with the SCOOP trial) based on two-sided one-sample test of proportion at 5% significance level (i.e. providing statistical evidence that the overall retention may be as low or high as 69% (0.69) or 81% (0.81) respectively, as opposed to 75%, 0.75, Figure 4f = *Sample size for 80% power to reject null hypothesis of 75% retention*)**.** In addition, against the same expectation, the 1:1:1 randomised comparison (i.e. PHOENIX Intervention vs. Active Control FRAX to GP vs. Usual Care pathways) will have >80% power to detect at least 12.2% absolute difference in retention rates between trial arms (or RR = 1.16) based on two-sided two-sample test of proportion at 5% significance level. **Power for treatment proportions.** Assuming that osteoporosis treatment percentages at 1-year follow up in the Usual Care vs. Active Control FRAX-GP vs. PHOENIX Intervention would be 4.5% vs. 15.5% vs. 20.3% respectively (i.e. as observed in SCOOP trial vs. with expected improvement in PHOENIX), a total of 375 participants recruited and randomised in the ratio 1:1:1 will have 96% power (Figure 4g) to detect linear trend in treatment proportions based on a two-sided test of binomial proportions at 5% statistical significance level**.** The same sample size will have 93% power for a secondary two-degree of freedom test of between group contrasts in comparison with Usual Care. When allowing for up to 25% attrition due to non-differential dropout or incompleteness of outcomes, the remaining sample size of 280 participants will have >84% power to detect the same magnitude of differences (Figure 4g). **Randomisation method.** Knowing that 375 participants were needed, we were advised to undertake block randomisation stratified by centre, sex and age group (Female: <75 vs ≥75, Male: <80 vs ≥80). Randomisation utilises the web application Research Electronic Data Capture (REDCap https://www.project-redcap.org/).

Statistical analysis Daily screening logs will be kept determining: i) the total number of days (n) where patients were approached in each centre; and ii) the daily totals of PHOENIX invitation packs distributed. This log will also record if a numbered pack was not given out on a day (e.g. if the patient did not arrive for scanning, or the receptionists omitted to give out the pack). The total number of consented individuals (x) as well as the number of consented individuals per day (total x/n overall, and per centre) will be calculated. The number of consented but subsequently excluded individuals (y) will be kept (e.g. for subsequently discovered metalwork in the scan, technical issues with scan). The number of randomised participants will be calculated (z= x-y), and the rate of randomisation (z/n overall, and per centre). These data will be captured from screening logs and entered manually onto a database. The REDCap database will be used to record details of all consenting patients (z). The database will be locked on completion of data entry and when missing data or any discrepancies are resolved. The data will be exported for statistical analysis to the study statisticians with assistance from the Chief Investigator. **Predefined Statistical Analysis Plan**: Response rates will be calculated as the proportion of participants invited who (a) consent to take part, (b) meet eligibility criteria, and (c) are randomised, with 95% confidence intervals to assess the extent to which the realised response deviates from the expected as detailed in the power calculations above. Cumulative participant accrual over time will be plotted overall and by recruitment centre to assess any major differences in accrual and assess progress toward target. Logistic regression will be used to compare randomised groups with respect to the 12-month outcomes of treatment rates and participant retention. Baseline stratification covariates (i.e. centre, sex, and age) will be adjusted for in the models. Analyses will be on an intention-to-treat basis.

Qualitative analysis and Stakeholder acceptability research

Both the baseline and one year follow up questionnaires being sent to all patients include space for participants to feedback their experience of the study. First explaining how this will help, this section includes example open questions such as *"Do you have any comments on being offered a bone health check at the same time as your CT scan? Or on taking part in this research study? /how you were asked to join? /your decision to take part?"*. Semi-structured interviews will be used with a smaller sub-sample of study participants (n=20) to allow for more in-depth exploration of issues that participants may raise in written feedback or during interview. Recordings will be transcribed by an academic transcription service following a confidentiality contract and anonymised by a member of the research team (JF) changing names of people and places and any identifiable details. Key research questions for the PHOENIX process evaluation are; i) what are the implementation facilitators and barriers for the PHOENIX Intervention? ii) how acceptable to the intended patient group is the process of PHOENIX screening? iii) how do implementation and acceptability issues affect uptake of the CT bone health screening and any subsequent GP investigation and recommended bone protective

For peer review only - http://bmjopen.bmj.com/site/about/guidelines.xhtml

treatment? A combination of interviews and focus groups with those staff groups who will be key to the effective delivery of PHOENIX screening will also be undertaken, both in hospital and primary care, in order to understand their different perspectives. These staff groups are research nurses, receptionists and radiographers in CT departments and GPs. Analyses will be descriptive. The software programme NVivo v12 (QSR) will be used to manage and index the qualitative data (anonymised transcripts of audio-recordings from interviews and focus groups) before charting, mapping and interpretation taking a framework approach [15]. Ritchie & Spencer thematic analysis is an approach suitable for qualitative research in which data collection is relatively focussed on key questions [16,17], hence it is applicable to PHOENIX.

Patient and Public Involvement

At the 'idea' stage of the research, our small team presented our idea at the large workshop *"Find your voice; Demystifying research and public involvement in research"* at Cambridge Professional Development Centre Trumpington in May 2015. This was run by Dr Doreen Tembo PPIE lead for the Eastern region NIHR Research Design Service in partnership with the Norfolk and Suffolk PPIRes team (Public and Patient Involvement in Research). This connected us with patients and public representatives, many of whom had relevant musculoskeletal disorders and/or past experience in an orthopaedic service. We collated the opinions and suggestions on our proposed project from every participant in the workshop in writing, individually and from small group discussions to help develop our research question and outcome measures, so that they were informed by patients' priorities, experience, and preferences .Three individuals, Mr Jeremy Dearling, Mrs Tessa Plume and Dr Ann Frost volunteered to join our trial group as PPI representatives; they were specifically involved in patient documentation design (particularly the PHOENIX pack, informed consent form which facilitated consent without having a researcher present) and contributed to suggestions for increasing patient recruitment and follow up. Jane Fleming facilitated group sessions and individual structured interviews to assess the burden of the PHOENIX intervention on patients themselves, as well as a qualitative assessment of the process from a user perspective. The results of the research will be disseminated to surviving participants after generating patient-friendly materials in collaboration with our PPIE representatives.

Economic analysis

Completion rates on the quality-of-life measure (EQ-5D-5L) and resource use items included within the postal survey will inform whether an economic evaluation is feasible using these data collection approaches in the definitive trial. Patterns of response and non-response will be explored to see if they suggest ways the postal survey can be refined to improve data completeness.

Ethics and Dissemination

Approved by committee (National Research Ethics NRES) East of England (EE) as REF/19/EE/0176. Dissemination will be through the Royal Osteoporosis Society Bone Academy (to patients and public) as well as to clinician peers via National and International bone/rheumatology scientific and clinical meetings.

Word Count: 3722 (excludes abstract, tables, figures, and references)

Legends for figures:

Figure 1. Study Flow chart for the PHOENIX study

Figure 2. Written clinical guidance and report produced at the end of a PHOENIX Intervention in a typical patient from Group 1. These comprise the standard output pdf's of Mindways QCT Pro which are sent to the participants' GP. In this case there are both hip and spine results from a typical patient with a previously undiagnosed grade 3 vertebral fracture of T12 (found and graded using SlicePick). The femoral neck BMD T-score is -3.03 and spinal osteoporosis is shown with L1-L3 volumetric BMD of 63.4mg/cm^3. Their FRAX 10-year risk of fracture is 25% and investigation/treatment is recommended by NOGG. The written clinical guidance is derived from the PHOENIX clinical decision tree (Supplemental file 2)

Figure 3. The PHOENIX Intervention: technical steps involved in Vertebral Fracture Assessment using Mindways SlicePick to automatically create the sagittal synchronised image in a patient where the technician has used embedded 6-point morphometry to grade a previously undiagnosed T12 vertebral fracture (upper panels). Note the radiopaque bowel contrast in AP and lateral SlicePick views. SlicePick is then used to create a volume for femoral neck (CTXA™) BMD and spine volumetric BMD measurements from the same CT scan. The middle panels show screenshots from CTXA™ femoral neck areal BMD (g/cm^2) analysis from CliniQCT. Note that in this case, only the femoral neck BMD value is taken forwards to the report/guidance (Figure 2), since the CT scan did not cover sufficiently far below the lesser trochanter to allow for 'total hip' BMD measurement. The lower panels show 3D QCT analysis of Lumbar spine L1, L2 and L3 trabecular volumetric BMD (mg/cm^3). Note that Lumbar Spine volumetric BMD T-scores are *not* used in the written clinical guidance and report shown in Figure 2, since trabecular bone spinal vBMD T-scores are not interchangeable with DXA-derived T-scores.

Figure 4. FRAX, sample size and power calculations for PHOENIX a) FRAX UK age-specific fracture risk assessment and intervention thresholds, b) Age-specific prevalence of red/amber FRAX risk category from preliminary local application of PHOENIX intervention in Cambridge, c) Calculation of the number of invites needed to randomise 375 red/amber risk participants based on different responses (rates of agreeing to participate), d) Number of PHOENIX invitation packs that must be given out in order to randomise 375 red/amber risk participants based on different prevalence of red/amber FRAX among participants, e) Our feasibility study aiming to randomise 375 of 938 (40%) consenting participants (i.e. 30% of 3125 total invited) would estimate the trial's response rate with a precision of 1.6% (i.e. half-width of the 95% confidence interval, *this sample size having 80% power to reject null hypothesis at a 30% response*). f) The randomised sample of 375 patients will provide 80% power to detect a difference of at least 6% or larger in the 12-month overall trial retention rates from a hypothesised 75% retention, based on two-sided one-sample test of proportion at 5% significance level (i.e. providing statistical evidence that the overall retention may be as low or high as 69% (0.69) or 81% (0.81) respectively, as opposed to 75%, 0.75, = *Sample size for 80% power to reject null hypothesis of 75% retention*), g) Power vs sample size to detect differences in 12-month treatment rates in our 3-arm trial, assuming treatment percentages of 4.5% vs. 15.5% vs. 20.3% with 1:1:1 randomisation to Usual Care vs. Active Control FRAX-GP vs. PHOENIX Intervention respectively, and type 1 error = 0.05

For peer review only - http://bmjopen.bmj.com/site/about/guidelines.xhtml

Authorship:
All authors agree to be accountable for all aspects of the work to enable questions relating to the accuracy or integrity of any part of the work to be appropriately investigated and resolved

Contributorship statement:
KESP Chief Investigator for the study and is responsible for the concept and design of the overall study and manuscript final approval.
KESP and EMC provided clinical expertise in the area of bone health which forms the basis for the study. They developed the concept and idea for PHOENIX, gained funding and wrote the clinical decision tree with assistance from DDGC. Also methods design, writing and providing feedback of manuscript, manuscript final approval. KESP is responsible for the medical aspects of opportunistic diagnosis of osteoporosis, recommending investigations and treatments to participants.
SKK (with assistance from the relevant trials work of LS, and pilot work of KESP/DDGC) developed the statistical analysis plan including calculation of sample size and definition of primary and secondary outcomes and power calculations.
APW designed the health economics work and developed suitable outcomes for the trial.
DDGC provided information on the background and rationale for using the various software. He has helped clarify pathways for scan analysis in the study and assisted in the development of the Clinical Decision Tree.
KW wrote the protocol and drafted the manuscript, clarified pathways and study flow and liaised with the team to finalise the protocol.
JF designed the process evaluation sub study, with all qualitative interview work, and developed suitable outcomes for the trial.
TDT provided information on the background and rationale for diagnosing vertebral fractures. He was responsible for the medical aspects of opportunistic diagnosis of vertebral fractures.

Acknowledgements: The PHOENIX study is dedicated to the memory of J. Keenan Brown, inventor of QCT Pro (9th February 1960 to 11th December 2021). Without Keenan, this study would not have been possible. The authors would like to acknowledge Karen Blesic for providing a valuable nursing perspective, for helping develop the idea and for her major contributions towards the NHS Research Innovation Fund pilot to PHOENIX (the clinical service called CORTEX). We acknowledge the contributions of Polly Barnes and Charlotte Tyson to CORTEX and also their contributions towards the practicalities of imaging data flow. We acknowledge Judith Brown's contributions towards the practicalities of patient flow and analysis through the pathways and imaging services. We acknowledge Jeremy Dearling as our primary PPIE representative who gave valuable contributions to the design of the overall study, provided detailed feedback on the materials and protocol development. We also acknowledge the assistance of the Eastern region NIHR Research Design Service team, specifically Jonathan Scales, Andrew Sharpe, Analisa Casarin (improving and developing the research idea and application) and Doreen Tembo (PPI lead for the same). KESP acknowledges the Cambridge NIHR Biomedical Research Centre who support his work, and support DDGC. KESP also thanks the NIHR for funding his attendance at the Pragmatic Clinical Trials Course at Queen Mary University of London which helped him develop the idea. Finally the authors acknowledge the major contribution of Dr Alan Lamont, chair of East of England (EE), Research Ethic Committee whose helpful suggestions and understanding of the relevant GCP issues and legislation rekindled our interest in adopting pragmatic patient consent without a researcher present.

Competing interests statement: No competing interests declared.

Funding: This project is funded by the National Institute for Health Research (NIHR) under its Research for Patient Benefit (RfPB) Programme (Grant Reference Number PB-PG-0816-20027) and by the Cambridge NIHR Biomedical Research Centre (BRC-1215-20014). The views expressed are those of the author(s) and not necessarily those of the NIHR or the Department of Health and Social Care. Funding is in place to 31.03.2022.

Data sharing statement:
Researchers involved in the trial who will have access to the full dataset. Applications for access to the final trial dataset will be through the Trial Management Group. All members of the TMG will have full access to the dataset, and a controlled access model (openly available to all applicants) will be followed as set out in the MRC and NIHR guidance https://www.methodologyhubs.mrc.ac.uk/files/7114/3682/3831/Datasharingguidance2015.pdf

References

[1] Lee SJ, Binkley N, Lubner MG, et al. Opportunistic screening for osteoporosis using the sagittal reconstruction from routine abdominal CT for combined assessment of vertebral fractures and density. Osteoporosis International 2016;27(3):113, 1-36. doi:10.1007/s00198-0153318-

[2] Carberry GA, Pooler BD, Binkley N, et al. Unreported Vertebral Body Compression Fractures at Abdominal Multidetector CT. Radiology 2013;268(1):120-26. doi:10.1148/radiol.13121632

[3] Al-Sari UA, Tobias J, Clark E. Health-related quality of life in older people with osteoporotic vertebral fractures: a systematic review and meta-analysis. Osteoporosis International 2016;27(10):2891-900. doi:10.1007/s00198-016-3648-x

For peer review only - http://bmjopen.bmj.com/site/about/guidelines.xhtml

[4] Department of Health. Hospital episode statistics (England) 2006.

[5] Jewell PD, Mitchell R, Javaid MK, et al. Reporting and Follow-up of Vertebral Fractures Seen on Imaging; Can Radiologists Help Reduce the Number of Hip Fractures? Osteoporosis International 2014;25:S674-S74.

[6] Harvey N, Cushnaghan J, Lawrence W, et al. Anxiety, Fracture Risk and Adherence to Treatment: The MRC SCOOP Trial. Osteoporosis International 2012;2 3:S531-S31.

[7] Steiger P, Staal J, Engelke K, et al. Validation of an Automatic Vertebral Prevalent Fracture Classifier Based Upon Full Vertebral Shape. Osteoporosis International 2013;24:S307-S08.

[8] Shepstone L, Lenaghan E, Cooper C, et al. Screening in the community to reduce fractures in older women (SCOOP) - a randomised controlled trial Lancet 2017(pii)S0140-6736(17)32640-5.

[9] OECD (2014). Computed tomography (CT) exams, total. Health: Key Tables from OECD, OECD - ISSN 2075-8480.

[10] National Institute for Health and Care Excellence. Osteoporosis: assessing the risk of fragility fracture 2012. NICE guidelines CG146, 2015

[11] Nelson HD, Haney EM, Dana T, et al. Screening for Osteoporosis: An Update for the US Preventive Services Task Force. Annals of internal medicine 2010;153(2):99-W43. doi:10.7326/0003-4819-153-2-201007200-00262

[12] Lindsay R et al. Risk of new vertebral fracture in the year following a fracture. JAMA. 2001 Jan 17;285(3):320-3

[13] https://www.england.nhs.uk/statistics/wp-content/uploads/sites/2/2016/08/Provisional-Monthly-Diagnostic-Imaging-Dataset-Statistics-2017-05-18.pdf

[14] Pickhardt PJ, Pooler BD, Lauder T, et al. Opportunistic screening for osteoporosis using abdominal computed tomography scans obtained for other indications. Annals of internal medicine 2013;158(8):588-95. doi:10.7326/0003-4819-158-8-201304160-00003

[15] Ritchie J, Spencer L. Qualitative data analysis for applied policy research. In: Bryman A, Burgess R, editors. Analyzing qualitative data. London: Routledge; 1994. p. 173–94.

[16] Moore G, Audrey S, Barker M, Bond L, Bonell C, Hardeman W, Moore L, O'Cathain A, Tinati T, Wight D, Baird J. Process evaluation of complex interventions: Medical Research Council guidance. MRC Population Health Science Research Network, London, 2014

[17] Moore GF, Audrey S, Barker M, Bond L, Bonell C, Hardeman W, Moore L, O'Cathain A, Tinati T, Wight D, Baird J. Process evaluation of complex interventions: Medical Research Council guidance. BMJ 2015;350:h1258 doi:10.1136/bmj.h1258

Revision chronology:
Protocol v1.2 Original 06.10.2020
Protocol v1.3 (amendment 1)
Protocol v2.0 (amendment 2)

Primary reason for Substantial Amendment 1: To allow participants to self-consent to the study during the Covid-19 pandemic. Increase in number of recruits permitted to be obtained from one site (Cambridge) as other sites paused non-Covid research. Additional changes: To allow review of scans when home working using a secure Information Technology (IT) environment

Substantial Amendment 2: Change to one of the primary outcomes related to the study retention at one year to take account of the relatively high mortality in this cohort. The original outcome stated that the study would be considered feasible if treated / not on treatment outcome was determined in ≥75% of the sample at 12 months from randomisation (S3.4.Phoenix_protocol v1.2_2019_07_01). This was amended to ≥75% of those **surviving** at 12 months post randomisation. This change was based on: i) a likely increased disease severity among patients who continued to have CT scans during the first wave of Covid-19 (many non-cancer scans were cancelled, skewing the study sample to those with cancer monitoring); and ii) findings from our own VORTEX Study (Vertebral fracture and Osteoporosis screening from Routine CT as an added EXtra – the VORTEX Study).

Trial Sponsor: Cambridge University NHS Foundation Trust & University of Cambridge

For peer review only - http://bmjopen.bmj.com/site/about/guidelines.xhtml

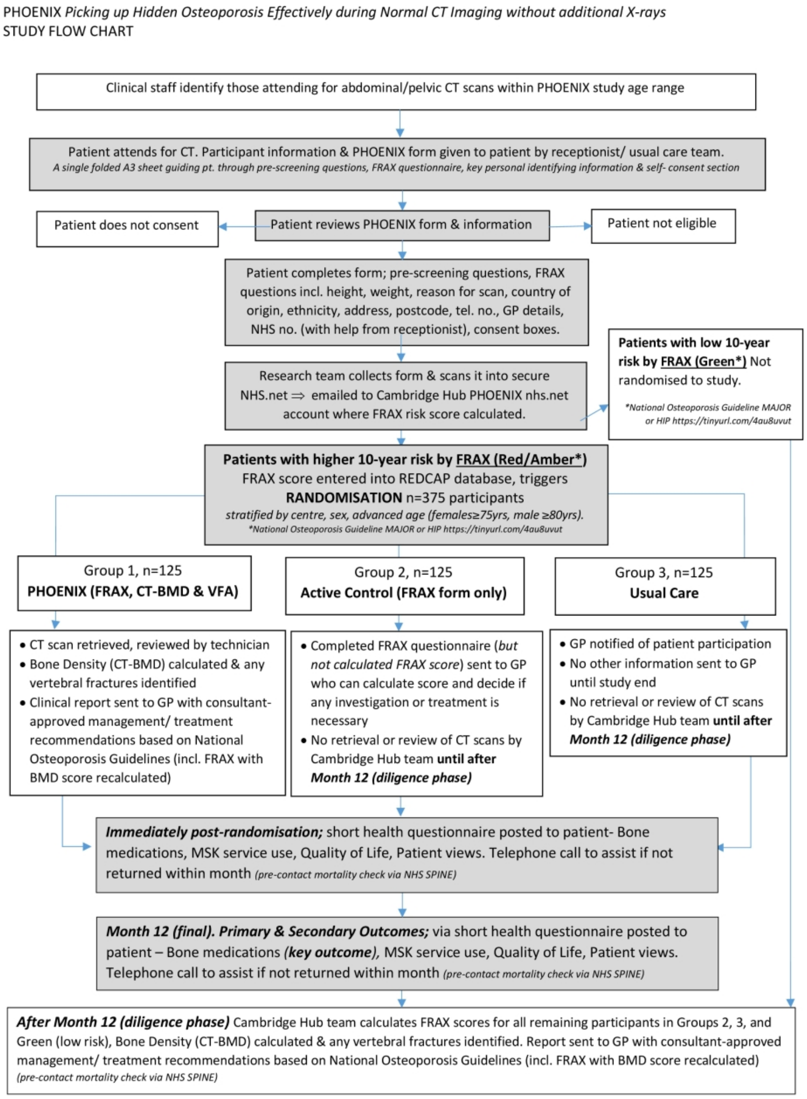

Figure 1. PHOENIX Study flow chart

210x297mm (600 x 600 DPI)

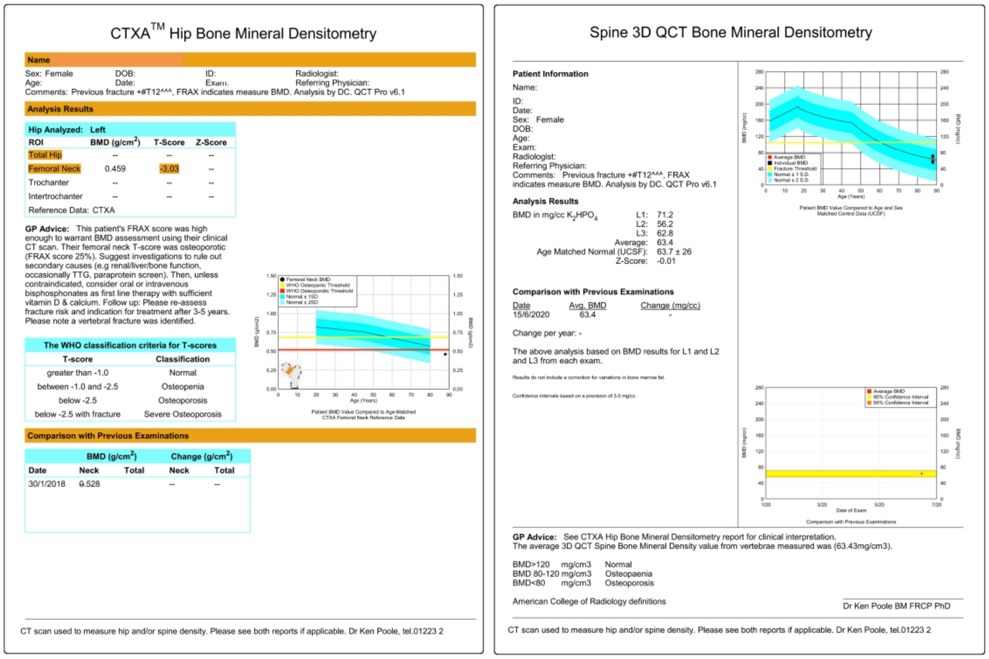

CTXA™ Hip Bone Mineral Densitometry

Name

Sex: Female DOB: ID: Radiologist:
Age: Date: Exam: Referring Physician:
Comments: Previous fracture +#T12^^^, FRAX indicates measure BMD. Analysis by DC. QCT Pro v6.1

Analysis Results

Hip Analyzed: Left

ROI	BMD (g/cm^2)	T-Score	Z-Score
Total Hip	--	--	--
Femoral Neck	0.459	-3.03	--
Trochanter	--	--	--
Intertrochanter	--	--	--

Reference Data: CTXA

GP Advice: This patient's FRAX score was high enough to warrant BMD assessment using their clinical CT scan. Their femoral neck T-score was osteoporotic (FRAX score 25%). Suggest investigations to rule out secondary causes (e.g renal/liver/bone function, occasionally TTG, paraprotein screen). Then, unless contraindicated, consider oral or intravenous bisphosphonates as first line therapy with sufficient vitamin D & calcium. Follow up: Please re-assess fracture risk and indication for treatment after 3-5 years. Please note a vertebral fracture was identified.

The WHO classification criteria for T-scores

T-score	Classification
greater than -1.0	Normal
between -1.0 and -2.5	Osteopenia
below -2.5	Osteoporosis
below -2.5 with fracture	Severe Osteoporosis

Comparison with Previous Examinations

	BMD (g/cm^2)		Change (g/cm^2)	
Date	Neck	Total	Neck	Total
30/1/2018	0.528		--	--

CT scan used to measure hip and/or spine density. Please see both reports if applicable. Dr Ken Poole, tel.01223 2

Spine 3D QCT Bone Mineral Densitometry

Patient Information

Name:

ID:
Date:
Sex: Female
DOB:
Age:
Exam:
Radiologist:
Referring Physician:
Comments: Previous fracture +#T12^^^, FRAX indicates measure BMD. Analysis by DC. QCT Pro v6.1

Analysis Results

BMD in mg/cc K_2HPO_4
L1: 71.2
L2: 56.2
L3: 62.8
Average: 63.4
Age Matched Normal (UCSF): 63.7 ± 26
Z-Score: -0.01

Comparison with Previous Examinations

Date	Avg. BMD	Change (mg/cc)
15/6/2020	63.4	-

Change per year: -

The above analysis based on BMD results for L1 and L2 and L3 from each exam.

Results do not include a correction for variations in bone marrow fat.

Confidence intervals based on a precision of 3.0 mg/cc.

GP Advice: See CTXA Hip Bone Mineral Densitometry report for clinical interpretation.
The average 3D QCT Spine Bone Mineral Density value from vertebrae measured was (63.43mg/cm3).

BMD>120 mg/cm3 Normal
BMD 80-120 mg/cm3 Osteopaenia
BMD<80 mg/cm3 Osteoporosis

American College of Radiology definitions

Dr Ken Poole BM FRCP PhD

CT scan used to measure hip and/or spine density. Please see both reports if applicable. Dr Ken Poole, tel.01223 2

Figure 2. Written clinical guidance and report produced at the end of a PHOENIX Intervention in a typical patient from Group 1. These comprise the standard output pdf's of Mindways QCT Pro which are sent to the participants' GP. In this case there are both hip and spine results from a typical patient with a previously undiagnosed grade 3 vertebral fracture of T12 (found and graded using SlicePick). The femoral neck BMD T-score is -3.03 and spinal osteoporosis is shown with L1-L3 volumetric BMD of 63.4mg/cm3. Their FRAX 10-year risk of fracture is 25% and investigation/treatment is recommended by NOGG. The written clinical guidance is derived from the PHOENIX clinical decision tree (Supplemental file 2)

419x296mm (300 x 300 DPI)

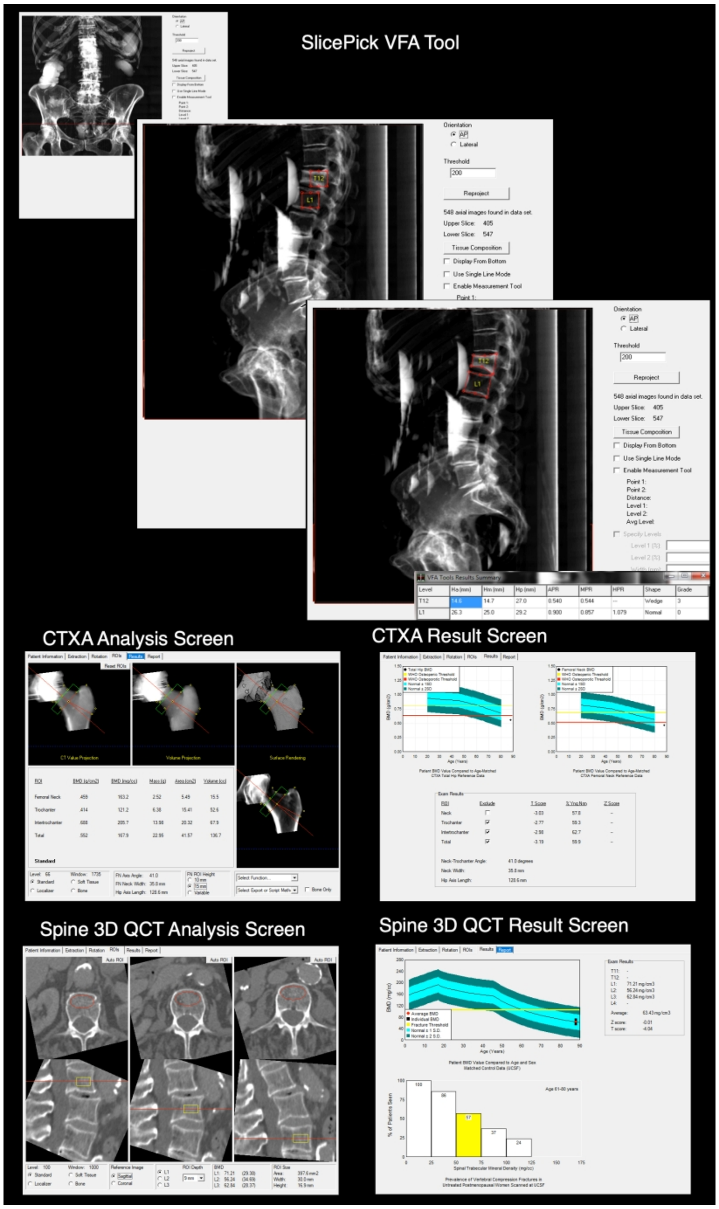

Figure 3. The PHOENIX Intervention: technical steps involved in Vertebral Fracture Assessment using Mindways SlicePick to automatically create the sagittal synchronised image in a patient where the technician has used embedded 6-point morphometry to grade a previously undiagnosed T12 vertebral fracture (upper panels). Note the radiopaque bowel contrast in AP and lateral SlicePick views. SlicePick is then used to create a volume for femoral neck (CTXATM) BMD and spine volumetric BMD measurements from the same CT scan. The middle panels show screenshots from CTXATM femoral neck areal BMD (g/cm2) analysis from CliniQCT. Note that in this case, only the femoral neck BMD value is taken forwards to the report/guidance (Figure 2), since the CT scan did not cover sufficiently far below the lesser trochanter to allow for 'total hip' BMD measurement. The lower panels show 3D QCT analysis of Lumbar spine L1, L2 and L3 trabecular volumetric BMD (mg/cm3). Note that Lumbar Spine volumetric BMD T-scores are not used in the written clinical guidance and report shown in Figure 2, since trabecular bone spinal vBMD T-scores are not interchangeable with DXA-derived T-scores.

151x254mm (300 x 300 DPI)

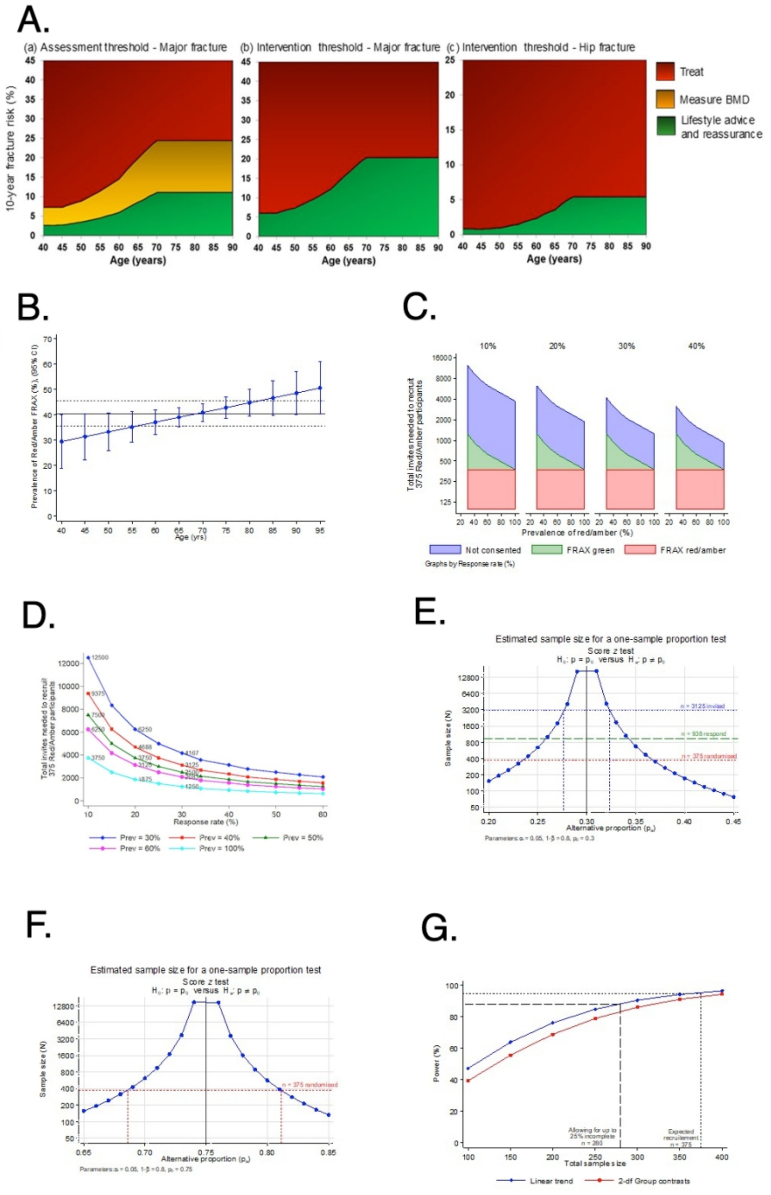

Figure 4. FRAX, sample size and power calculations for PHOENIX a) FRAX UK age-specific fracture risk assessment and intervention thresholds, b) Age-specific prevalence of red/amber FRAX risk category from preliminary local application of PHOENIX intervention in Cambridge, c) Calculation of the number of invites needed to randomise 375 red/amber risk participants based on different responses (rates of agreeing to participate), d) Number of PHOENIX invitation packs that must be given out in order to randomise 375 red/amber risk participants based on different prevalence of red/amber FRAX among participants, e) Our feasibility study aiming to randomise 375 of 938 (40%) consenting participants (i.e. 30% of 3125 total invited) would estimate the trial's response rate with a precision of 1.6% (i.e. half-width of the 95% confidence interval, this sample size having 80% power to reject null hypothesis at a 30% response). f) The randomised sample of 375 patients will provide 80% power to detect a difference of at least 6% or larger in the 12-month overall trial retention rates from a hypothesised 75% retention, based on two- sided one-sample test of proportion at 5% significance level (i.e. providing statistical evidence that the overall retention may be as low or high as 69% (0.69) or 81% (0.81) respectively, as opposed to 75%, 0.75, = Sample size for 80% power to reject null hypothesis of 75% retention), g) Power vs sample size to detect

differences in 12-month treatment rates in our 3-arm trial, assuming treatment percentages of 4.5% vs. 15.5% vs. 20.3% with 1:1:1 randomisation to Usual Care vs. Active Control FRAX-GP vs. PHOENIX Intervention respectively, and type 1 error = 0.05

84x132mm (300 x 300 DPI)

Appendix 1

NHS Cambridge University Hospitals NHS Foundation Trust

UNIVERSITY OF CAMBRIDGE

PHOENIX

Picking up Hidden Osteoporosis Effectively during Normal CT Imaging without additional X-rays

For peer review only

Osteoporosis is a condition that makes bones become fragile and break more easily, especially in the spine and hips. Osteoporosis can be treated. We would like to diagnose it earlier and are running a study called PHOENIX-f to see if this can be done using the scan that you are having today. There are no extra scans, other tests or treatment required to join the study.

If you are interested in being part of the study, please turn over the page and read on

If you do not wish to take part in the study, can you please tell us why in the box below? This would be really useful for us to know, but you do not have to tell us.

Now please return this form to the PHOENIX-f returns box

Thank you for your co-operation

For peer review only - http://bmjopen.bmj.com/site/about/guidelines.xhtml

By completing this form, we understand that you are happy for one of our research team to talk to you about the study.

Your scan taken today can also be used to measure your bone health.

We can't use every scan, so please answer the following 3 questions to see if we can use yours.

Please circle your answer

**Do you take medication for your bones once a week that require you to sit or stand upright?**	**Y**	**N**
**Do you have a yearly infusion (drip) or injection for your bones?**	**Y**	**N**
**Do you have a hip replacement? Have you ever had a hip operation?**	**Y**	**N**

If you answered 'Yes' to any of the questions above unfortunately we will be unable to use your CT scan today for PHOENIX-f.

Please return this form to the PHOENIX-f returns box.
Thank you for your co-operation.

If you have answered 'NO' to ALL the questions above we will be able to review your CT scan for PHOENIX-f.

In order to understand the study more, please read the patient information leaflet. Then, if you are interested in taking part in PHOENIX to measure your bone health, please fill in the questionnaire overleaf and complete the consent form

For peer review only

For peer review only - http://bmjopen.bmj.com/site/about/guidelines.xhtml

PHOENIX-f Consent Form

Chief Investigator: Dr Ken Poole, Consultant in Rheumatology and Metabolic Bone Disease, Cambridge

Please *initial* boxes below, then sign and date the form and add your address ***Initials***

1. I confirm that I have read and understood the patient information for the study. ☐
2. I understand that my participation is voluntary and that I am free to withdraw at any time without giving any reason, without my medical care or legal rights being affected. ☐
3. If I decide to withdraw from the trial I agree to data already collected to be used for Phoenix-f. ☐
4. I give permission for the study researchers to have access to my scan and relevant NHS databases in order to conduct this research ☐
5. I agree for my personal details and relevant medical information to be sent from my hospital to the study team at Addenbrooke's Hospital for the purpose of the research. I understand that scans have identifiable data and will be kept securely at Addenbrooke's Hospital and will only be accessed by the research team ☐
6. I agree to the research team contacting my GP to notify them of my participation in the study and any results ☐
7. I give permission to be contacted by post or phone by the study team at Addenbrooke's Hospital. I understand that this will be to send me 2 questionnaires and may include an invitation to talk to a researcher about my experience of the study ☐
8. I agree to take part in the above study. ☐

Mr / Mrs / Ms / Dr (please circle as appropriate)

....

Please enter first and family name above

…………………………………………………………. *Date:* ………………………….

Your Signature

Date of Birth: .... *Height:* .... *Weight:* ....

NHS number: ....

Why are you having a scan today? ....

The following questions are required for accurate bone health calculation:

Your country of origin: .... *Ethnicity:* ....

PLEASE TURN OVER PAGE TO COMPLETE THIS FORM

For peer review only - http://bmjopen.bmj.com/site/about/guidelines.xhtml
For peer review only

4

BONE HEALTH QUESTIONNAIRE	Yes	No	Not Sure
Have you broken any bones since you turned 40?			
If Yes, which bones?			
Did your mother or father ever break a hip?			
Do you currently smoke?			
Do you take steroid tablets now (eg: 'prednisolone'), or have you taken steroid tablets in the past, for longer than three months at any time?			
Have you been diagnosed with rheumatoid arthritis?			
Have you been diagnosed with any of the following?<br>**(Tick 'yes' if any of the following apply)**<br>• Premature menopause (before 45yrs of age)<br>• Insulin dependent diabetes<br>• Brittle bone disease, Ankylosing spondylitis<br>• Overactive thyroid<br>• Low testosterone level (men only)<br>• Chronic malnutrition or malabsorption eg: anorexia or coeliac disease<br>• Chronic liver disease			
Do you drink three or more units of alcohol a **day**, on average? (That's 1 1/2 pints of beer or 3 glasses of wine or 3 short measures of spirits)			

Your Address (for questionnaires):

....

....

....

....

Post Code: .... **Your Telephone no:** ....

Your GP Name and address:

....

....

Now please return this form to the PHOENIX-f returns box. Thank you for your co-operation

PHOENIX Methodology paper_2021_04_29_ submission 4

For peer review only

PHOENIX-f Clinical Decision Tree *part 1*

Initial steps

1. Retrieve and visually inspect CT images for any vertebral fractures with AP and lateral localiser views (using Mindways QCT PRO SlicePick for 6-point morphometry).

2. Measure CTXA Hip and/or Spine BMD with QCT PRO, noting Femoral neck & Hip BMD results (in g/cm2) and/or Average L1-L3 Spine BMD results (in mg/cm3).

3. Recalculate FRAX online using the Mindways Femoral neck/Hip BMD (g/cm2) value & change 'Previous Fracture' to 'Yes' if a Grade 2 or 3 fracture is found at step 1.

4. If only one (Hip or Spine) result is available, follow the GP Advice Clinical Decision Tree here to generate automatic GP Advice text.

5. If both Hip BMD and Spine BMD results are available, CTXA Femoral neck/Hip results usually dominate, and the following generic Spine 3D QCT GP advice box text is automatically added, "See CTXA Hip Bone Mineral Densitometry report for clinical interpretation." In GP Advice box add the Spine BMD value and definitions of Normal/ Osteopaenia/Osteoporosis.

6. Only if Spine BMD value is <80mg/cm3 AND NOGG advice is GREEN (after entering Femoral neck/Hip BMD into FRAX) does a Spine result dominate. In this case, the following generic CTXA Hip GP advice box text is automatically added, "Both hip and spine results are available. For this patients' clinical interpretation, please see their associated Spine 3D QCT BMD result".

GP advice text using *Mindways QCT Pro Spine L1-3 BMD value + initial FRAX score/NOGG*

ACR Spine BMD Thresholds

**Abnormal High BMD >200mg/cm^3**	**Normal BMD >120mg/cm^3**		**Osteopenia 120mg/cm^3 ≥ BMD > 80mg/cm^3**		**Osteoporosis BMD ≤ 80mg/cm^3**
	**Initial FRAX Major osteoporotic fracture risk ≤ 20%**	**Initial FRAX Major osteoporotic fracture risk >20%**	**Initial FRAX Major osteoporotic fracture risk ≤ 20%**	**Initial FRAX Major osteoporotic fracture risk >20%**	
This patient had a FRAX score high enough to warrant a BMD assessment which was done using the clinical CT scan. Their BMD of (***) mg/cm3 (*) is higher than expected. High density can be due to degenerative/ genetic effects or for other reasons.	This patient had a FRAX score high enough to warrant a BMD assessment which was done using the clinical CT scan. Their spine BMD >120mg/cm3 (*) is defined as normal bone density by ACR criteria. Unless there is a change in the clinical circumstances, no routine repeat BMD tests are indicated.	This patient had a FRAX score high enough to warrant BMD assessment using the clinical CT scan. Their spine BMD >120mg/cm3 (*) which is defined as normal bone density by ACR criteria. However, there are clinical risk factors which elevate the 10 year future fracture risk (**%).	This patient had a FRAX score high enough to warrant a BMD assessment which was done using the clinical CT scan. Their SPINE BMD of 80-120 mg/cm3 (*) is defined as osteopenia by ACR criteria. There is no current indication for bone active therapy, but the patient may wish to follow the lifestyle advice to maintain bone density (if relevant to patient avoid smoking and excess alcohol intake, and maintain weightbearing exercise.) Please use the online WHO FRAX tool to determine if a standard DXA scan is needed in 3 years.	This patient had a FRAX score high enough to warrant BMD assessment using the clinical CT scan. Their SPINE BMD of 80-120 mg/cm3(*) is defined as osteopenia by ACR criteria. However, there are clinical risk factors which elevate the 10 year future fracture risk above the treatment threshold (**%). Unless contraindicated, consider bisphosphonate treatment.	This patient had a FRAX score high enough (10 year future risk **%) to warrant a BMD assessment using their clinical CT scan. Their SPINE BMD of <80mg/cm3 (*) is defined as osteoporosis by ACR criteria. We suggest investigations to rule out secondary causes (e.g. renal/liver/bone function, low vit D, high PTH, occasionally TTG, paraprotein screen). Then, unless contraindicated, consider oral or intravenous bisphosphonates as first line therapy with sufficient vitamin D & calcium. If relevant to patient, please recommend avoid smoking, excess alcohol intake, & maintain weightbearing exercise. Follow up: Please re-assess fracture risk and indication for treatment after 3-5 years
If grade 2 or 3 vertebral # identified.<br>Include:<br>Please note that a vertebral fracture was identified. Unless this was traumatic/already known, you may wish to investigate further	If grade 2 or 3 vertebral # identified.<br>Include:<br>Please note that a vertebral fracture was identified. Unless this was traumatic/already known, you may wish to investigate further	If grade 2 or 3 vertebral # identified.<br>Include:<br>Please note that a vertebral fracture was identified. Unless this was traumatic/already known, you may wish to investigate further	If grade 2 or 3 vertebral # identified.<br>Include:<br>Please note that a vertebral fracture was identified. Unless this was traumatic/already known, you may wish to investigate further	If grade 2 or 3 vertebral # identified.<br>Include:<br>Please note that a vertebral fracture was identified. Unless this was traumatic/already known, you may wish to investigate further	If grade 2 or 3 vertebral # identified.<br>Include:<br>Please note that a vertebral fracture was identified. Unless this was traumatic/already known, you may wish to investigate further

* Vertebrae analysed
** Major Fracture Risk Score
*** Average BMD value of vertebrae analysed

American College of Radiology

PHOENIX-f Clinical Decision Tree *part 2*

GP advice text using *Mindways QCT Pro CTXA Femoral neck BMD value + BMD-Adjusted FRAX/NOGG*

WHO BMD T-Score Criteria								
**Abnormal High T-Score > 2**	**Normal T-Score > -1**			**Osteopenia -1 ≥ T-Score > -2.5**			**Osteoporosis T-Score ≤ -2.5**	
**Any FRAX/NOGG Threshold**	**Below FRAX /NOGG Treatment Threshold**	**Below FRAX/NOGG Treatment Threshold AND ≥75yrs with prior fragility#/vert #**	**Above FRAX/NOGG Treatment Threshold**	**Below FRAX/NOGG Treatment Threshold**	**Either i) Below FRAX/NOGG Treatment Threshold ≥75yrs with prior fragility#/vert# OR ii) Between -2.0 & -2.50 with prior fragility#/vert#**	**Above FRAX/NOGG Treatment Threshold**	**Below FRAX/NOGG Treatment Threshold**	**Either i) Above FRAX/NOGG Treatment Threshold OR ii) Below treatment threshold with a prior fracture aged ≥75**
This patient has a FRAX score high enough to warrant a BMD assessment which was done using their clinical CT scan. Their *femoral neck/ total hip T-score was (***) which is higher than expected which can have genetic, degenerative or other causes. There is no evidence of osteoporosis	This patient had a FRAX score high enough to warrant a BMD assessment using the clinical CT scan. Their *femoral neck/total hip T-score was >-1 which is defined as normal hip bone density by WHO guidelines (FRAX score **%). Unless there is a change in the clinical circumstances, no routine repeat BMD tests are indicated.	This patient had a FRAX score high enough to warrant a BMD assessment using the clinical CT scan. Their *femoral neck/ total hip T-score was >-1 which is defined as normal hip bone density by WHO guidelines (FRAX score **%). Please note National Guidelines recommend bisphosphonate treatment in the majority of elderly people with a prior fragility fracture regardless of calculated risk. If relevant to patient, recommend avoid smoking, excess alcohol intake, & maintain weightbearing exercise.	This patient had a FRAX score high enough to warrant a BMD assessment using the clinical CT scan. Their *femoral neck/total hip T-score was >-1 which is defined as normal hip bone density by WHO guidelines. However, there are clinical risk factors which elevate the 10 year future fracture risk (FRAX score **%).	This patient had a FRAX score high enough to warrant BMD assessment using the clinical CT scan. Their *femoral neck/ total hip T-score was between -1 and -2.5 which is defined as osteopenia, or low normal bone density, by WHO guidelines. (FRAX score **%). There is no current indication for bone active therapy, but the patient may wish to follow the lifestyle advice to maintain bone density (if relevant to patient avoid smoking and excess alcohol intake, and maintain weightbearing exercise.) Please use the online WHO FRAX tool to determine if a standard DXA scan is needed in 3 years.	This patient had a FRAX score high enough to warrant BMD assessment using the clinical CT scan. Their *femoral neck/total hip T-score was between -1 and > -2.5 which is defined as osteopenia, or low normal bone density, by WHO guidelines (FRAX Score **%). Please note National Guidelines recommend bisphosphonate treatment in the majority of older people with a prior fragility fracture regardless of calculated risk. Unless contraindicated consider bisphosphonate treatment. If relevant to patient, recommend avoid smoking, excess alcohol intake, & maintain weightbearing exercise.	This patient had a FRAX score high enough to warrant BMD assessment using the clinical CT scan. Their *femoral neck/total hip T-score was between -1 and > -2.5 which is defined as osteopenia, or low normal bone density, by WHO guidelines. However, there are clinical risk factors which elevate the 10 year future fracture risk above the treatment threshold (FRAX Score **%). Unless contraindicated consider bisphosphonate treatment. If relevant to patient, recommend avoid smoking, excess alcohol intake, & maintain weightbearing exercise.	This patient's FRAX score was high enough to warrant BMD assessment using their clinical CT scan. Their *femoral neck/total hip T-score was osteoporotic (FRAX score **%). While the scan indicates osteoporosis at the hip, treatment is not automatically recommended (by National Guidelines) at this level of 10-year risk. Suggest investigations to rule out secondary causes (e.g renal/liver/bone function, occasionally TTG, paraprotein screen) The patient may wish to follow the lifestyle advice to maintain bone density (if relevant to patient avoid smoking and excess alcohol intake, and maintain weightbearing exercise.)	This patient's FRAX score was high enough to warrant BMD assessment using their clinical CT scan. Their *femoral neck/ total hip T-score was osteoporotic (FRAX score **%). Suggest investigations to rule out secondary causes (e.g renal/liver/ bone function, occasionally TTG, paraprotein screen). Then, unless contraindicated, consider oral or intravenous bisphosphonates as first line therapy with sufficient vitamin D & calcium. Follow up: Please reassess fracture risk and indication for treatment after 3-5 years
If grade 2 or 3 vertebral # identified<br>Include:<br>Please note that a vertebral fracture was identified, unless this was traumatic/already known, you may wish to investigate further	If grade 2 or 3 vertebral # identified, <75y/o only.<br>Include:<br>Please note that a vertebral fracture was identified, unless this was traumatic/ already known, you may wish to order a spine only DXA scan.	If grade 2 or 3 vertebral # identified.<br>Include:<br>Please note that a vertebral fracture was identified.	If grade 2 or 3 vertebral # identified, <75y/o only.<br>Include:<br>Please note that a vertebral fracture was identified, unless this was traumatic/ already known, you may wish to order a spine only DXA scan."	If grade 2 or 3 vertebral # identified, <75y/o only.<br>Include:<br>Please note that a vertebral fracture was identified, unless this was traumatic/already known, you may wish to order a spine only DXA scan.	If grade 2 or 3 vertebral # identified<br>Include:<br>Please note that a vertebral fracture was identified.	If grade 2 or 3 vertebral # identified<br>Include:<br>Please note that a vertebral fracture was identified.	If grade 2 or 3 vertebral # identified, <75y/o only<br>Include:<br>Please note that a vertebral fracture was identified.	If grade 2 or 3 vertebral # identified<br>Include:<br>Please note that a vertebral fracture was identified.

* Where lowest BMD T-Score was at either the Femoral Neck or Total Hip

** Value of either the Major FRAX score OR the Hip Fracture FRAX score when this value is above the FRAX/NOGG treatment threshold AND Major is below the threshold.

*** Value of the BMD T-Score

Reporting checklist for protocol of a clinical trial.

Based on the SPIRIT guidelines.

Instructions to authors

Complete this checklist by entering the page numbers from your manuscript where readers will find each of the items listed below.

Your article may not currently address all the items on the checklist. Please modify your text to include the missing information. If you are certain that an item does not apply, please write "n/a" and provide a short explanation.

Upload your completed checklist as an extra file when you submit to a journal.

In your methods section, say that you used the SPIRITreporting guidelines, and cite them as:

Chan A-W, Tetzlaff JM, Gøtzsche PC, Altman DG, Mann H, Berlin J, Dickersin K, Hróbjartsson A, Schulz KF, Parulekar WR, Krleža-Jerić K, Laupacis A, Moher D. SPIRIT 2013 Explanation and Elaboration: Guidance for protocols of clinical trials. BMJ. 2013;346:e7586

		Reporting Item	Page Number
**Administrative information**			
Title	#1	Descriptive title identifying the study design, population, interventions, and, if applicable, trial acronym	1

For peer review only

Trial registration	#2a	Trial identifier and registry name. If not yet registered, name of intended registry	1
Trial registration: data set	#2b	All items from the World Health Organization Trial Registration Data Set	1
Protocol version	#3	Date and version identifier	1
Funding	#4	Sources and types of financial, material, and other support	10
Roles and responsibilities: contributorship	#5a	Names, affiliations, and roles of protocol contributors	2
Roles and responsibilities: sponsor contact information	#5b	Name and contact information for the trial sponsor	2
Roles and responsibilities: sponsor and funder	#5c	Role of study sponsor and funders, if any, in study design; collection, management, analysis, and interpretation of data; writing of the report; and the decision to submit the report for publication, including whether they will have ultimate authority over any of these activities	2
Roles and responsibilities: committees	#5d	Composition, roles, and responsibilities of the coordinating centre, steering committee, endpoint adjudication committee, data management team, and	5

For peer review only

		other individuals or groups overseeing the trial, if applicable (see Item 21a for data monitoring committee)	
**Introduction**			
Background and rationale	#6a	Description of research question and justification for undertaking the trial, including summary of relevant studies (published and unpublished) examining benefits and harms for each intervention	3
Background and rationale: choice of comparators	#6b	Explanation for choice of comparators	5
Objectives	#7	Specific objectives or hypotheses	3
Trial design	#8	Description of trial design including type of trial (eg, parallel group, crossover, factorial, single group), allocation ratio, and framework (eg, superiority, equivalence, non-inferiority, exploratory)	4
**Methods: Participants, interventions, and outcomes**			
Study setting	#9	Description of study settings (eg, community clinic, academic hospital) and list of countries where data will be collected. Reference to where list of study sites can be obtained	3

For peer review only

Eligibility criteria	#10	Inclusion and exclusion criteria for participants. If applicable, eligibility criteria for study centres and individuals who will perform the interventions (eg, surgeons, psychotherapists)	7
Interventions: description	#11a	Interventions for each group with sufficient detail to allow replication, including how and when they will be administered	5
Interventions: modifications	#11b	Criteria for discontinuing or modifying allocated interventions for a given trial participant (eg, drug dose change in response to harms, participant request, or improving / worsening disease)	n/a
Interventions: adherance	#11c	Strategies to improve adherence to intervention protocols, and any procedures for monitoring adherence (eg, drug tablet return; laboratory tests)	n/a
Interventions: concomitant care	#11d	Relevant concomitant care and interventions that are permitted or prohibited during the trial	7
Outcomes	#12	Primary, secondary, and other outcomes, including the specific measurement variable (eg, systolic blood pressure), analysis metric (eg, change from baseline, final value, time to event), method of aggregation (eg, median, proportion), and time point for each outcome. Explanation of the clinical relevance of chosen efficacy and harm outcomes is strongly recommended	7

For peer review only

Participant timeline	#13	Time schedule of enrolment, interventions (including any run-ins and washouts), assessments, and visits for participants. A schematic diagram is highly recommended (see Figure)	n/a
Sample size	#14	Estimated number of participants needed to achieve study objectives and how it was determined, including clinical and statistical assumptions supporting any sample size calculations	7
Recruitment	#15	Strategies for achieving adequate participant enrolment to reach target sample size	n/a
**Methods: Assignment of interventions (for controlled trials)**			
Allocation: sequence generation	#16a	Method of generating the allocation sequence (eg, computer-generated random numbers), and list of any factors for stratification. To reduce predictability of a random sequence, details of any planned restriction (eg, blocking) should be provided in a separate document that is unavailable to those who enrol participants or assign interventions	9
Allocation concealment mechanism	#16b	Mechanism of implementing the allocation sequence (eg, central telephone; sequentially numbered, opaque,	n/a

For peer review only

		sealed envelopes), describing any steps to conceal the sequence until interventions are assigned	
Allocation: implementation	#16c	Who will generate the allocation sequence, who will enrol participants, and who will assign participants to interventions	9
Blinding (masking)	#17a	Who will be blinded after assignment to interventions (eg, trial participants, care providers, outcome assessors, data analysts), and how	n/a
Blinding (masking): emergency unblinding	#17b	If blinded, circumstances under which unblinding is permissible, and procedure for revealing a participant's allocated intervention during the trial	n/a
**Methods: Data collection, management, and analysis**			
Data collection plan	#18a	Plans for assessment and collection of outcome, baseline, and other trial data, including any related processes to promote data quality (eg, duplicate measurements, training of assessors) and a description of study instruments (eg, questionnaires, laboratory tests) along with their reliability and validity, if known. Reference to where data collection forms can be found, if not in the protocol	9

For peer review only

Data collection plan: retention	#18b	Plans to promote participant retention and complete follow-up, including list of any outcome data to be collected for participants who discontinue or deviate from intervention protocols	n/a
Data management	#19	Plans for data entry, coding, security, and storage, including any related processes to promote data quality (eg, double data entry; range checks for data values). Reference to where details of data management procedures can be found, if not in the protocol	n/a
Statistics: outcomes	#20a	Statistical methods for analysing primary and secondary outcomes. Reference to where other details of the statistical analysis plan can be found, if not in the protocol	9
Statistics: additional analyses	#20b	Methods for any additional analyses (eg, subgroup and adjusted analyses)	9
Statistics: analysis population and missing data	#20c	Definition of analysis population relating to protocol non-adherence (eg, as randomised analysis), and any statistical methods to handle missing data (eg, multiple imputation)	9

Methods: Monitoring

Data monitoring: formal committee	#21a	Composition of data monitoring committee (DMC); summary of its role and reporting structure; statement of whether it is independent from the sponsor and competing interests; and reference to where further	n/a

For peer review only

		details about its charter can be found, if not in the protocol. Alternatively, an explanation of why a DMC is not needed	
Data monitoring: interim analysis	#21b	Description of any interim analyses and stopping guidelines, including who will have access to these interim results and make the final decision to terminate the trial	n/a
Harms	#22	Plans for collecting, assessing, reporting, and managing solicited and spontaneously reported adverse events and other unintended effects of trial interventions or trial conduct	n/a
Auditing	#23	Frequency and procedures for auditing trial conduct, if any, and whether the process will be independent from investigators and the sponsor	n/a
**Ethics and dissemination**			
Research ethics approval	#24	Plans for seeking research ethics committee / institutional review board (REC / IRB) approval	10
Protocol amendments	#25	Plans for communicating important protocol modifications (eg, changes to eligibility criteria, outcomes, analyses) to relevant parties (eg, investigators, REC / IRBs, trial participants, trial registries, journals, regulators)	1

For peer review only

Consent or assent	#26a	Who will obtain informed consent or assent from potential trial participants or authorised surrogates, and how (see Item 32)	5
Consent or assent: ancillary studies	#26b	Additional consent provisions for collection and use of participant data and biological specimens in ancillary studies, if applicable	n/a
Confidentiality	#27	How personal information about potential and enrolled participants will be collected, shared, and maintained in order to protect confidentiality before, during, and after the trial	5
Declaration of interests	#28	Financial and other competing interests for principal investigators for the overall trial and each study site	10
Data access	#29	Statement of who will have access to the final trial dataset, and disclosure of contractual agreements that limit such access for investigators	10
Ancillary and post trial care	#30	Provisions, if any, for ancillary and post-trial care, and for compensation to those who suffer harm from trial participation	n/a
Dissemination policy: trial results	#31a	Plans for investigators and sponsor to communicate trial results to participants, healthcare professionals, the public, and other relevant groups (eg, via publication, reporting in results databases, or other data sharing arrangements), including any publication restrictions	10

For peer review only

Dissemination policy: authorship	#31b	Authorship eligibility guidelines and any intended use of professional writers	n/a
Dissemination policy: reproducible research	#31c	Plans, if any, for granting public access to the full protocol, participant-level dataset, and statistical code	10

Appendices

Informed consent materials	#32	Model consent form and other related documentation given to participants and authorised surrogates	12
Biological specimens	#33	Plans for collection, laboratory evaluation, and storage of biological specimens for genetic or molecular analysis in the current trial and for future use in ancillary studies, if applicable	n/a

The SPIRIT Explanation and Elaboration paper is distributed under the terms of the Creative Commons Attribution License CC-BY-NC. This checklist was completed on 16. February 2021 using https://www.goodreports.org/, a tool made by the EQUATOR Network in collaboration with Penelope.ai

For peer review only
